# Supplementary material for: Recyclable and Stable α-Methylproline-Derived Chiral Ligands for the Chemical Dynamic Kinetic Resolution of free C,N-Unprotected α-Amino Acids
Source: Molecules. 2019 Jun 13;24(12):2218. doi: 10.3390/molecules24122218 (PMC6630268; doi:10.3390/molecules24122218)

Supplementary Materials

Recyclable and Stable α-Methylproline-Derived Chiral Ligands for the Chemical Dynamic Kinetic Resolution of free *C,N*-Unprotected α-Amino Acids

Shuangjie Shu †, Liang Zhao †, Shengbin Zhou, Chenglin Wu, Hong Liu * and Jiang Wang *

State Key Laboratory of Drug Research, Shanghai Institute of Materia Medica, Chinese Academy of Sciences, 555 Zu Chong Zhi Road, Shanghai 201203, China; shushuangjie@126.com (S.S.); frankzl@163.com (L.Z.); stbin_06@163.com (S.Z.); wucl1990@mail.ustc.edu.cn (C.W.);

† These authors contributed equally to this work.

* Correspondence: hliu@simm.ac.cn (H.L.); jwang@simm.ac.cn (J.W.); Tel.: +86-21-5080-7042 (H.L.); +86-21-50806600-5418 (J.W.)

Academic Editor: Derek J. McPhee

Contents

(A) Table S1: HPLC Spectra for dr Determination of (*S*, 2*S*)-6……………………………………S3

(B) Table S2: HPLC Spectra for dr Determination of (*R*, 2*R*)-6……………………………………...S3

(C) Figure S1: HPLC Spectra for ee Determination…………………………………….................S21

(D) Figure S2: Copies of ^{1}H NMR and ^{13}C NMR Spectra for the Products………………………..S22

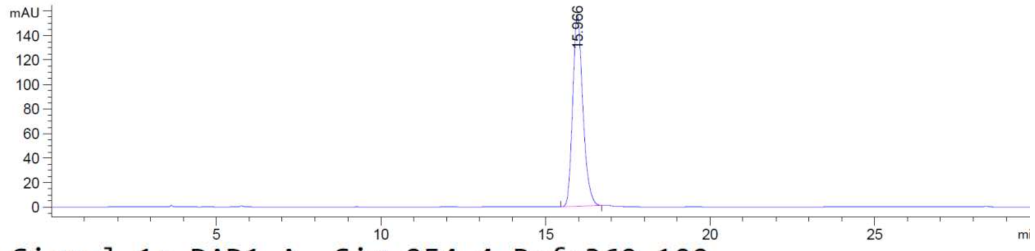

Signal 1: DAD1 A, Sig=254,4 Ref=360,100

Peak #	RetTime [min]	Type	Width [min]	Area [mAU*s]	Height [mAU]	Area %
1	15.966	BB	0.3291	3361.41943	156.19789	100.0000
Totals :				3361.41943	156.19789	

Figure S1. HPLC Spectra for dr Determination. **(S,2S)-6a**. The dr was determined by LC-MS with binary pump, photodiode array detector (DAD), using Eclipse XDB-C18 column (250 × 4.6 mm, 5 μm) (CH_3CN/H_2O = 65:35, flow rate 1.0 mL/min, λ = 254 nm), t_{major} = 15.966 min, t_{minor} = not found, dr > 99:1.

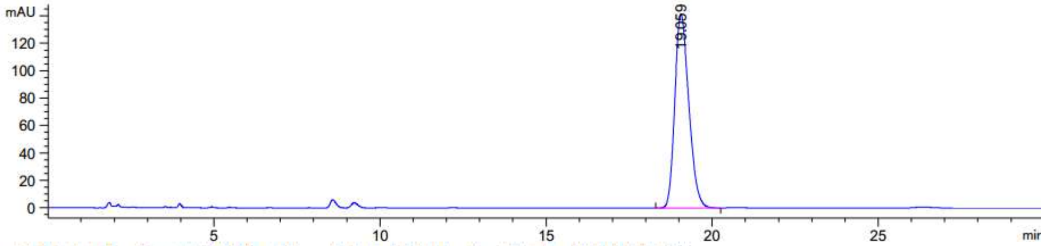

Signal 1: DAD1 A, Sig=254,4 Ref=360,100

Peak #	RetTime [min]	Type	Width [min]	Area [mAU*s]	Height [mAU]	Area %
1	19.059	BB	0.4536	4148.16406	141.66849	100.0000
Totals :				4148.16406	141.66849	

Figure S2. HPLC Spectra for dr Determination. **(S,2S)-6b.** The dr was determined by LC-MS with binary pump, photodiode array detector (DAD), using Eclipse XDB-C18 column (250 × 4.6 mm, 5 μm) (CH_3CN/H_2O = 65:35, flow rate 1.0 mL/min, λ = 254 nm), t_{major} = 19.059 min, t_{minor} = not found, dr > 99:1.

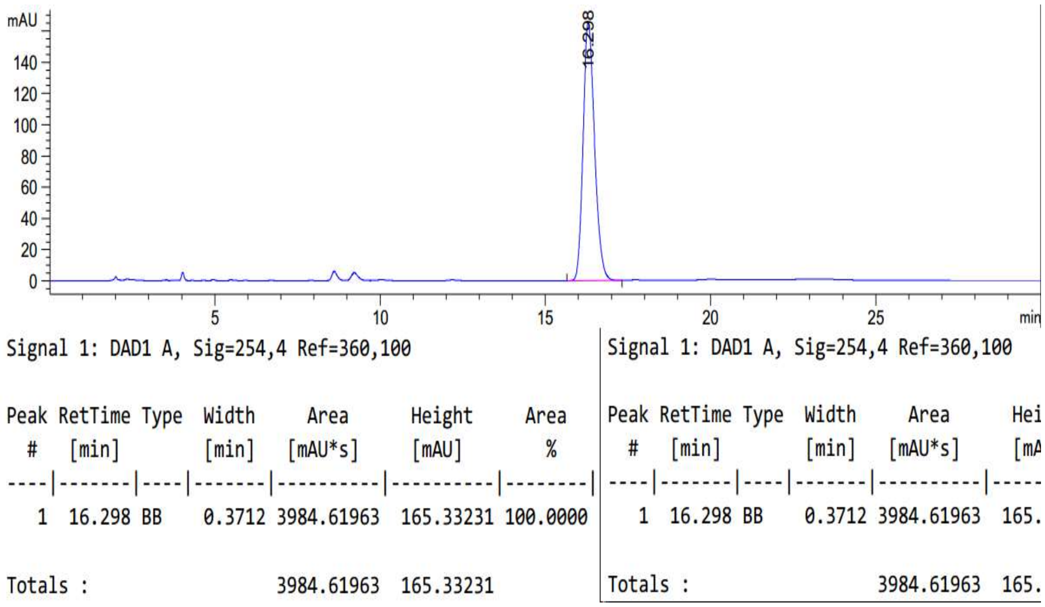

Signal 1: DAD1 A, Sig=254,4 Ref=360,100

Peak #	RetTime [min]	Type	Width [min]	Area [mAU*s]	Height [mAU]	Area %
1	16.298	BB	0.3712	3984.61963	165.33231	100.0000
Totals :				3984.61963	165.33231	

Signal 1: DAD1 A, Sig=254,4 Ref=360,100

Peak #	RetTime [min]	Type	Width [min]	Area [mAU*s]	Hei [mA
1	16.298	BB	0.3712	3984.61963	165.
Totals :				3984.61963	165.

Figure S3 HPLC Spectra for dr Determination. **(S,2S)-6c.** The dr was determined by LC-MS with binary pump, photodiode array detector (DAD), using Eclipse XDB-C18 column (250 × 4.6 mm, 5 μm) (CH3CN/H2O = 65:35, flow rate 1.0 mL/min, λ = 254 nm), tmajor = 16.298 min, tminor = not found, dr > 99:1.

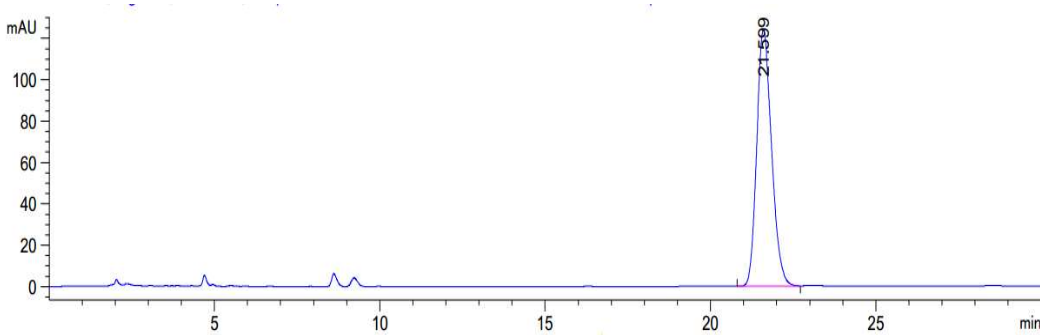

Signal 1: DAD1 A, Sig=254,4 Ref=360,100

Peak #	RetTime [min]	Type	Width [min]	Area [mAU*s]	Height [mAU]	Area %
1	21.599	BB	0.4818	3867.19165	124.00460	100.0000
Totals :				3867.19165	124.00460	

Signal 1: DAD1 A, Sig=254,4 Ref=360,100

Peak #	RetTime [min]	Type	Width [min]	Area [mAU*s]	He: [m/
1	21.599	BB	0.4818	3867.19165	124.
Totals :				3867.19165	124.

Figure S4. HPLC Spectra for dr Determination. **(S,2S)-6d.** The dr was determined by LC-MS with binary pump, photodiode array detector (DAD), using Eclipse XDB-C18 column (250 × 4.6 mm, 5 μm) (CH3CN/H2O = 65:35, flow rate 1.0 mL/min, λ = 254 nm), tmajor =21.599 min, tminor = not found, dr > 99:1.

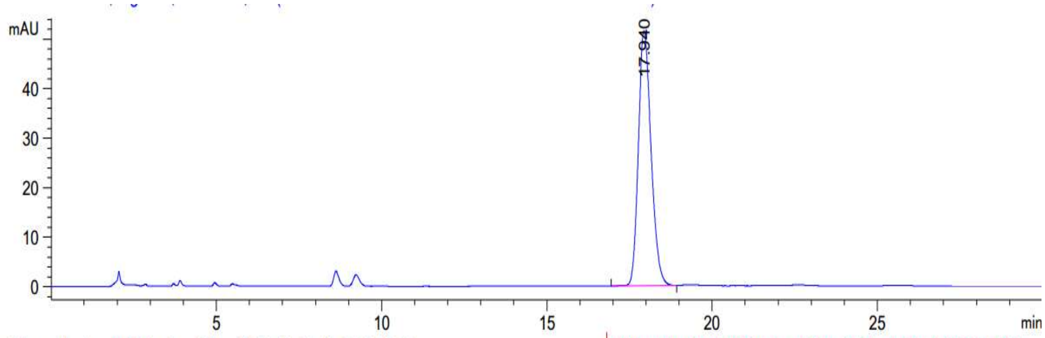

Signal 1: DAD1 A, Sig=254,4 Ref=360,100

Peak #	RetTime [min]	Type	Width [min]	Area [mAU*s]	Height [mAU]	Area %
1	17.940	BB	0.4115	1374.31299	51.48024	100.0000
Totals :				1374.31299	51.48024	

Signal 1: DAD1 A, Sig=254,4 Ref=360,100

Peak #	RetTime [min]	Type	Width [min]	Area [mAU*s]	He [m
1	17.940	BB	0.4115	1374.31299	51
Totals :				1374.31299	51

Figure S4. HPLC Spectra for dr Determination. **(S,2S)-6e**. The dr was determined by LC-MS with binary pump, photodiode array detector (DAD), using Eclipse XDB-C18 column (250 × 4.6 mm, 5 μm) (CH_3CN/H_2O = 65:35, flow rate 1.0 mL/min, λ = 254 nm), tmajor =17.940 min, tminor = not found, dr > 99:1.

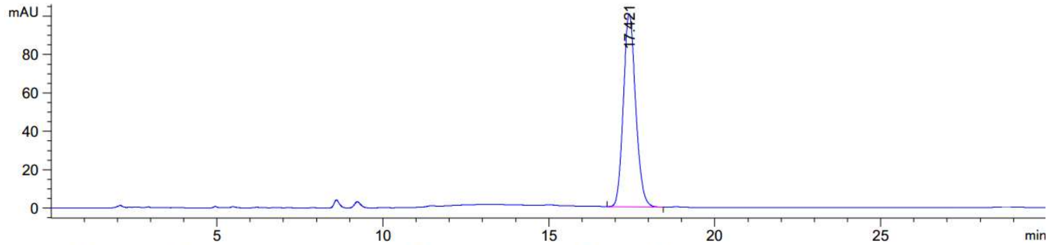

Signal 1: DAD1 A, Sig=254,4 Ref=360,100

Peak #	RetTime [min]	Type	Width [min]	Area [mAU*s]	Height [mAU]	Area %
1	17.421	BB	0.3970	2593.92383	100.53072	100.0000
Totals :				2593.92383	100.53072	

Figure S5. HPLC Spectra for dr Determination. **(S,2S)-6f.** The dr was determined by LC-MS with binary pump, photodiode array detector (DAD), using Eclipse XDB-C18 column (250 × 4.6 mm, 5 μm) (CH_3CN/H_2O = 65:35, flow rate 1.0 mL/min, λ = 254 nm), tmajor =17.421 min, tminor = not found, dr > 99:1.

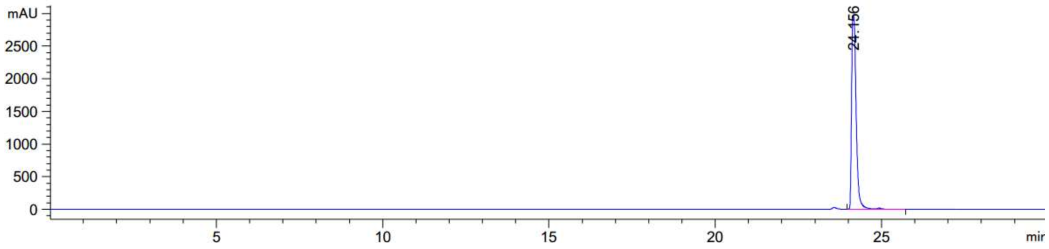

Signal 1: DAD1 A, Sig=254,4 Ref=360,100

Peak #	RetTime [min]	Type	Width [min]	Area [mAU*s]	Height [mAU]	Area %
1	24.156	VV R	0.1424	2.77940e4	2977.50684	100.0000
Totals :				2.77940e4	2977.50684	

Figure S6. HPLC Spectra for dr Determination. **(S,2S)-6g.** The dr was determined by LC-MS with binary pump, photodiode array detector (DAD), using Eclipse XDB-C18 column (250 × 4.6 mm, 5 μm) (CH3CN/H2O = 65:35, flow rate 1.0 mL/min, λ = 254 nm), tmajor =24.156 min, tminor = not found, dr > 99:1.

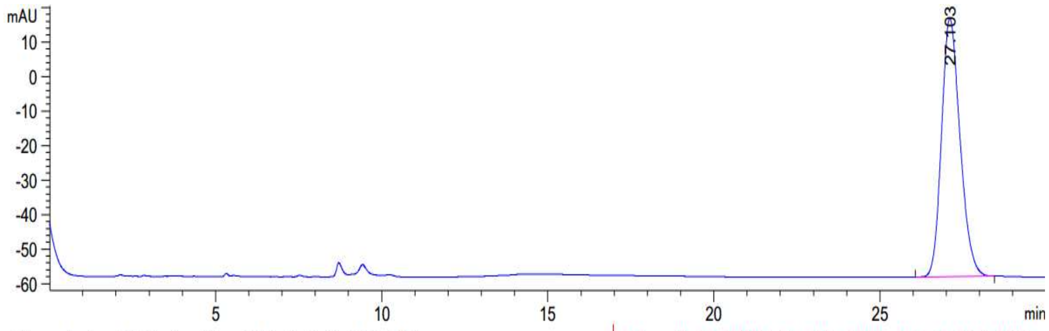

Signal 1: DAD1 A, Sig=254,4 Ref=360,100

Peak #	RetTime [min]	Type	Width [min]	Area [mAU*s]	Height [mAU]	Area %
1	27.103	BB	0.5965	2902.74463	74.83443	100.0000
Totals :				2902.74463	74.83443	

Signal 1: DAD1 A, Sig=254,4 Ref=360,100

Peak #	RetTime [min]	Type	Width [min]	Area [mAU*s]	He [r
1	27.103	BB	0.5965	2902.74463	74
Totals :				2902.74463	74

Figure S7. HPLC Spectra for dr Determination. **(S,2S)-6h.** The dr was determined by LC-MS with binary pump, photodiode array detector (DAD), using Eclipse XDB-C18 column (250 × 4.6 mm, 5 μm) (CH3CN/H2O = 65:35, flow rate 1.0 mL/min, λ = 254 nm), tmajor =27.103 min, tminor = not found, dr > 99:1.

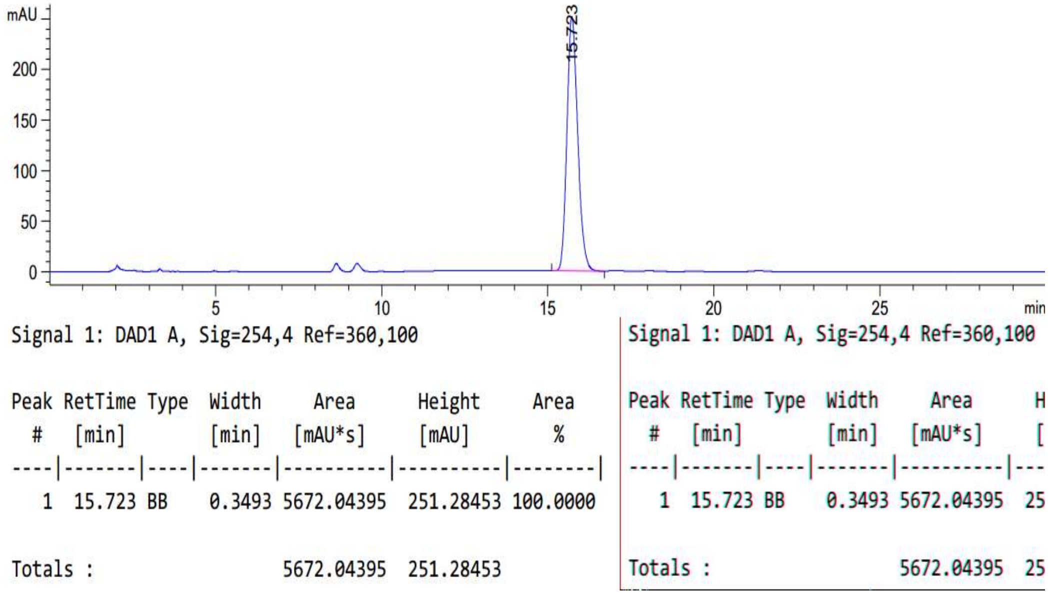

Signal 1: DAD1 A, Sig=254,4 Ref=360,100

Peak #	RetTime [min]	Type	Width [min]	Area [mAU*s]	Height [mAU]	Area %
1	15.723	BB	0.3493	5672.04395	251.28453	100.0000
Totals :				5672.04395	251.28453	

Signal 1: DAD1 A, Sig=254,4 Ref=360,100

Peak #	RetTime [min]	Type	Width [min]	Area [mAU*s]	H [
1	15.723	BB	0.3493	5672.04395	25
Totals :				5672.04395	25

Figure S8. HPLC Spectra for dr Determination. **(S,2S)-6i.** The dr was determined by LC-MS with binary pump, photodiode array detector (DAD), using Eclipse XDB-C18 column (250 × 4.6 mm, 5 μm) (CH3CN/H2O = 65:35, flow rate 1.0 mL/min, λ = 254 nm), tmajor =15.723 min, tminor = not found, dr > 99:1.

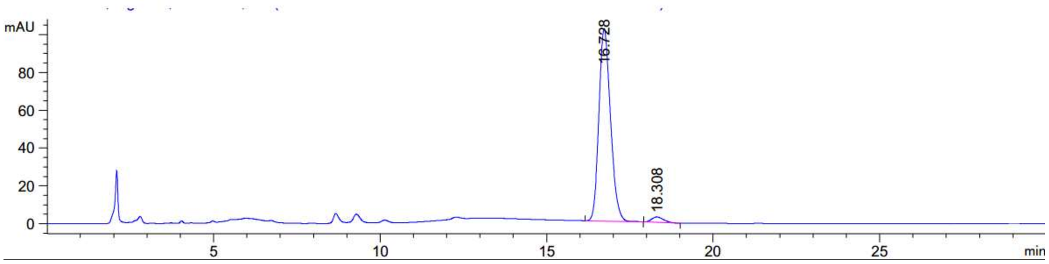

Signal 1: DAD1 A, Sig=254,4 Ref=360,100

Peak #	RetTime [min]	Type	Width [min]	Area [mAU*s]	Height [mAU]	Area %
1	16.728	BB	0.3730	2470.32056	101.83247	97.1254
2	18.308	BB	0.3911	73.11334	2.89011	2.8746
Totals :				2543.43390	104.72258	

Figure S9. HPLC Spectra for dr Determination. **(S,2S)-6j.** The dr was determined by LC-MS with binary pump, photodiode array detector (DAD), using Eclipse XDB-C18 column (250 × 4.6 mm, 5 μm) (CH3CN/H2O = 65:35, flow rate 1.0 mL/min, λ = 254 nm), tmajor =16.728 min, tminor = 18.306, dr = 97:3.

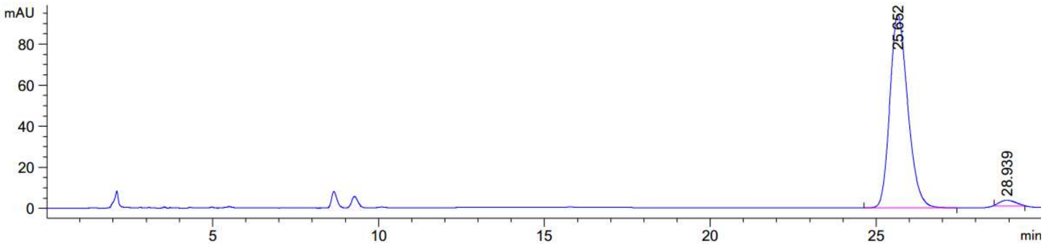

Signal 1: DAD1 A, Sig=254,4 Ref=360,100

Peak #	RetTime [min]	Type	Width [min]	Area [mAU*s]	Height [mAU]	Area %
1	25.652	BB	0.5764	3521.20605	94.14336	97.5461
2	28.939	MM R	0.5481	88.58088	2.69367	2.4539
Totals :				3609.78693	96.83703	

Figure S10. HPLC Spectra for dr Determination. **(S,2S)-6k.** The dr was determined by LC-MS with binary pump, photodiode array detector (DAD), using Eclipse XDB-C18 column (250 × 4.6 mm, 5 μm) (CH3CN/H2O = 65:35, flow rate 1.0 mL/min, λ = 254 nm), tmajor =25.652 min, tminor = 28.939, dr = 98:2.

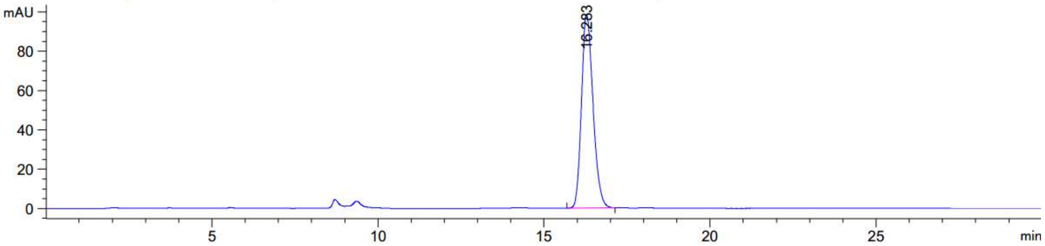

Signal 1: DAD1 A, Sig=254,4 Ref=360,100

Peak #	RetTime [min]	Type	Width [min]	Area [mAU*s]	Height [mAU]	Area %
1	16.283	BB	0.3707	2369.86108	98.49016	100.0000
Totals :				2369.86108	98.49016	

Figure S11. HPLC Spectra for dr Determination. **(S,2S)-6l.** The dr was determined by LC-MS with binary pump, photodiode array detector (DAD), using Eclipse XDB-C18 column

(250 × 4.6 mm, 5 μm) ($CH3CN/H2O$ = 65:35, flow rate 1.0 mL/min, λ = 254 nm), tmajor =16.283 min, tminor = not found, dr > 99:1.

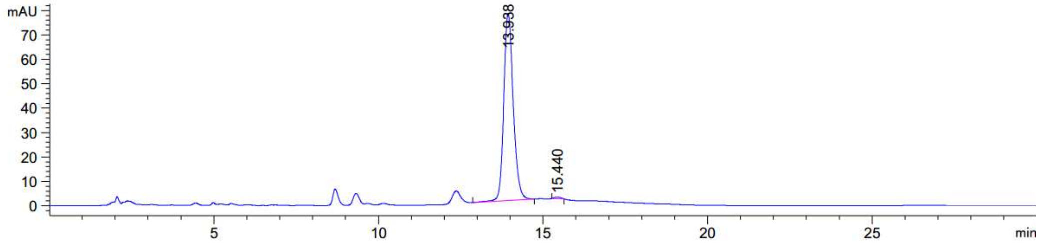

Signal 1: DAD1 A, Sig=254,4 Ref=360,100

Peak #	RetTime [min]	Type	Width [min]	Area [mAU*s]	Height [mAU]	Area %
1	13.938	BB	0.3069	1530.51453	76.67406	99.3870
2	15.440	MM R	0.2291	9.43969	6.86610e-1	0.6130
Totals :				1539.95421	77.36067	

Figure S12. HPLC Spectra for dr Determination. **(S,2S)-6m.** The dr was determined by LC-MS with binary pump, photodiode array detector (DAD), using Eclipse XDB-C18 column (250 × 4.6 mm, 5 μm) ($CH3CN/H2O$ = 65:35, flow rate 1.0 mL/min, λ = 254 nm), tmajor =13.938 min, tminor = 15.440, dr > 99:1.

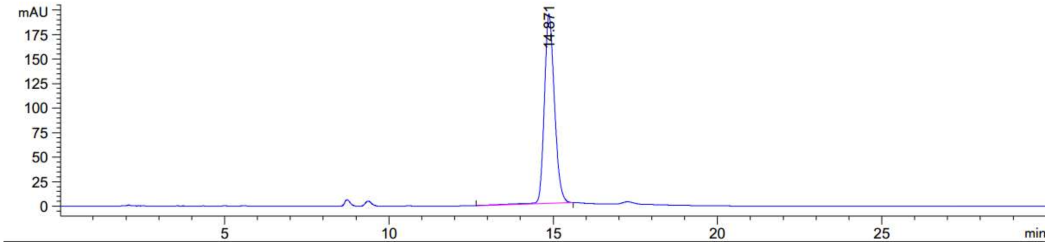

Signal 1: DAD1 A, Sig=254,4 Ref=360,100

Peak #	RetTime [min]	Type	Width [min]	Area [mAU*s]	Height [mAU]	Area %
1	14.871	BB	0.3287	4114.56836	193.00291	100.0000
Totals :				4114.56836	193.00291	

Table S1. HPLC Spectra for dr Determination. **(S,2S)-6n.** The dr was determined by LC-MS with binary pump, photodiode array detector (DAD), using Eclipse XDB-C18 column (250 × 4.6 mm, 5 μm) (CH_3CN/H_2O = 65:35, flow rate 1.0 mL/min, λ = 254 nm), tmajor =14.871 min, tminor = not found, dr > 99:1.

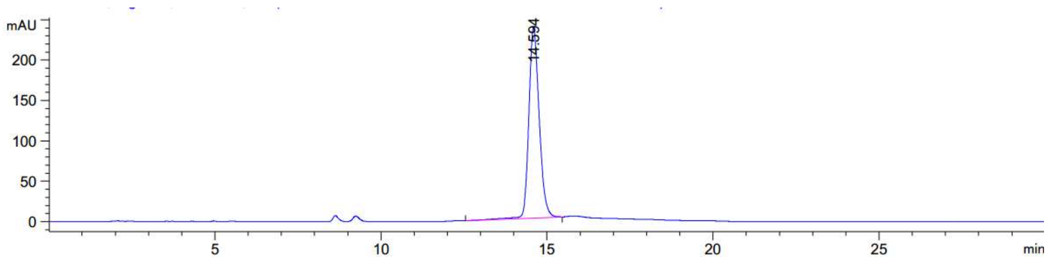

Signal 1: DAD1 A, Sig=254,4 Ref=360,100

Peak #	RetTime [min]	Type	Width [min]	Area [mAU*s]	Height [mAU]	Area %
1	14.594	BB	0.3329	5128.51611	236.61984	100.0000
Totals :				5128.51611	236.61984	

Figure S13. HPLC Spectra for dr Determination. **(S,2S)-6o.** The dr was determined by LC-MS with binary pump, photodiode array detector (DAD), using Eclipse XDB-C18 column (250 × 4.6 mm, 5 μm) (CH_3CN/H_2O = 65:35, flow rate 1.0 mL/min, λ = 254 nm), tmajor =14.594 min, tminor = not found, dr > 99:1.

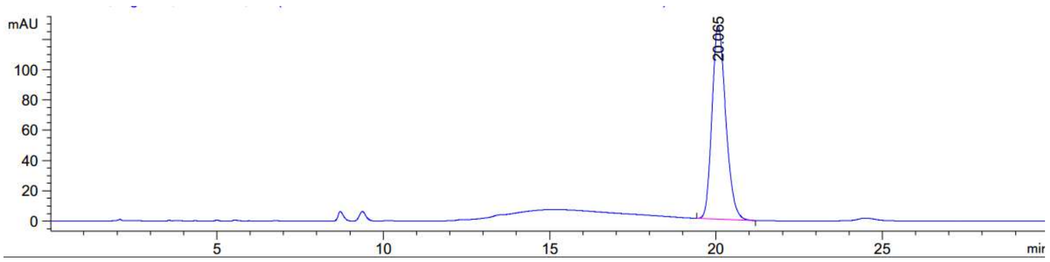

Signal 1: DAD1 A, Sig=254,4 Ref=360,100

Peak #	RetTime [min]	Type	Width [min]	Area [mAU*s]	Height [mAU]	Area %
1	20.065	BB	0.4425	3660.47900	127.65401	100.0000
Totals :				3660.47900	127.65401	

Figure S14. HPLC Spectra for dr Determination. **(S,2S)-6p.** The dr was determined by LC-MS with binary pump, photodiode array detector (DAD), using Eclipse XDB-C18 column (250 × 4.6 mm, 5 μm) ($CH3CN/H2O$ = 65:35, flow rate 1.0 mL/min, λ = 254 nm), tmajor =20.065 min, tminor = not found, dr > 99:1.

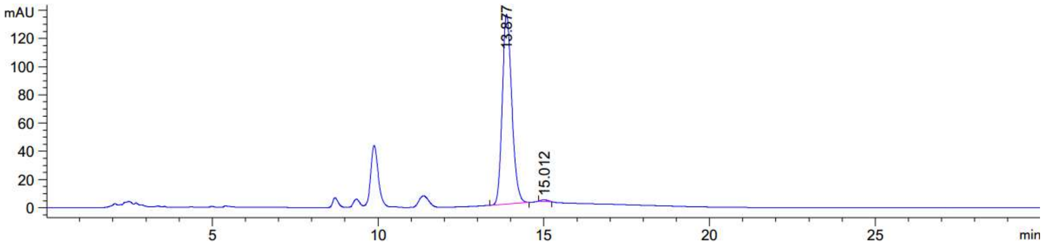

Signal 1: DAD1 A, Sig=254,4 Ref=360,100

Peak #	RetTime [min]	Type	Width [min]	Area [mAU*s]	Height [mAU]	Area %
1	13.877	BB	0.3074	2691.48608	134.53139	99.3861
2	15.012	MM R	0.2451	16.62535	1.13065	0.6139
Totals :				2708.11143	135.66203	

Figure S15. HPLC Spectra for dr Determination. **(S,2S)-6q.** The dr was determined by LC-MS with binary pump, photodiode array detector (DAD), using Eclipse XDB-C18 column (250 × 4.6 mm, 5 μm) ($CH3CN/H2O$ = 65:35, flow rate 1.0 mL/min, λ = 254 nm), tmajor =13.877 min, tminor = 15.012, dr > 99:1.

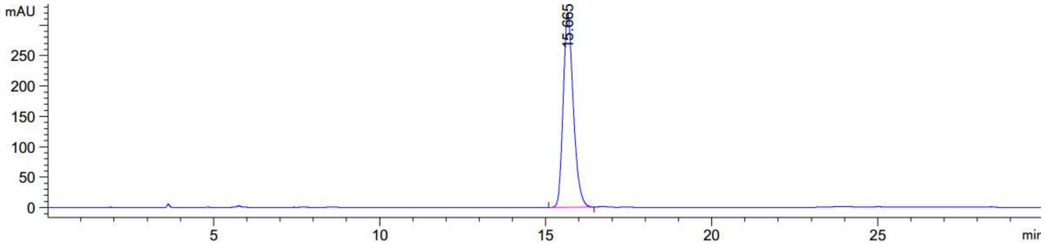

Signal 1: DAD1 A, Sig=254,4 Ref=360,100

Peak #	RetTime [min]	Type	Width [min]	Area [mAU*s]	Height [mAU]	Area %
1	15.665	BB	0.3232	6772.79297	319.65454	100.0000
Totals :				6772.79297	319.65454	

Figure S16. HPLC Spectra for dr Determination. **(*R*,2*R*)-6a.** The dr was determined by LC-MS with binary pump, photodiode array detector (DAD), using Eclipse XDB-C18 column (250 × 4.6 mm, 5 μm) (CH3CN/H2O = 65:35, flow rate 1.0 mL/min, λ = 254 nm), tmajor = 15.665 min, tminor = not found, dr > 99:1.

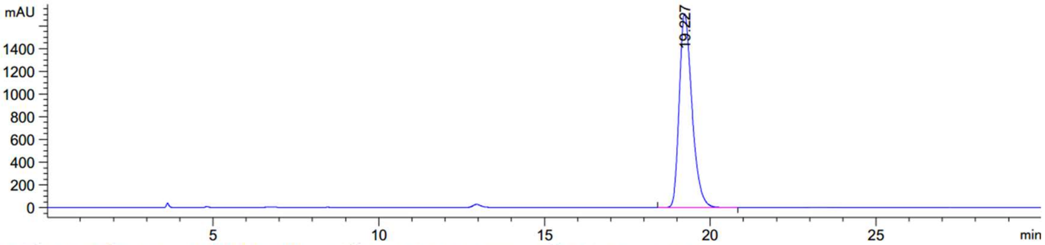

Signal 1: DAD1 A, Sig=254,4 Ref=360,100

Peak #	RetTime [min]	Type	Width [min]	Area [mAU*s]	Height [mAU]	Area %
1	19.227	BB	0.4094	4.55613e4	1706.92480	100.0000
Totals :				4.55613e4	1706.92480	

Figure S17. HPLC Spectra for dr Determination. **(*R*,2*R*)-6b.**The dr was determined by LC-MS with binary pump, photodiode array detector (DAD), using Eclipse XDB-C18 column (250 × 4.6 mm, 5 μm) (CH3CN/H2O = 65:35, flow rate 1.0 mL/min, λ = 254 nm), tmajor = 19.227 min, tminor = not found, dr > 99:1.

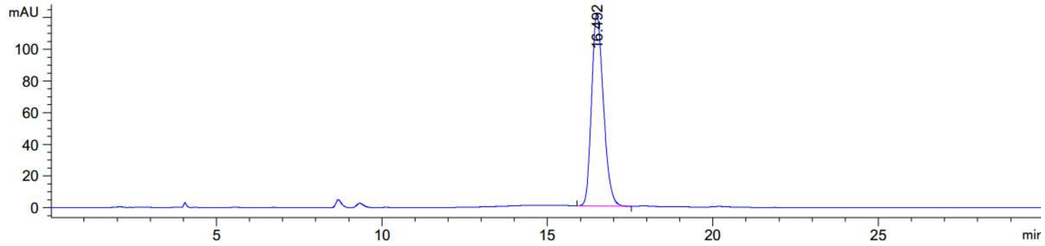

Signal 1: DAD1 A, Sig=254,4 Ref=360,100

Peak #	RetTime [min]	Type	Width [min]	Area [mAU*s]	Height [mAU]	Area %
1	16.492	BB	0.3817	3012.85474	121.35284	100.0000

Totals : 3012.85474 121.35284

Figure S18. HPLC Spectra for dr Determination. **(*R*,2*R*)-6c.**The dr was determined by LC-MS with binary pump, photodiode array detector (DAD), using Eclipse XDB-C18 column (250 × 4.6 mm, 5 μm) (CH3CN/H2O = 65:35, flow rate 1.0 mL/min, λ = 254 nm), tmajor = 16.492 min, tminor = not found, dr > 99:1

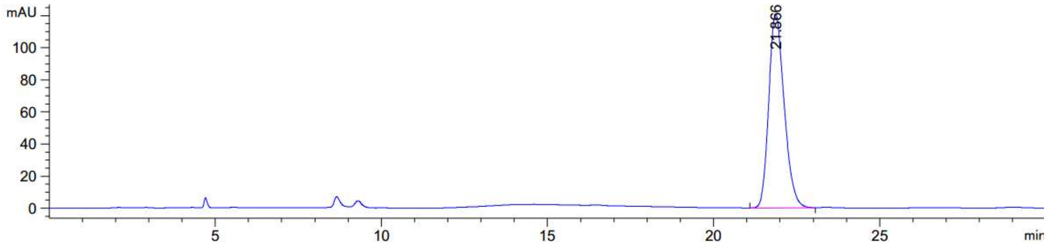

Signal 1: DAD1 A, Sig=254,4 Ref=360,100

Peak #	RetTime [min]	Type	Width [min]	Area [mAU*s]	Height [mAU]	Area %
1	21.866	BB	0.4960	3895.11133	120.85636	100.0000

Totals : 3895.11133 120.85636

Figure S19. HPLC Spectra for dr Determination. **(*R*,2*R*)-6d.**The dr was determined by LC-MS with binary pump, photodiode array detector (DAD), using Eclipse XDB-C18 column (250 × 4.6 mm, 5 μm) (CH3CN/H2O = 65:35, flow rate 1.0 mL/min, λ = 254 nm), tmajor = 21.866 min, tminor = not found, dr > 99:1.

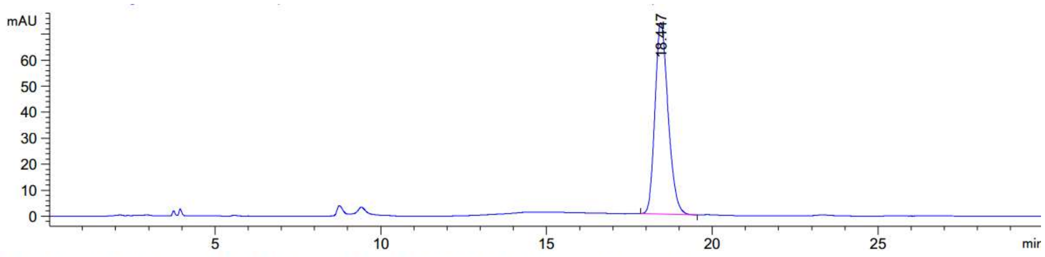

Signal 1: DAD1 A, Sig=254,4 Ref=360,100

Peak #	RetTime [min]	Type	Width [min]	Area [mAU*s]	Height [mAU]	Area %
1	18.447	BB	0.4252	2024.72644	73.54380	100.0000
Totals :				2024.72644	73.54380	

Figure S20. HPLC Spectra for dr Determination. **(*R*,2*R*)-6e.**The dr was determined by LC-MS with binary pump, photodiode array detector (DAD), using Eclipse XDB-C18 column (250 × 4.6 mm, 5 μm) ($CH3CN/H2O$ = 65:35, flow rate 1.0 mL/min, λ = 254 nm), tmajor = 18.447 min, tminor = not found, dr > 99:1.

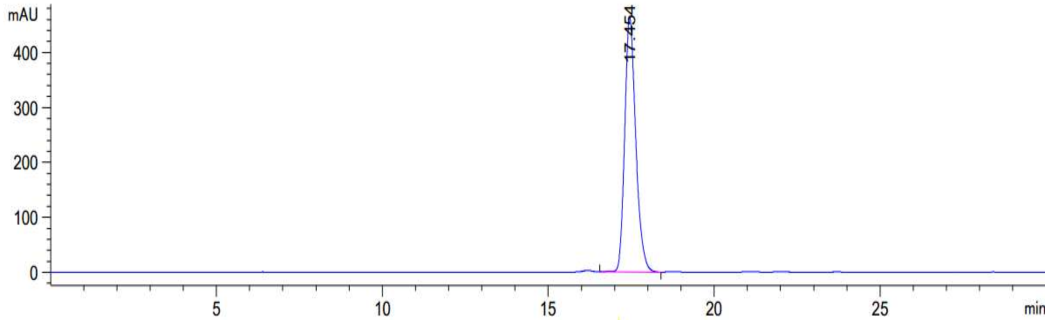

Signal 1: DAD1 A, Sig=254,4 Ref=360,100

Peak #	RetTime [min]	Type	Width [min]	Area [mAU*s]	Height [mAU]	Area %
1	17.454	BB	0.3543	1.07674e4	464.74875	100.0000
Totals :				1.07674e4	464.74875	

Signal 1: DAD1 A, Sig=254,4 Ref=360,100

Peak #	RetTime [min]	Type	Width [min]	Area [mAU*s]	H [
1	17.454	BB	0.3543	1.07674e4	46
Totals :				1.07674e4	46

Figure S21. HPLC Spectra for dr Determination. **(*R*,2*R*)-6f.**The dr was determined by LC-MS with binary pump, photodiode array detector (DAD), using Eclipse XDB-C18 column (250 × 4.6 mm, 5 μm) ($CH3CN/H2O$ = 65:35, flow rate 1.0 mL/min, λ = 254 nm), tmajor = 17.454 min, tminor = not found, dr > 99:1.

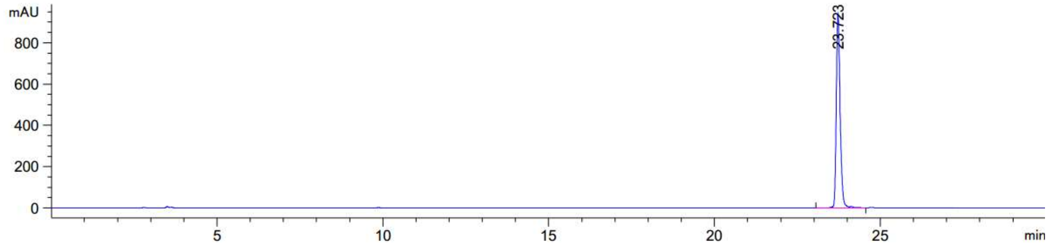

Signal 1: DAD1 A, Sig=254,4 Ref=360,100

Peak #	RetTime [min]	Type	Width [min]	Area [mAU*s]	Height [mAU]	Area %
1	23.723	BV R	0.1217	7602.73975	940.68359	100.0000
Totals :				7602.73975	940.68359	

Figure S22. HPLC Spectra for dr Determination. **(*R*,2*R*)-6g.**The dr was determined by LC-MS with binary pump, photodiode array detector (DAD), using Eclipse XDB-C18 column (250 × 4.6 mm, 5 μm) (CH3CN/H2O = 65:35, flow rate 1.0 mL/min, λ = 254 nm), tmajor = 23.723 min, tminor = not found, dr > 99:1.

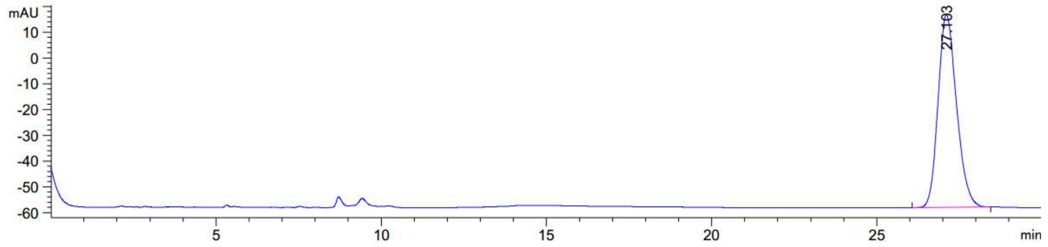

Signal 1: DAD1 A, Sig=254,4 Ref=360,100

Peak #	RetTime [min]	Type	Width [min]	Area [mAU*s]	Height [mAU]	Area %
1	27.103	BB	0.5965	2902.74463	74.83443	100.0000
Totals :				2902.74463	74.83443	

Figure S23. HPLC Spectra for dr Determination. **(*R*,2*R*)-6h.**The dr was determined by LC-MS with binary pump, photodiode array detector (DAD), using Eclipse XDB-C18 column (250 × 4.6 mm, 5 μm) (CH3CN/H2O = 65:35, flow rate 1.0 mL/min, λ = 254 nm), tmajor = 19.227 min, tminor = not found, dr > 99:1.

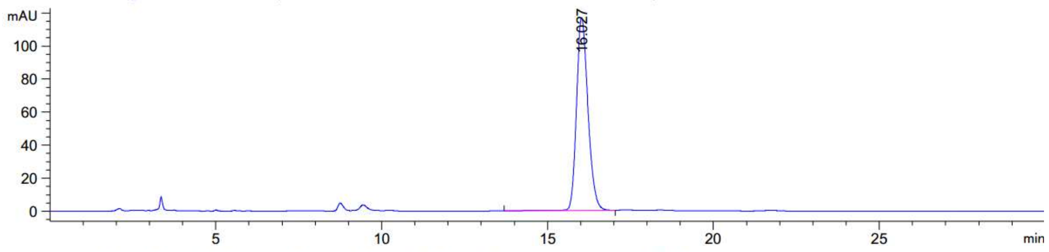

Signal 1: DAD1 A, Sig=254,4 Ref=360,100

Peak #	RetTime [min]	Type	Width [min]	Area [mAU*s]	Height [mAU]	Area %
1	16.027	BB	0.3683	2803.96143	116.68654	100.0000
Totals :				2803.96143	116.68654	

Figure S24. HPLC Spectra for dr Determination. **(*R*,2*R*)-6i.**The dr was determined by LC-MS with binary pump, photodiode array detector (DAD), using Eclipse XDB-C18 column (250 × 4.6 mm, 5 μm) (CH3CN/H2O = 65:35, flow rate 1.0 mL/min, λ = 254 nm), tmajor = 16.027 min, tminor = not found, dr > 99:1.

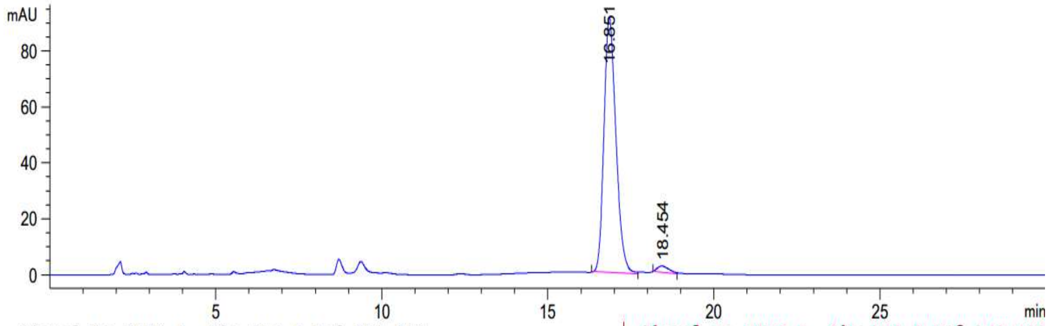

Signal 1: DAD1 A, Sig=254,4 Ref=360,100

Peak #	RetTime [min]	Type	Width [min]	Area [mAU*s]	Height [mAU]	Area %
1	16.851	MM R	0.4161	2277.50513	91.23013	97.6470
2	18.454	MM R	0.4143	54.88123	2.20771	2.3530
Totals :				2332.38635	93.43784	

Signal 1: DAD1 A, Sig=254,4 Ref=360,100

Peak #	RetTime [min]	Type	Width [min]	Area [mAU*s]
1	16.851	MM R	0.4161	2277.50513
2	18.454	MM R	0.4143	54.88123
Totals :				2332.38635

Figure S25. HPLC Spectra for dr Determination. **(*R*,2*R*)-6j.** The dr was determined by LC-MS with binary pump, photodiode array detector (DAD), using Eclipse XDB-C18 column (250 × 4.6 mm, 5 μm) (CH3CN/H2O = 65:35, flow rate 1.0 mL/min, λ = 254 nm), tmajor = 16.851 min, tminor = 18.454, dr = 98:2.

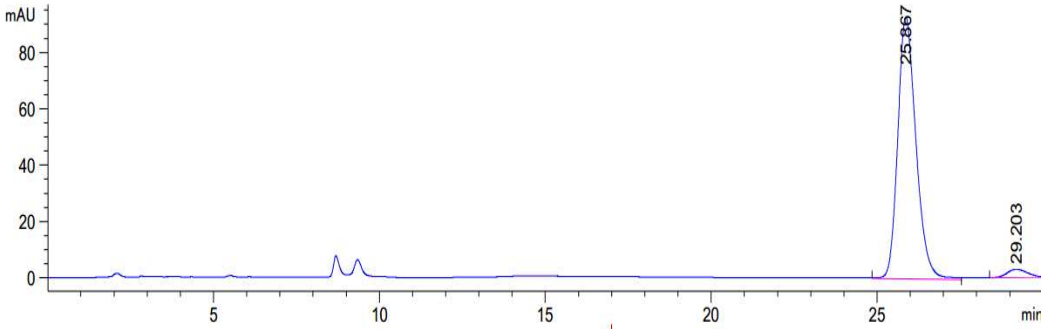

Signal 1: DAD1 A, Sig=254,4 Ref=360,100

Peak #	RetTime [min]	Type	Width [min]	Area [mAU*s]	Height [mAU]	Area %
1	25.867	MM R	0.6347	3534.49023	92.80738	96.7458
2	29.203	BBA	0.6299	118.88742	2.87795	3.2542
Totals :				3653.37766	95.68533	

Signal 1: DAD1 A, Sig=254,4 Ref=360,100

Peak #	RetTime [min]	Type	Width [min]	Area [mAU*s]	H [
1	25.867	MM R	0.6347	3534.49023	9
2	29.203	BBA	0.6299	118.88742	
Totals :				3653.37766	9

Figure S26. HPLC Spectra for dr Determination. **(*R*,2*R*)-6k.** The dr was determined by LC-MS with binary pump, photodiode array detector (DAD), using Eclipse XDB-C18 column (250 × 4.6 mm, 5 μm) (CH3CN/H2O = 65:35, flow rate 1.0 mL/min, λ = 254 nm), tmajor = 25.867 min, tminor = 29.203, dr = 97:3.

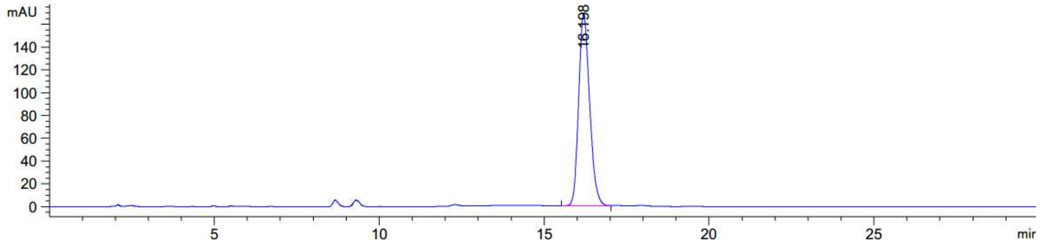

Signal 1: DAD1 A, Sig=254,4 Ref=360,100

Peak #	RetTime [min]	Type	Width [min]	Area [mAU*s]	Height [mAU]	Area %
1	16.198	BB	0.3573	3888.09253	168.44102	100.0000
Totals :				3888.09253	168.44102	

Figure S27. HPLC Spectra for dr Determination. **(*R*,2*R*)-6l.** The dr was determined by LC-MS with binary pump, photodiode array detector (DAD), using Eclipse XDB-C18 column (250 × 4.6 mm, 5 μm) (CH3CN/H2O = 65:35, flow rate 1.0 mL/min, λ = 254 nm), tmajor = 16.198 min, tminor = not found, dr > 99:1.

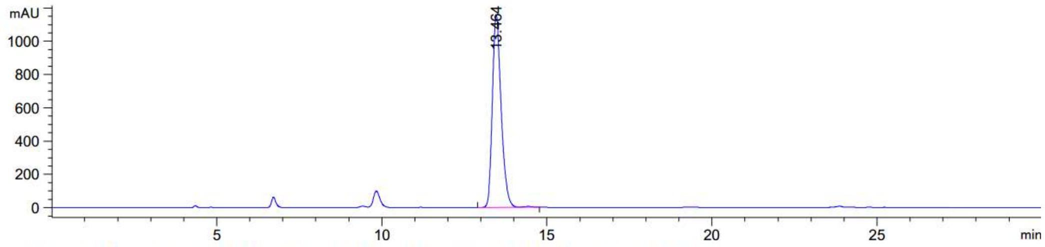

Signal 1: DAD1 A, Sig=254,4 Ref=360,100

Peak #	RetTime [min]	Type	Width [min]	Area [mAU*s]	Height [mAU]	Area %
1	13.464	BV R	0.2817	2.15247e4	1158.18042	100.0000
Totals :				2.15247e4	1158.18042	

Figure S28. HPLC Spectra for dr Determination. **(*R*,2*R*)-6m.** The dr was determined by LC-MS with binary pump, photodiode array detector (DAD), using Eclipse XDB-C18 column (250 × 4.6 mm, 5 μm) (CH3CN/H2O = 65:35, flow rate 1.0 mL/min, λ = 254 nm), tmajor = 13.464 min, tminor = not found, dr > 99:1.

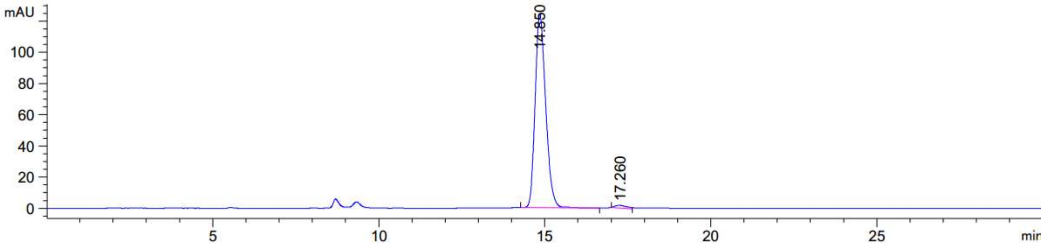

Signal 1: DAD1 A, Sig=254,4 Ref=360,100

Peak #	RetTime [min]	Type	Width [min]	Area [mAU*s]	Height [mAU]	Area %
1	14.850	BB	0.3401	2751.26807	124.34421	98.4359
2	17.260	MM R	0.4245	43.71647	1.71646	1.5641
Totals :				2794.98454	126.06067	

Figure S29. HPLC Spectra for dr Determination. **(*R*,2*R*)-6n.** The dr was determined by LC-MS with binary pump, photodiode array detector (DAD), using Eclipse XDB-C18 column (250 × 4.6 mm, 5 μm) (CH3CN/H2O = 65:35, flow rate 1.0 mL/min, λ = 254 nm), tmajor = 14.850 min, tminor = 17.260, dr = 98:2.

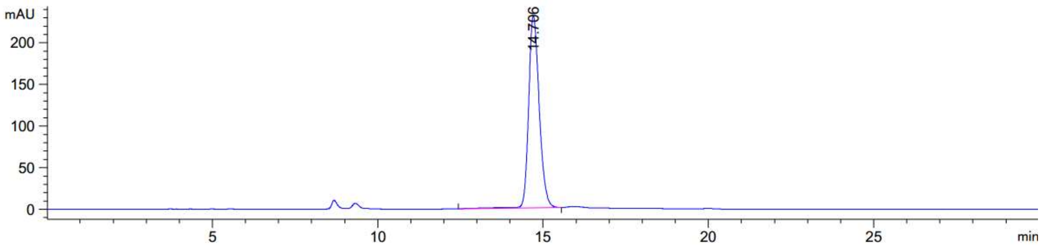

Signal 1: DAD1 A, Sig=254,4 Ref=360,100

Peak #	RetTime [min]	Type	Width [min]	Area [mAU*s]	Height [mAU]	Area %
1	14.706	BB	0.3415	5131.90820	230.68553	100.0000
Totals :				5131.90820	230.68553	

Figure S30. HPLC Spectra for dr Determination. **(*R*,2*R*)-6o.** The dr was determined by LC-MS with binary pump, photodiode array detector (DAD), using Eclipse XDB-C18 column (250 × 4.6 mm, 5 μm) (CH3CN/H2O = 65:35, flow rate 1.0 mL/min, λ = 254 nm), tmajor = 14.706 min, tminor = not found, dr > 99:1.

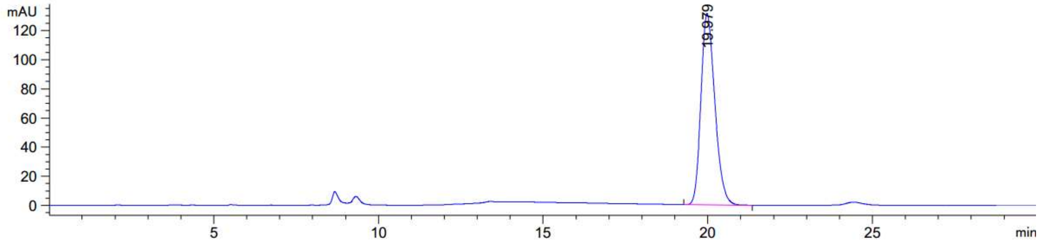

Signal 1: DAD1 A, Sig=254,4 Ref=360,100

Peak #	RetTime [min]	Type	Width [min]	Area [mAU*s]	Height [mAU]	Area %
1	19.979	BB	0.4530	3861.24878	131.33554	100.0000
Totals :				3861.24878	131.33554	

Figure S31. HPLC Spectra for dr Determination. **(*R*,2*R*)-6p.** The dr was determined by LC-MS with binary pump, photodiode array detector (DAD), using Eclipse XDB-C18 column (250 × 4.6 mm, 5 μm) (CH3CN/H2O = 65:35, flow rate 1.0 mL/min, λ = 254 nm), tmajor = 19.979 min, tminor = not found, dr > 99:1.

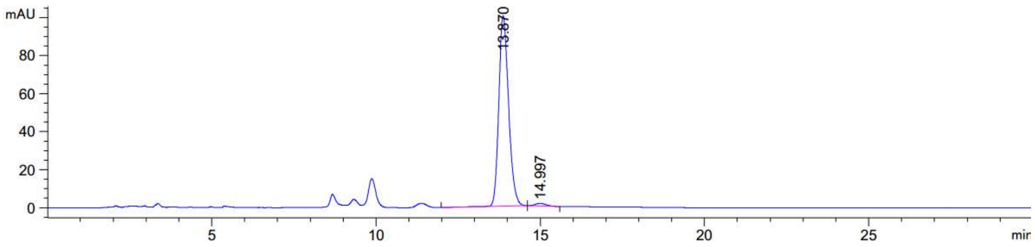

Signal 1: DAD1 A, Sig=254,4 Ref=360,100

Peak #	RetTime [min]	Type	Width [min]	Area [mAU*s]	Height [mAU]	Area %
1	13.870	BB	0.3176	2074.31128	100.17336	98.4997
2	14.997	BB	0.3420	31.59428	1.41756	1.5003

Totals : 2105.90556 101.59091

Figure S32. HPLC Spectra for dr Determination. **(*R*,2*R*)-6q.** The dr was determined by LC-MS with binary pump, photodiode array detector (DAD), using Eclipse XDB-C18 column (250 × 4.6 mm, 5 μm) (CH3CN/H2O = 65:35, flow rate 1.0 mL/min, λ = 254 nm), tmajor = 13.870 min, tminor = 14.997, dr = 98:2.

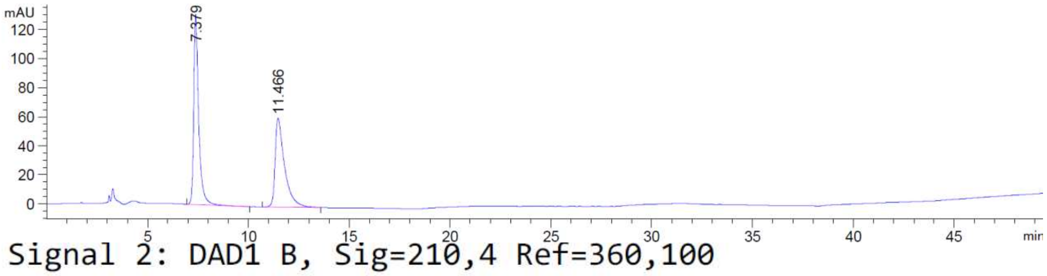

Signal 2: DAD1 B, Sig=210,4 Ref=360,100

Peak #	RetTime [min]	Type	Width [min]	Area [mAU*s]	Height [mAU]	Area %
1	7.379	BB	0.2493	2235.69507	130.86295	52.9964
2	11.466	BB	0.4662	1982.88416	61.17502	47.0036
Totals :				4218.57922	192.03796	

Figure S33. HPLC Spectra for ee Determination. ***(rac)*-phenylalanine**. The ee was determined by HPLC with an Astec CHIROBIOTIC™ T chiral HPLC column (4.6 mm × 25 cm, 5 μm) (MeOH/H2O = 90/10, λ = 210 nm, 1 mL/min). t_S = 7.379 min, t_R = 11.466.

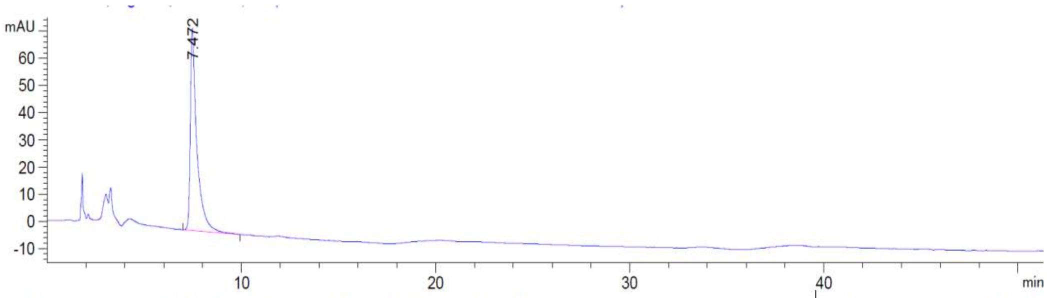

Signal 2: DAD1 B, Sig=210,4 Ref=360,100

Peak #	RetTime [min]	Type	Width [min]	Area [mAU*s]	Height [mAU]	Area %
1	7.472	BB	0.3288	1741.18774	74.45191	100.0000
Totals :				1741.18774	74.45191	

Signal 2: DAD1 [illegible]

Peak #	RetTime [min]	Typ[illegible]
1	7.472	BB
Totals :		

Figure S34: HPLC Spectra for ee Determination. ***(S)*-phenylalanine**. The ee was determined by HPLC with an Astec CHIROBIOTIC™ T chiral HPLC column (4.6 mm × 25 cm, 5 μm) (MeOH/H2O = 90/10, λ = 210 nm, 1 mL/min). t_S = 7.472 min, t_R = not found, ee > 99 %.

Figure S35: Nickel(II)-(*R*)-*N*-(2-benzoyl-4-chlorophenyl)-1-(3,4-dichlorobenzyl)-2-methylpyrrolidine-2-carboxamide/(*R*)-phenylalanine Schiff Base Complex 6a

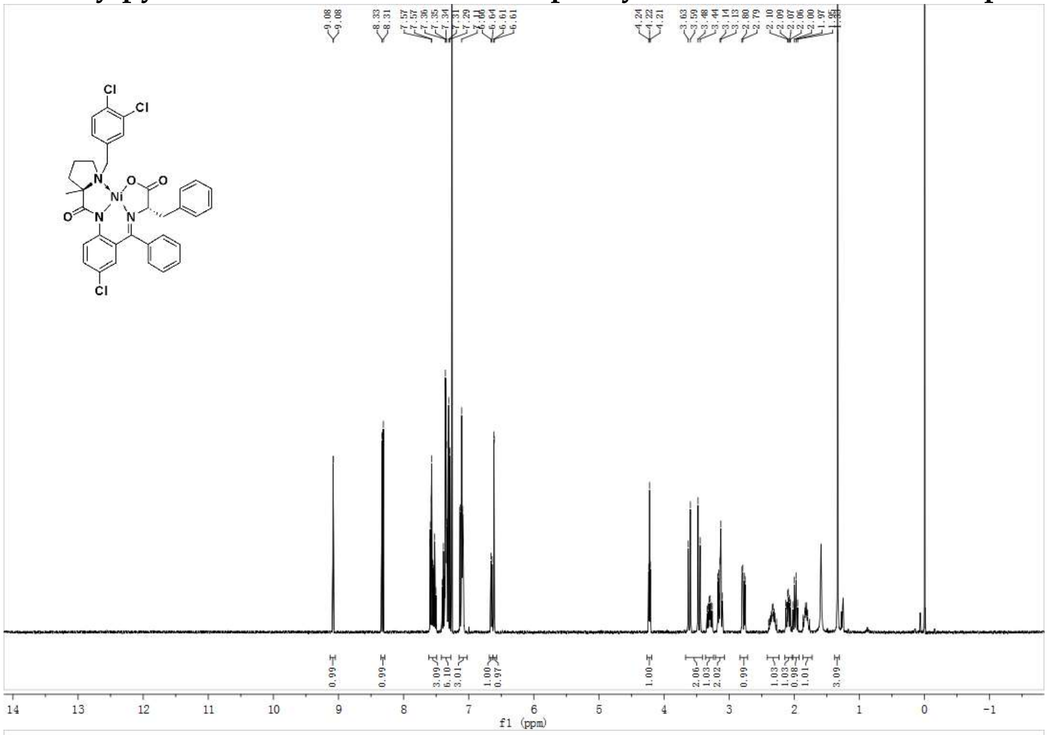

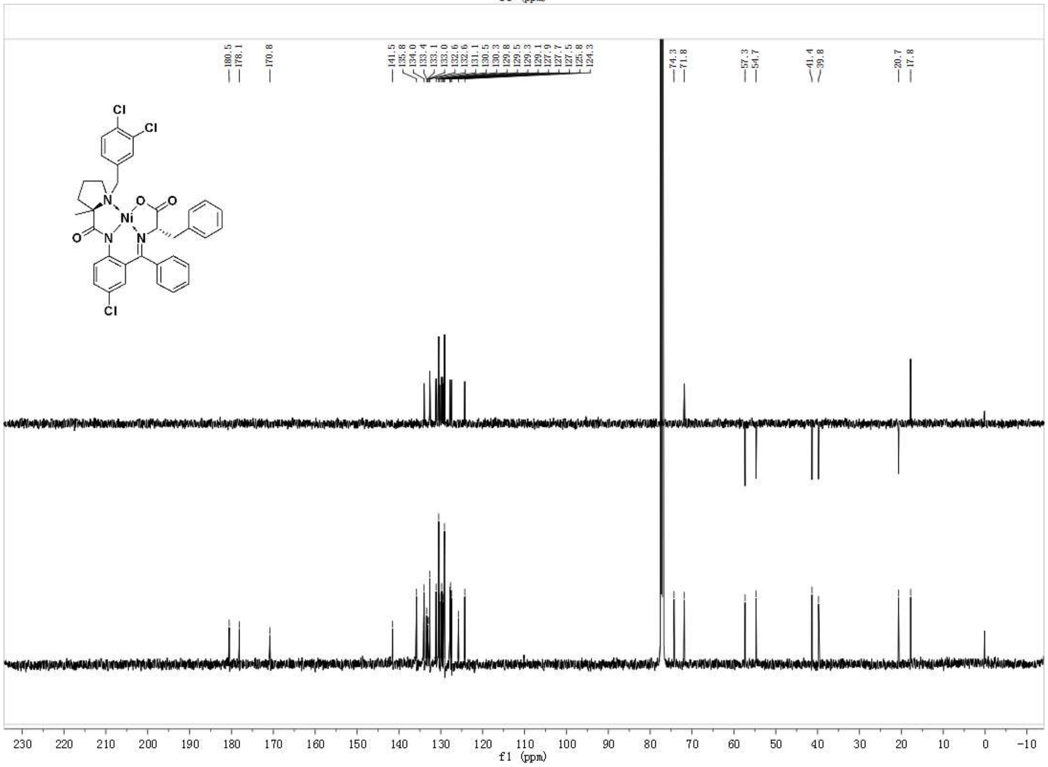

Figure S36: Nickel(II)-(*R*)-*N*-(2-benzoyl-4-chlorophenyl)-1-(3,4-dichlorobenzyl)-2-methylpyrrolidine-2-carboxamide/(*R*)-2-methoxyphenylalanine Schiff Base Complex 6b

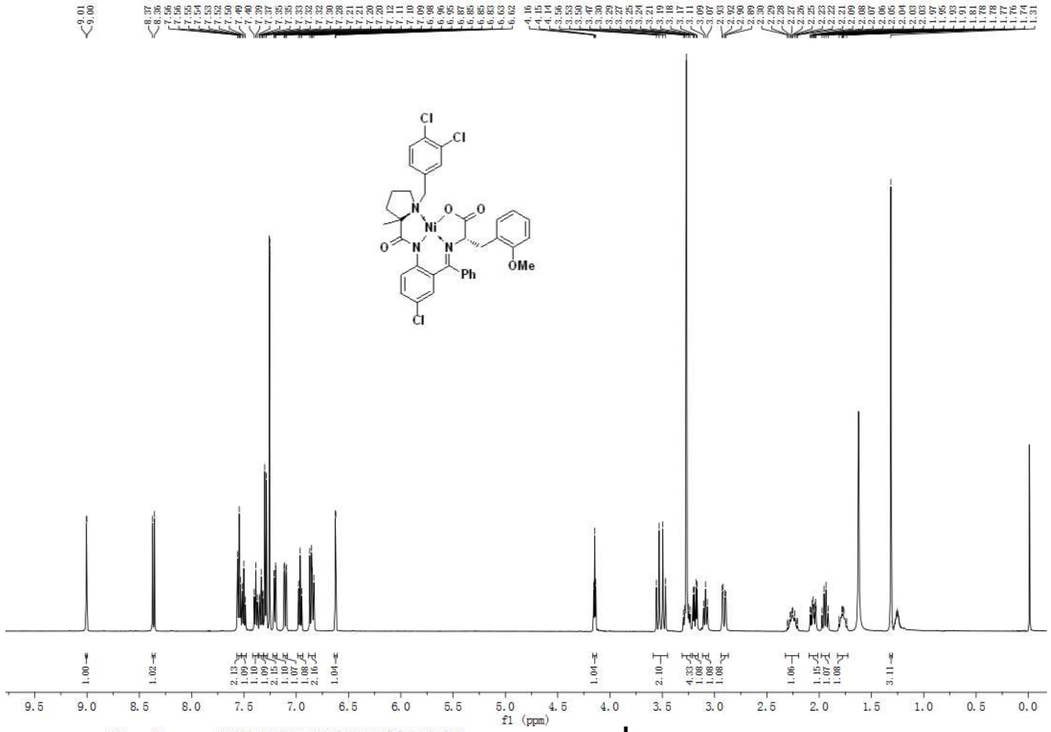

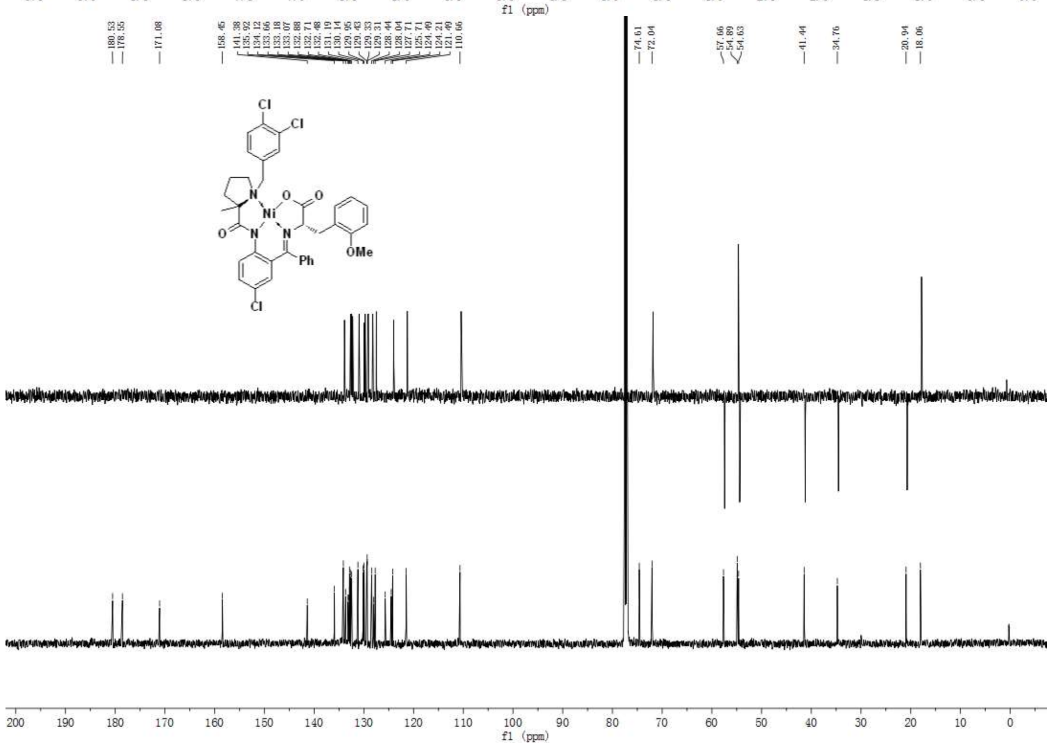

Figure S37: Nickel(II)-(*R*)-*N*-(2-benzoyl-4-chlorophenyl)-1-(3,4-dichlorobenzyl)-2-methylpyrrolidine-2-carboxamide/(*R*)-3-methoxyphenylalanine Schiff Base Complex 6c

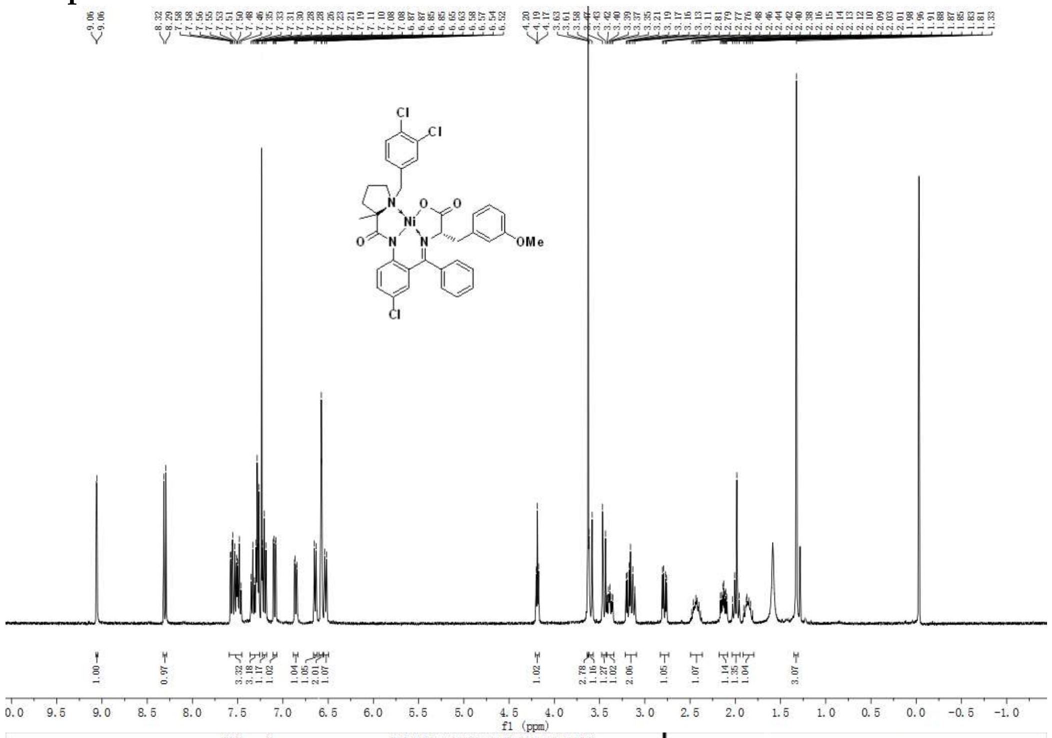

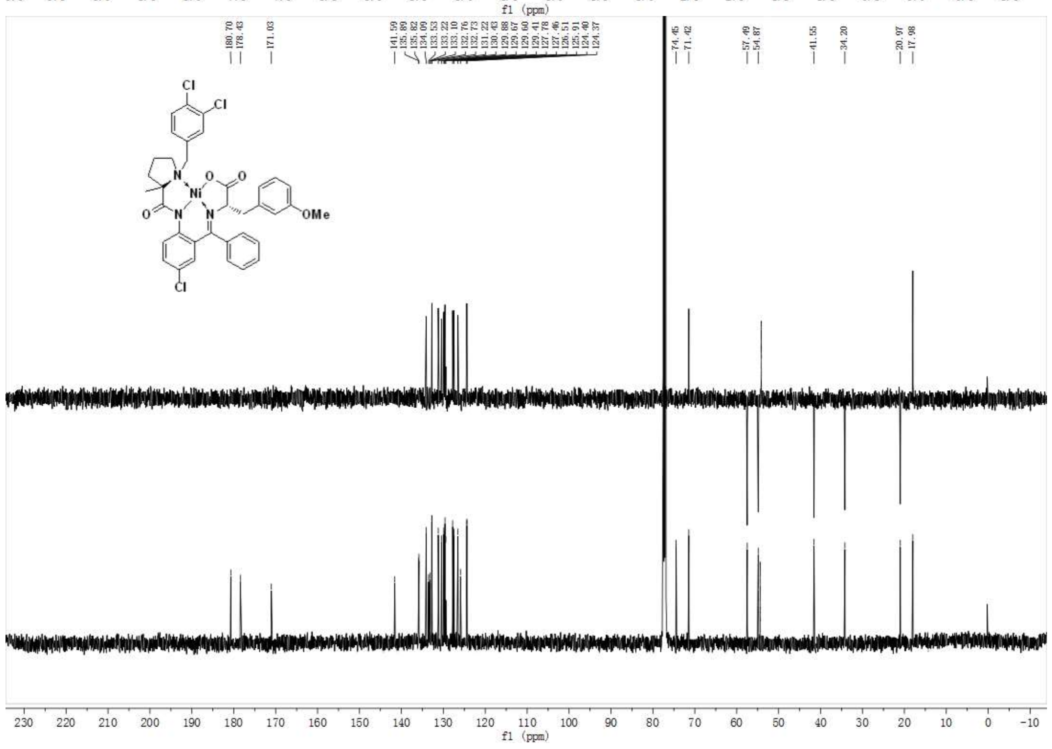

Figure S38: Nickel(II)-(*R*)-*N*-(2-benzoyl-4-chlorophenyl)-1-(3,4-dichlorobenzyl)-2-methylpyrrolidine-2-carboxamide/(*R*)-3-methylphenylalanine Schiff Base Complex 6d

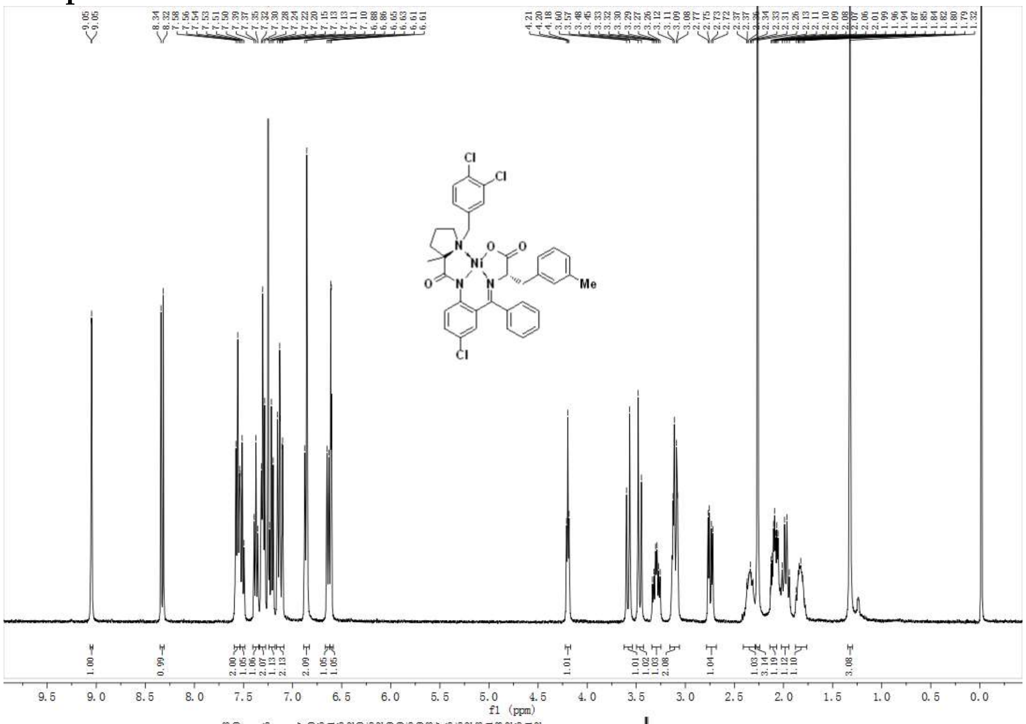

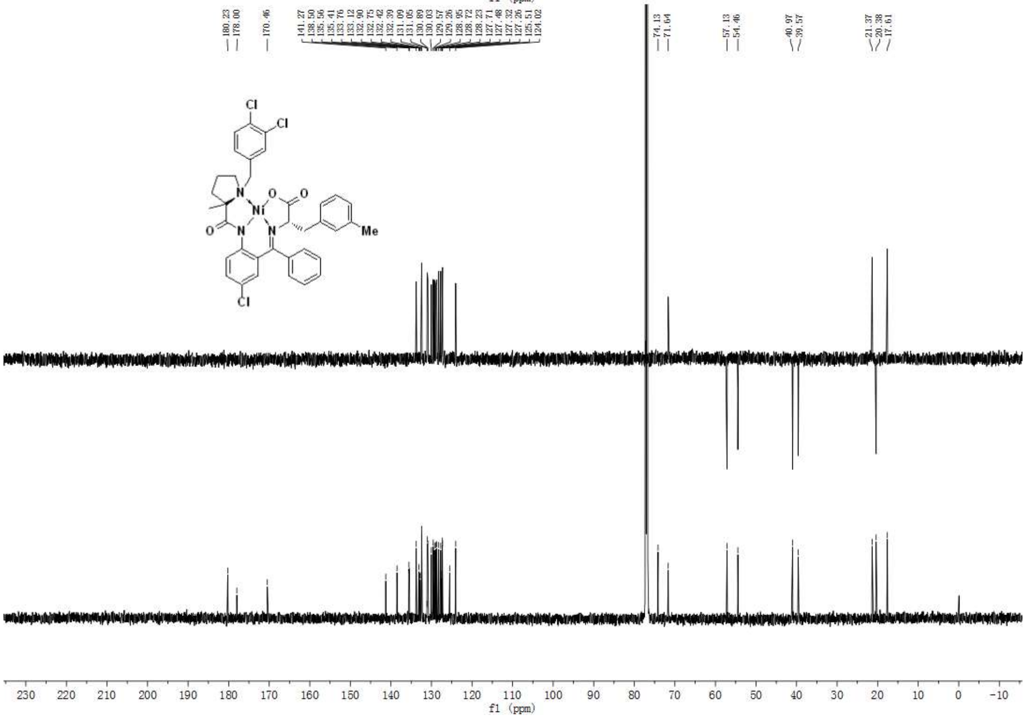

Figure S39: Nickel(II)-(*R*)-*N*-(2-benzoyl-4-chlorophenyl)-1-(3,4-dichlorobenzyl)-2-methylpyrrolidine-2-carboxamide/(*R*)-4-fluorophenylalanine Schiff Base Complex 6e

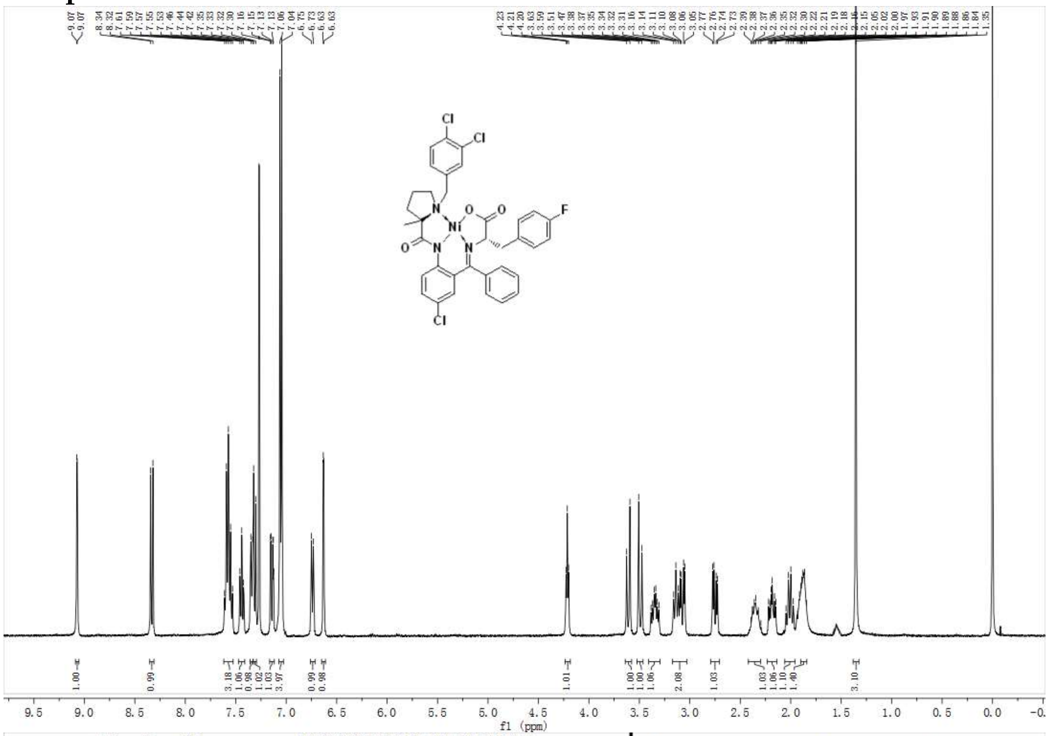

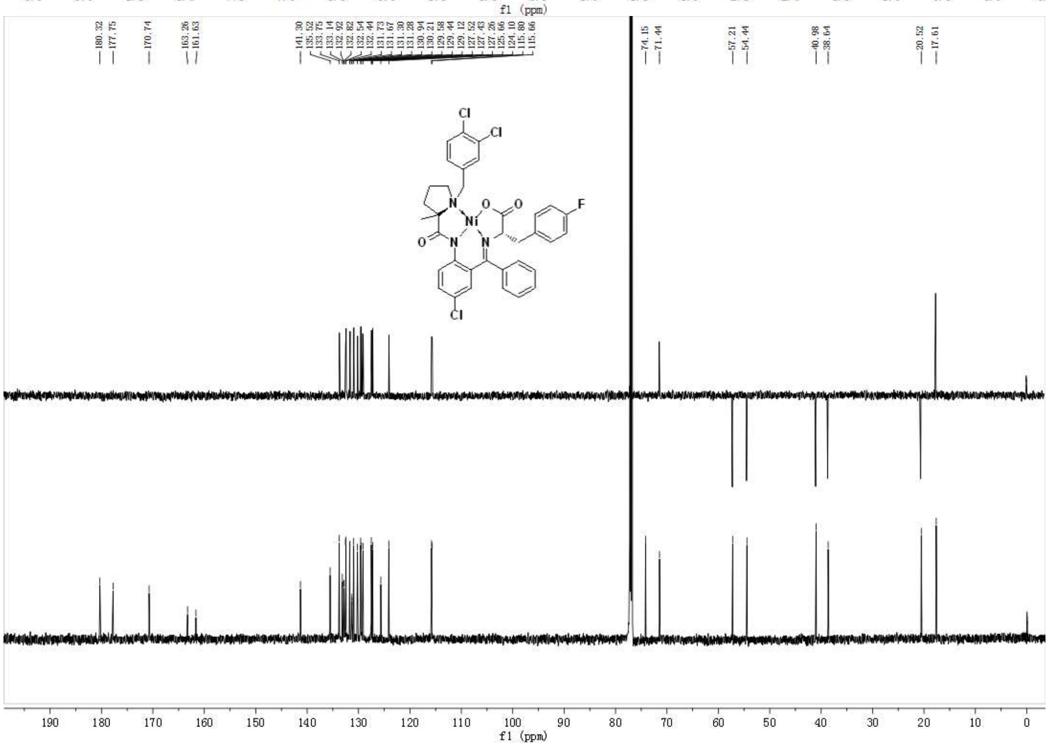

Figure S40: Nickel(II)-(*R*)-*N*-(2-benzoyl-4-chlorophenyl)-1-(3,4-dichlorobenzyl)-2-methylpyrrolidine-2-carboxamide/(*R*)-3,5-diiodotyrosine Schiff Base Complex 6f

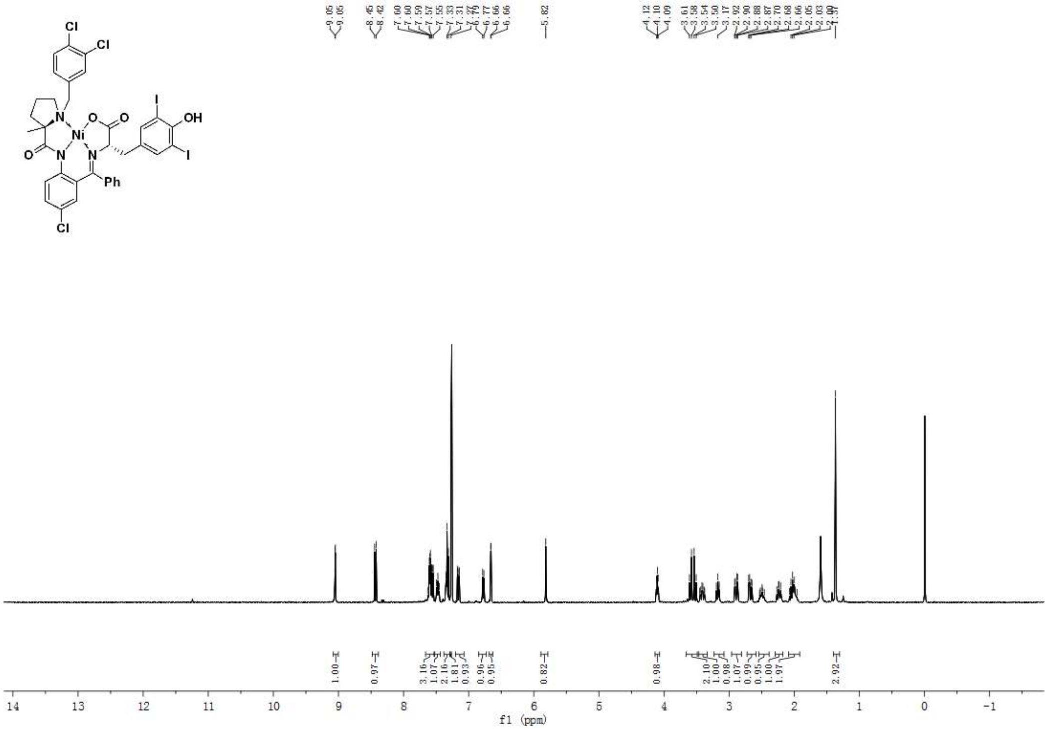

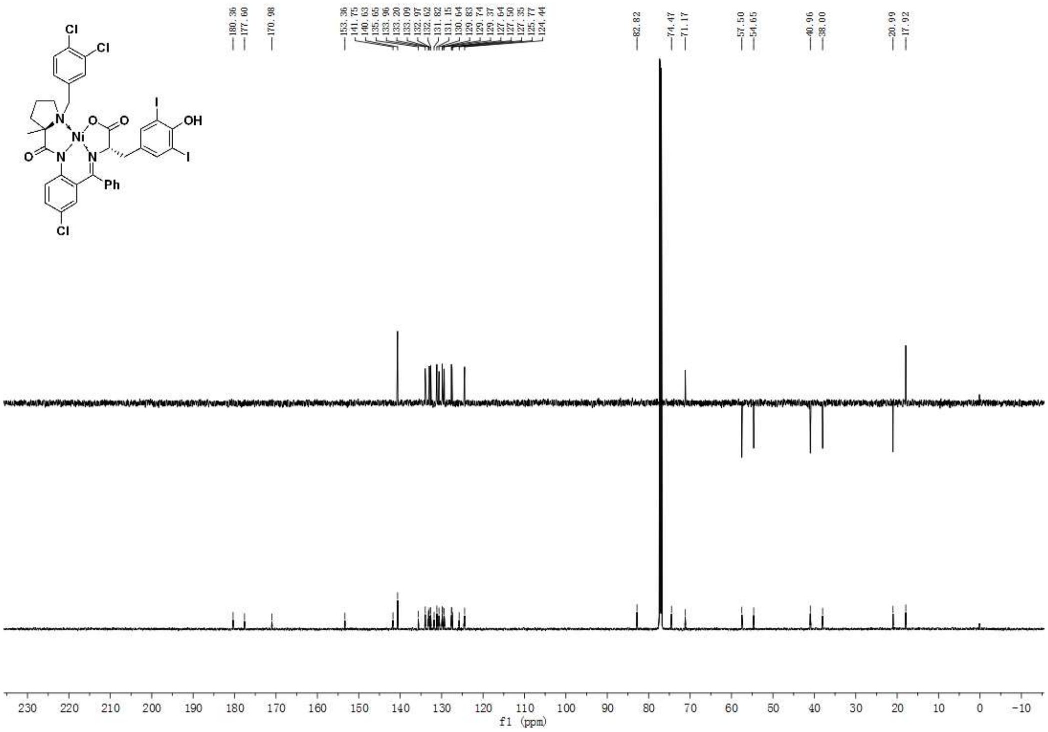

Figure S41: Nickel(II)-(*R*)-*N*-(2-benzoyl-4-chlorophenyl)-1-(3,4-dichlorobenzyl)-2-methylpyrrolidine-2-carboxamide/(*R*)-3-(1-naphthyl)alanine Schiff Base Complex 6g

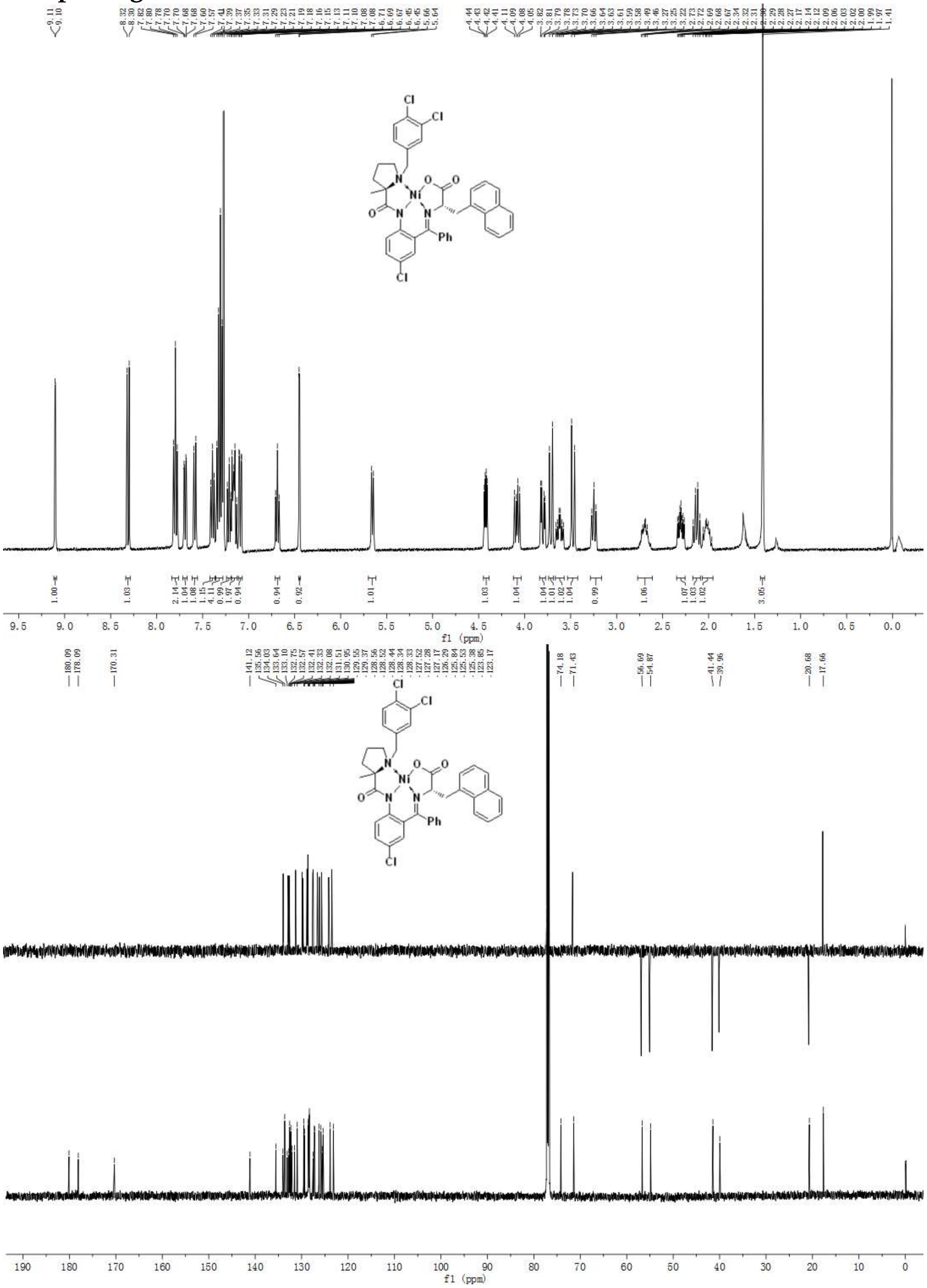

Figure S42: Nickel(II)-(*R*)-*N*-(2-benzoyl-4-chlorophenyl)-1-(3,4-dichlorobenzyl)-2-methylpyrrolidine-2-carboxamide/(*R*)-3-(3-benzothienyl)alanine Schiff Base Complex 6h

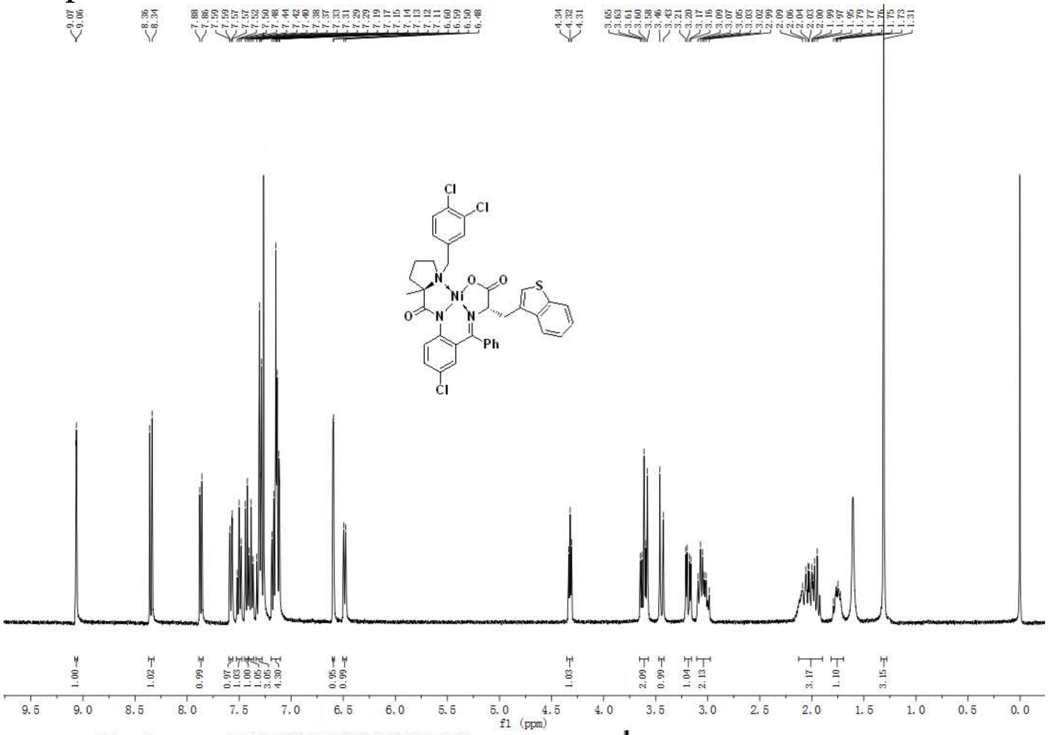

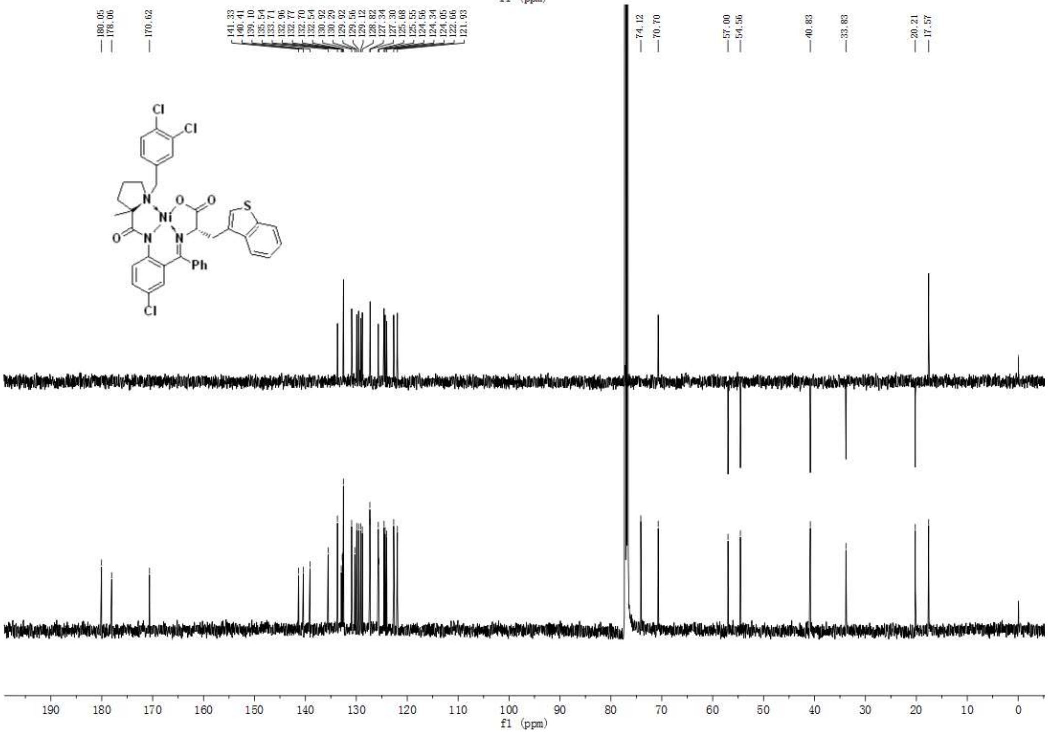

Figure S43: Nickel(II)-(*R*)-*N*-(2-benzoyl-4-chlorophenyl)-1-(3,4-dichlorobenzyl)-2-methylpyrrolidine-2-carboxamide/(*R*)-3-(3-thienyl)alanine Schiff Base Complex 6i

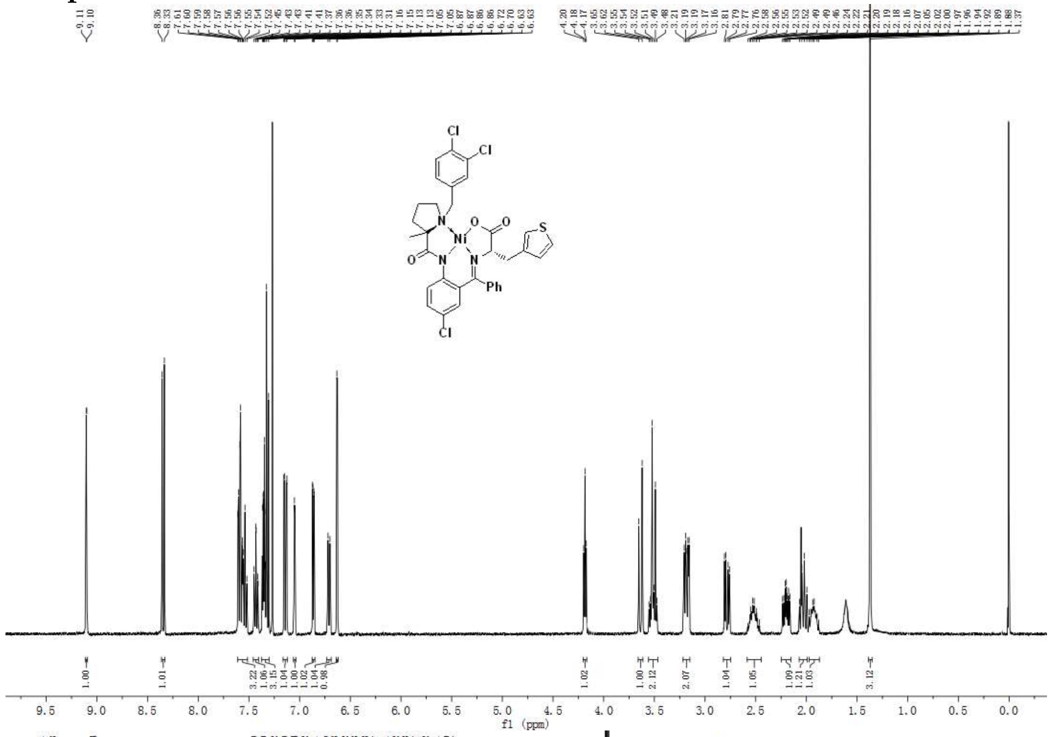

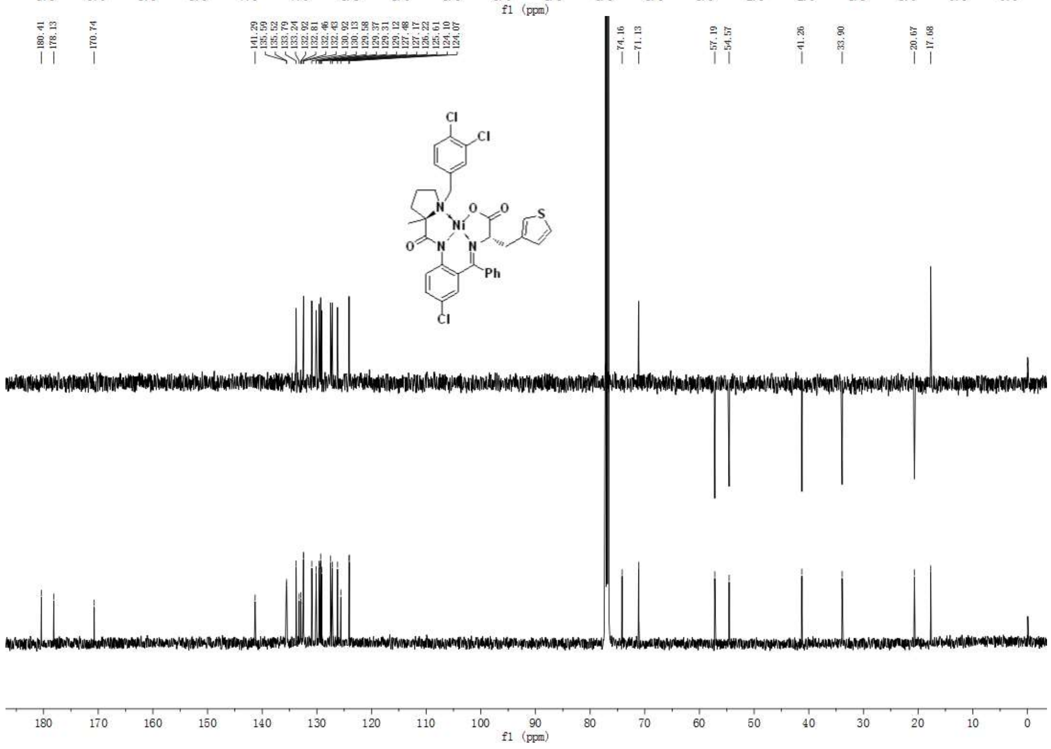

Figure S44: Nickel(II)-(*R*)-*N*-(2-benzoyl-4-chlorophenyl)-1-(3,4-dichlorobenzyl)-2-methylpyrrolidine-2-carboxamide/(*R*)-2-(3-methoxyphenyl)glycine Schiff Base Complex 6j

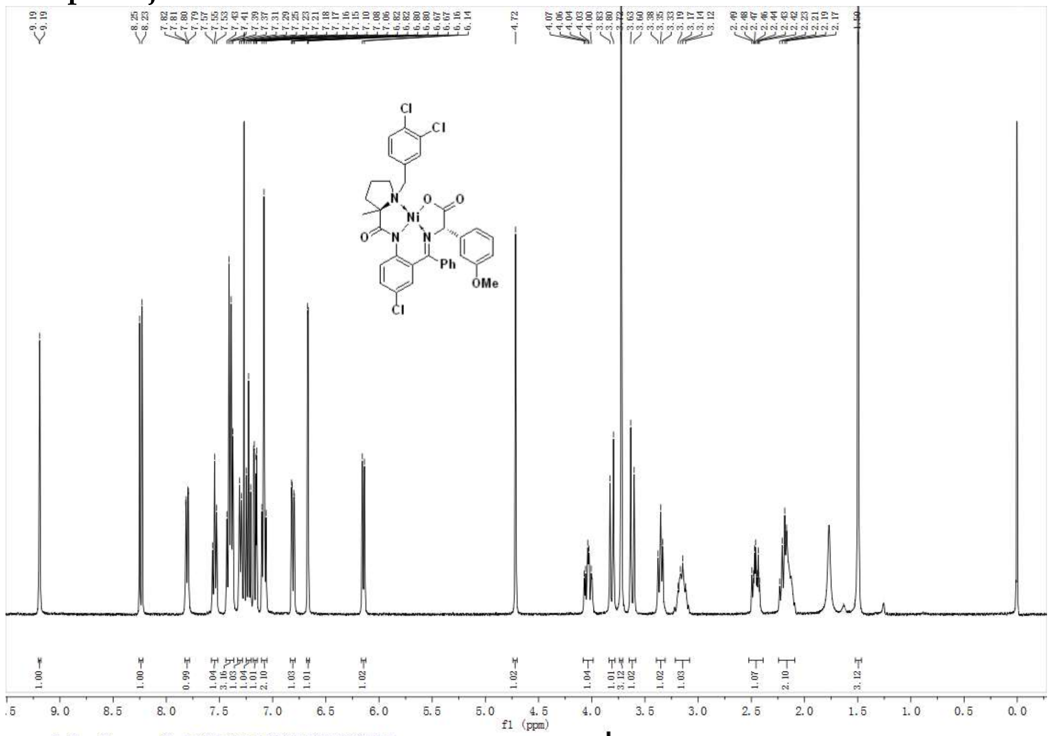

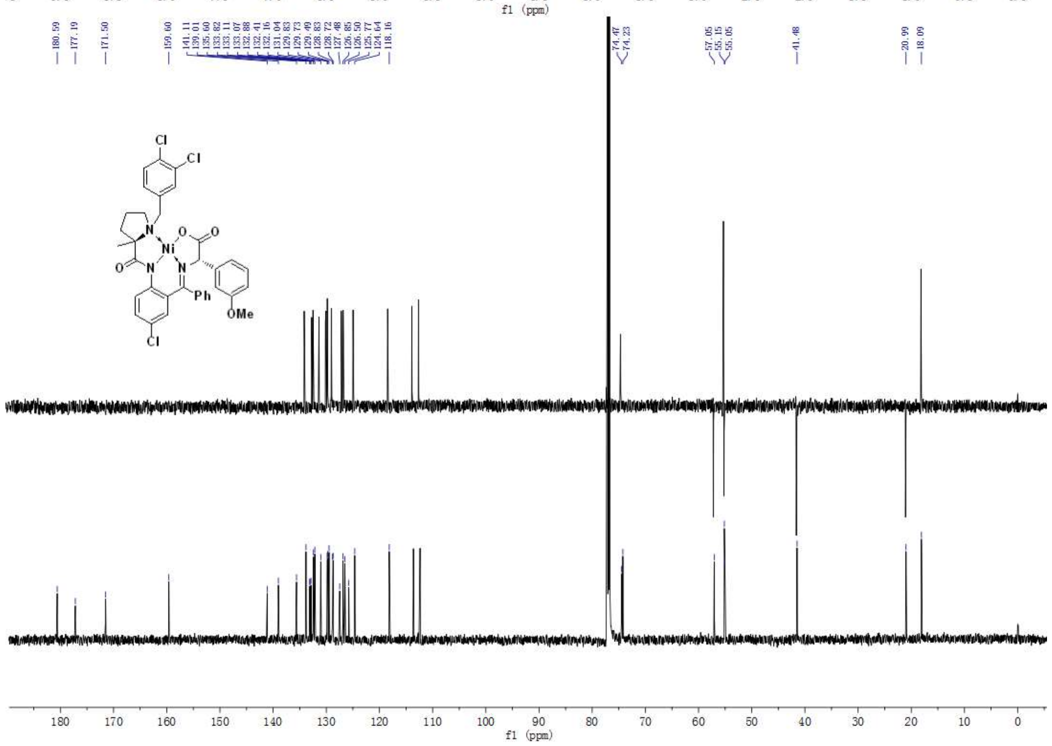

Figure S45: Nickel(II)-(*R*)-*N*-(2-benzoyl-4-chlorophenyl)-1-(3,4-dichlorobenzyl)-2-methylpyrrolidine-2-carboxamide/(*R*)-2-(3-bromophenyl)glycine Schiff Base Complex 6k

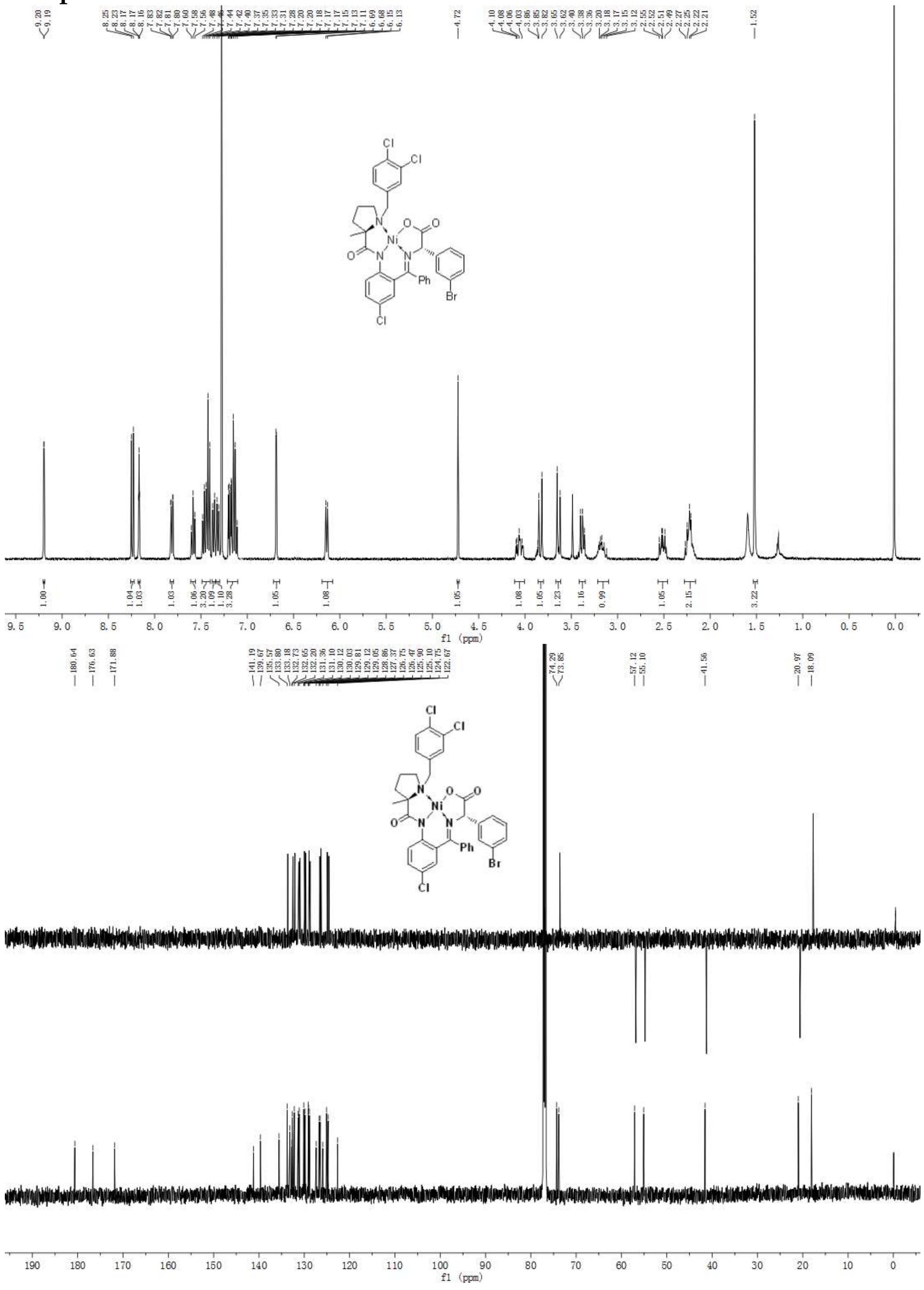

Figure S46: Nickel(II)-(*R*)-*N*-(2-benzoyl-4-chlorophenyl)-1-(3,4-dichlorobenzyl)-2-methylpyrrolidine-2-carboxamide/(*R*)-2-cyclobutylglycine Schiff Base Complex 6l

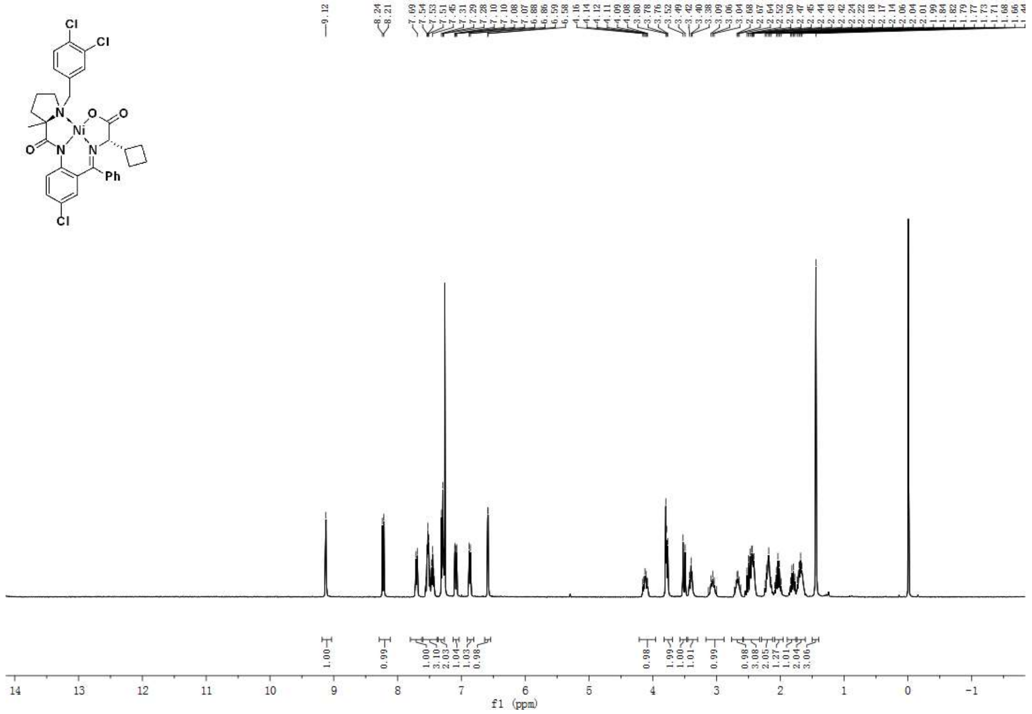

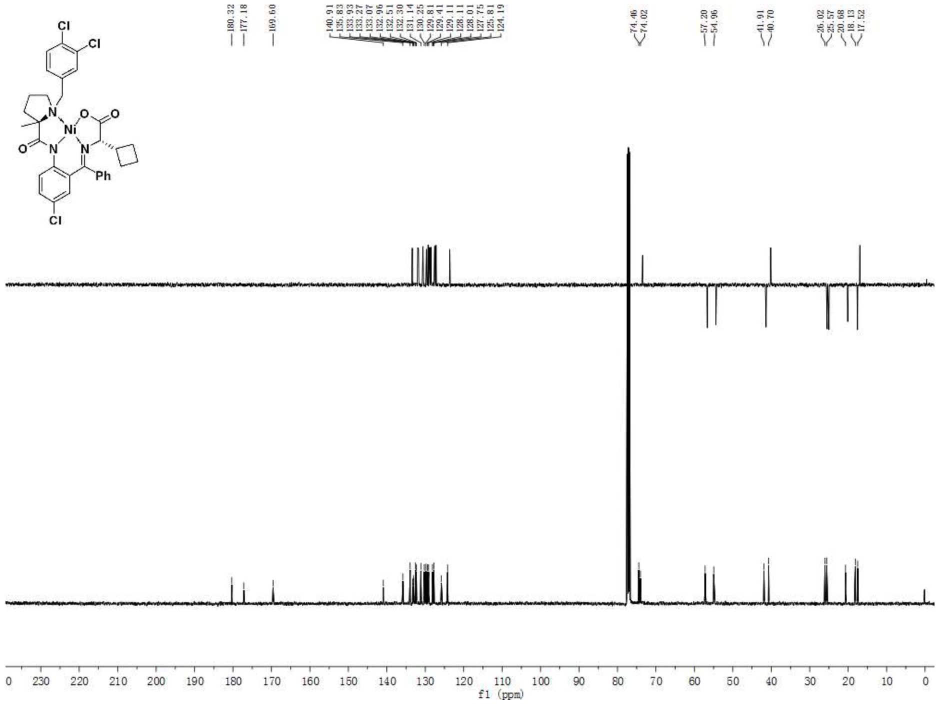

Figure S47: Nickel(II)-(*R*)-*N*-(2-benzoyl-4-chlorophenyl)-1-(3,4-dichlorobenzyl)-2-methylpyrrolidine-2-carboxamide/(*R*)-2-amino-4,4,4-trifluorobutyric acid Schiff Base Complex 6m

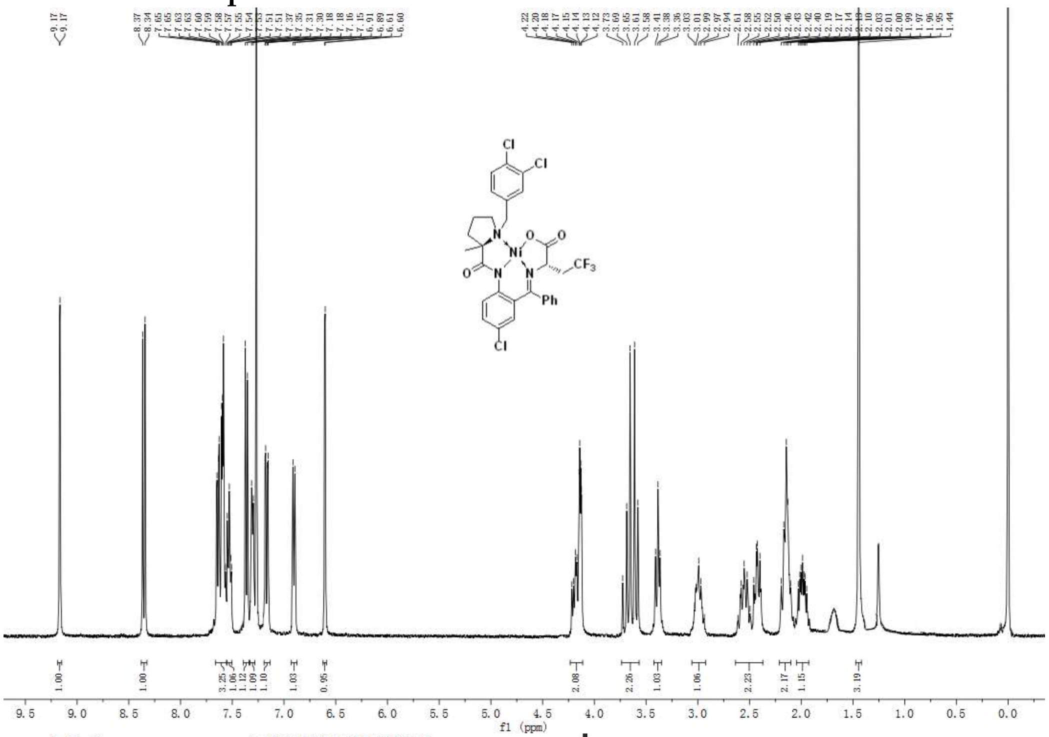

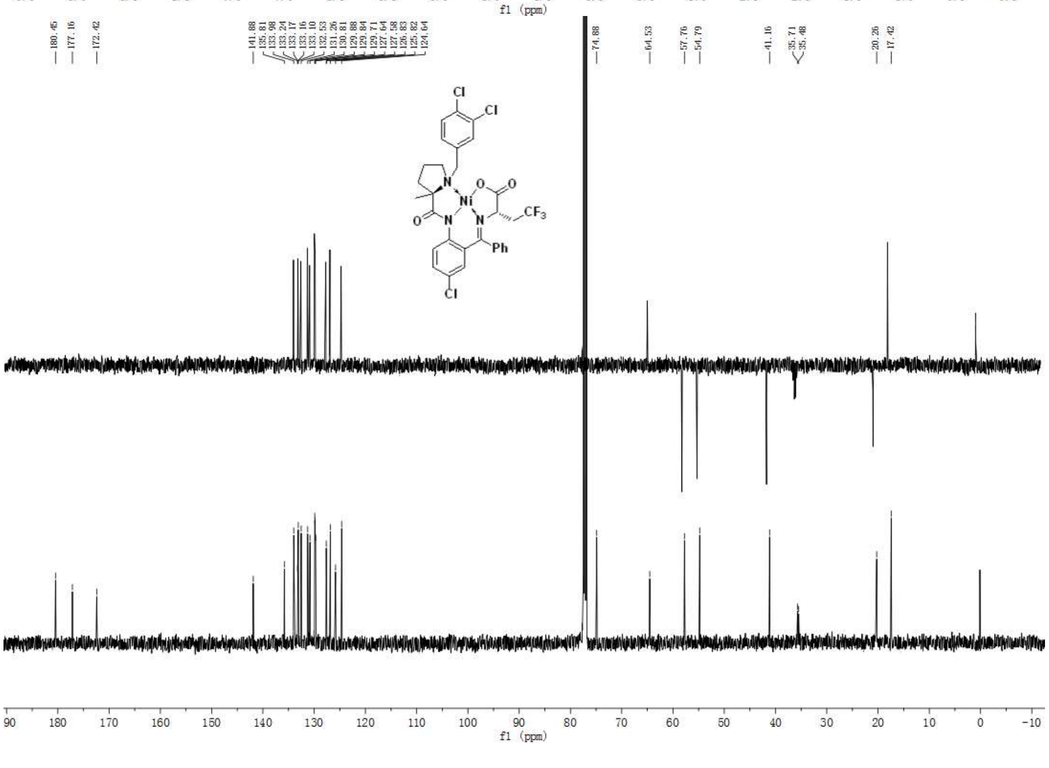

Figure S48: Nickel(II)-(*R*)-*N*-(2-benzoyl-4-chlorophenyl)-1-(3,4-dichlorobenzyl)-2-methylpyrrolidine-2-carboxamide/(*R*)-norvaline Schiff Base Complex 6n

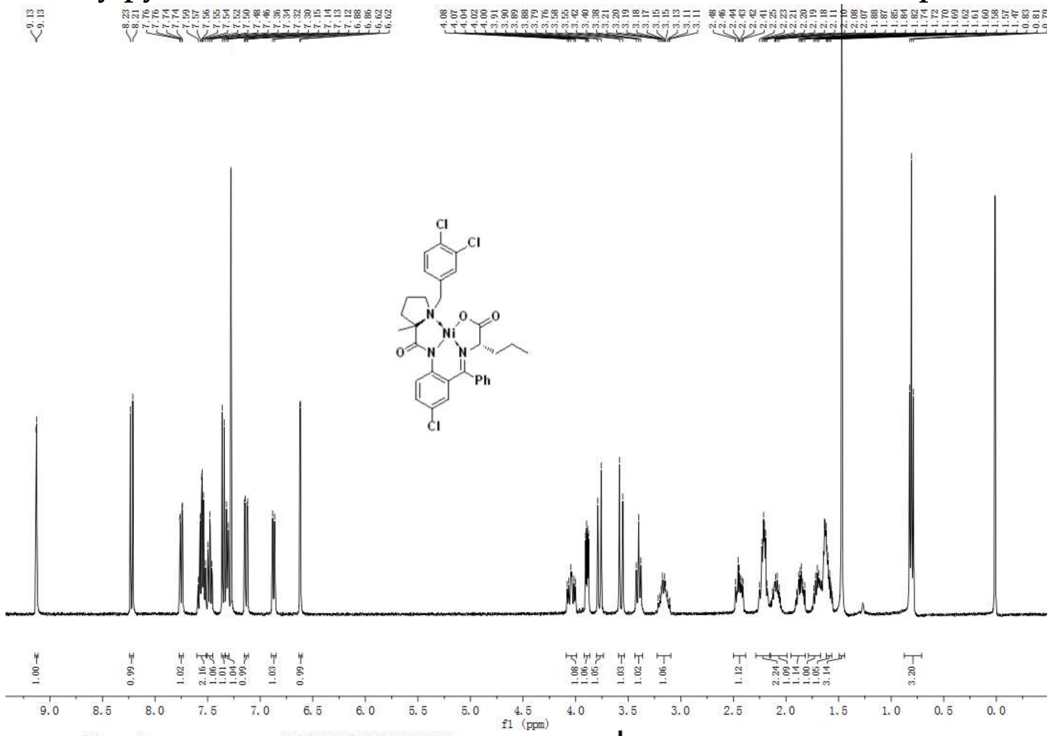

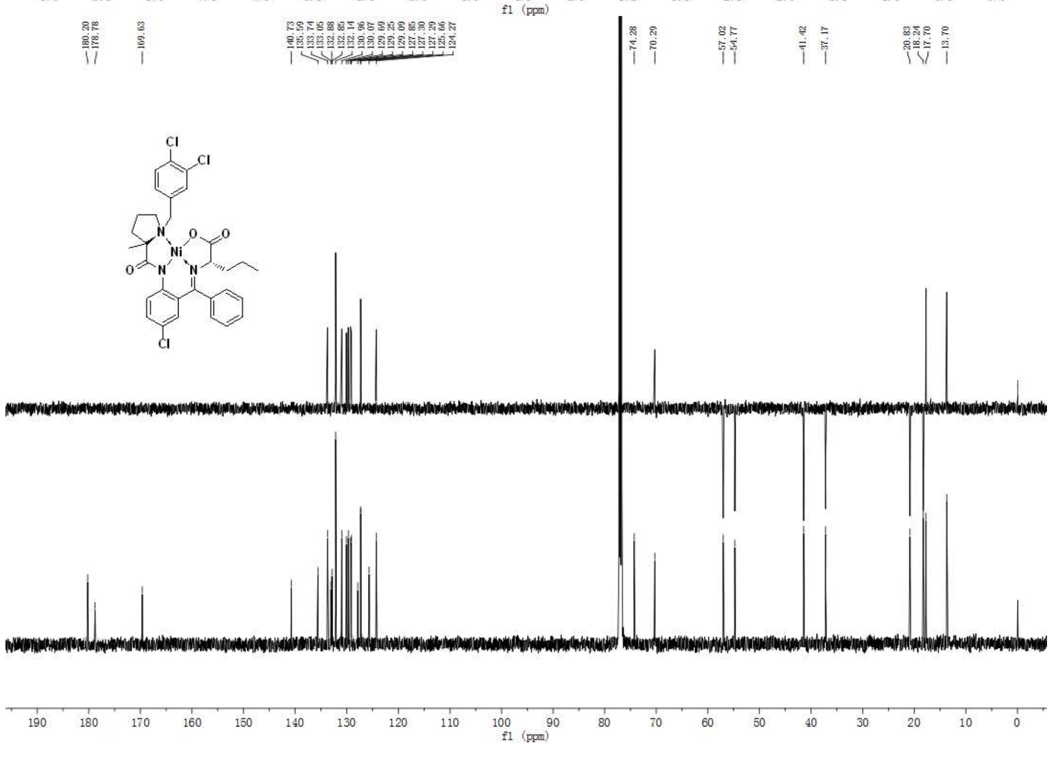

Figure S49: Nickel(II)-(*R*)-*N*-(2-benzoyl-4-chlorophenyl)-1-(3,4-dichlorobenzyl)-2-methylpyrrolidine-2-carboxamide/(*R*)-valine Schiff Base Complex 6o

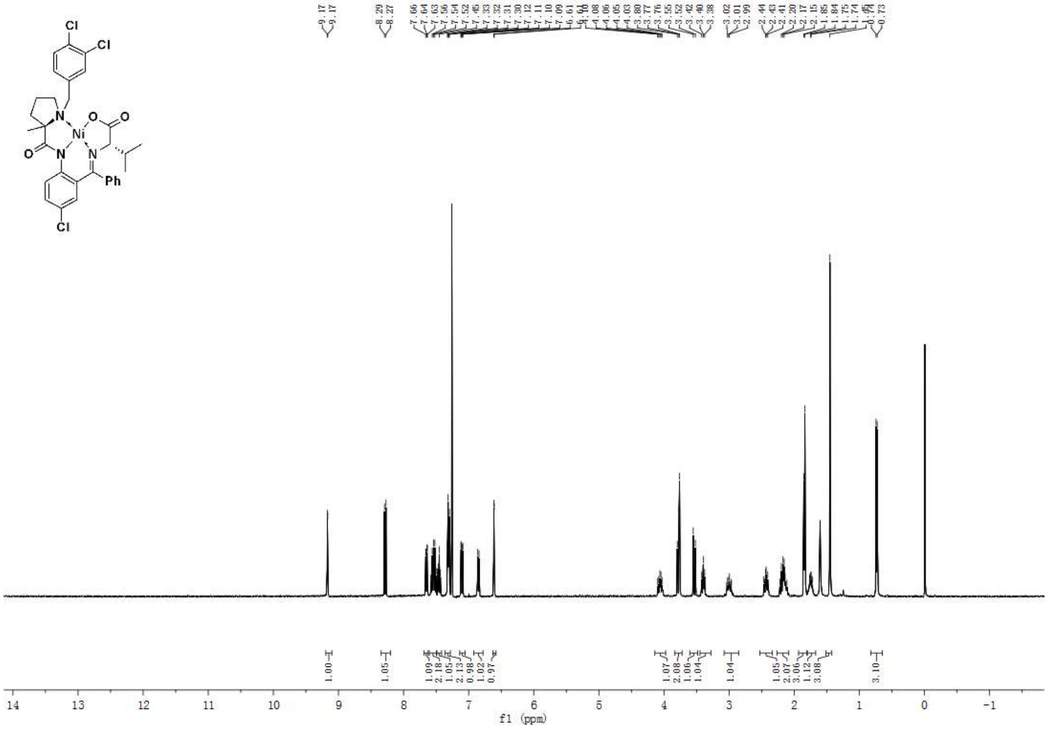

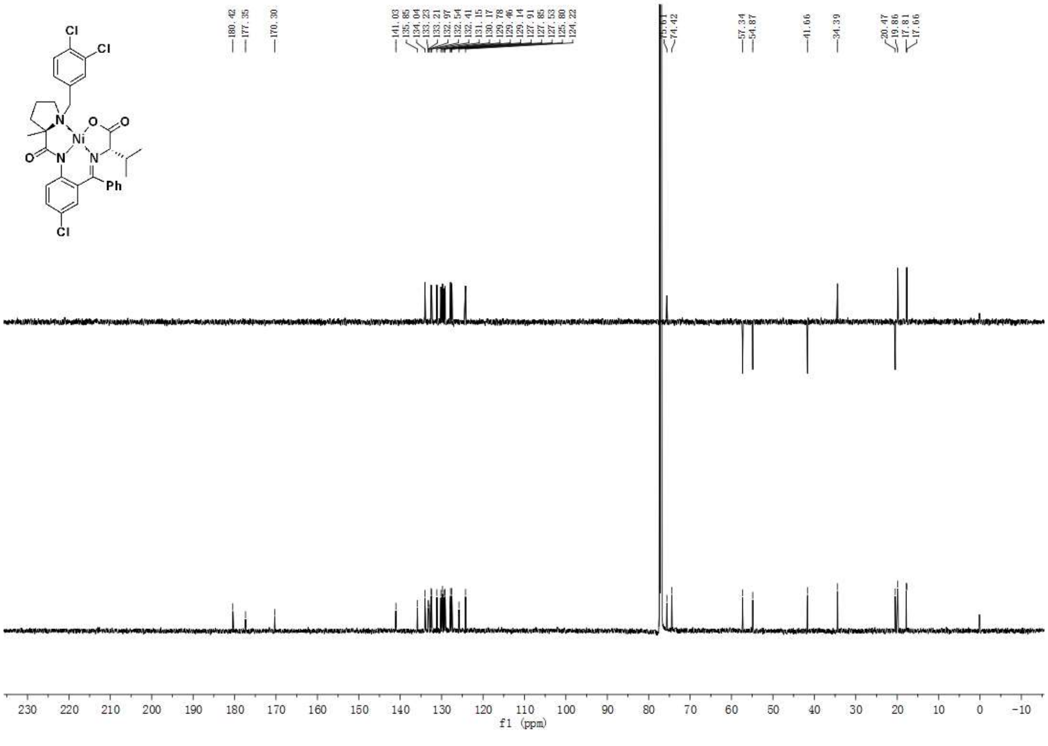

Figure S50: Nickel(II)-(*R*)-*N*-(2-benzoyl-4-chlorophenyl)-1-(3,4-dichlorobenzyl)-2-methylpyrrolidine-2-carboxamide/(*R*)-leucine Schiff Base Complex 6p

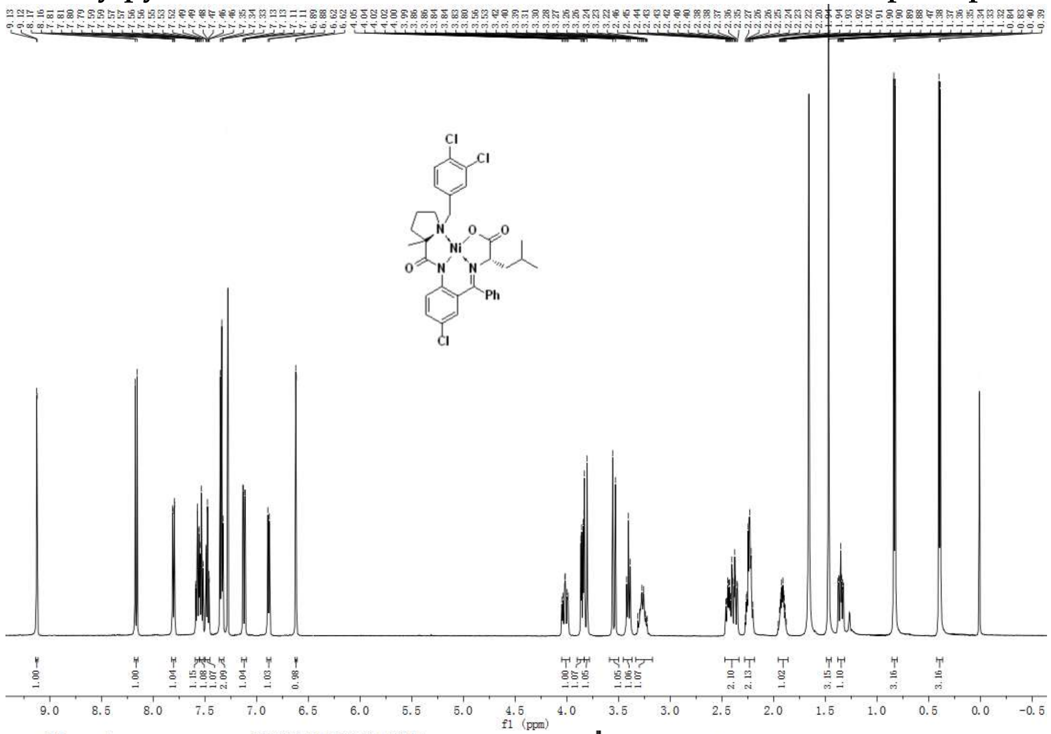

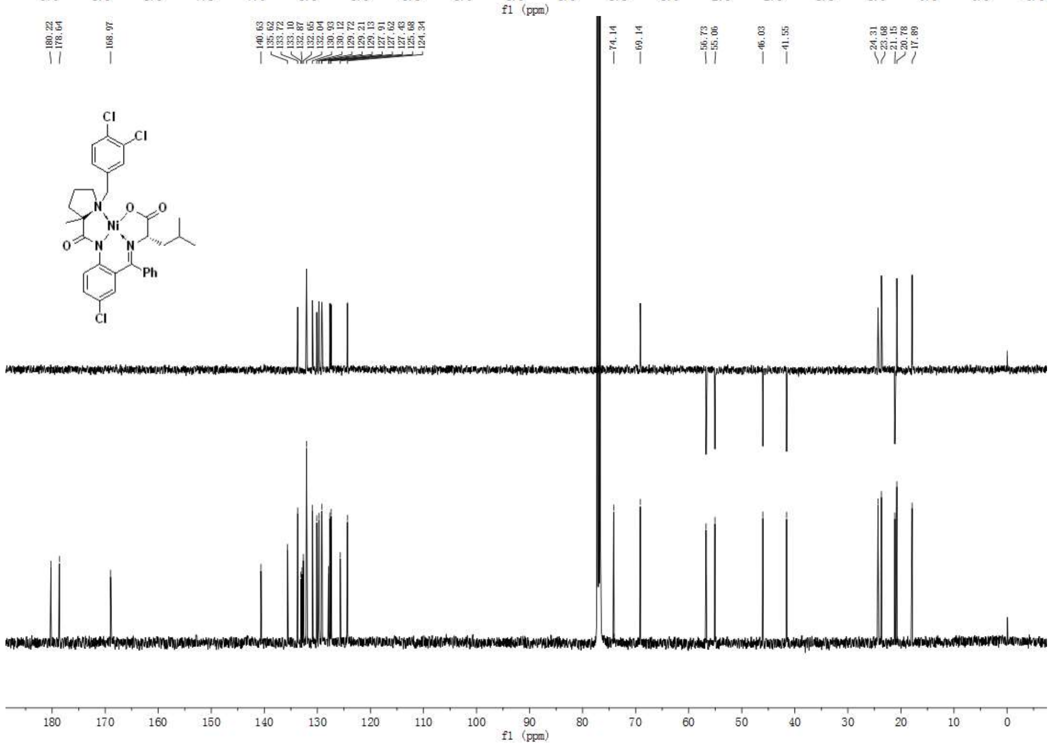

Figure S51: Nickel(II)-(*R*)-*N*-(2-benzoyl-4-chlorophenyl)-1-(3,4-dichlorobenzyl)-2-methylpyrrolidine-2-carboxamide/(*R*)-methionine Schiff Base Complex 6q

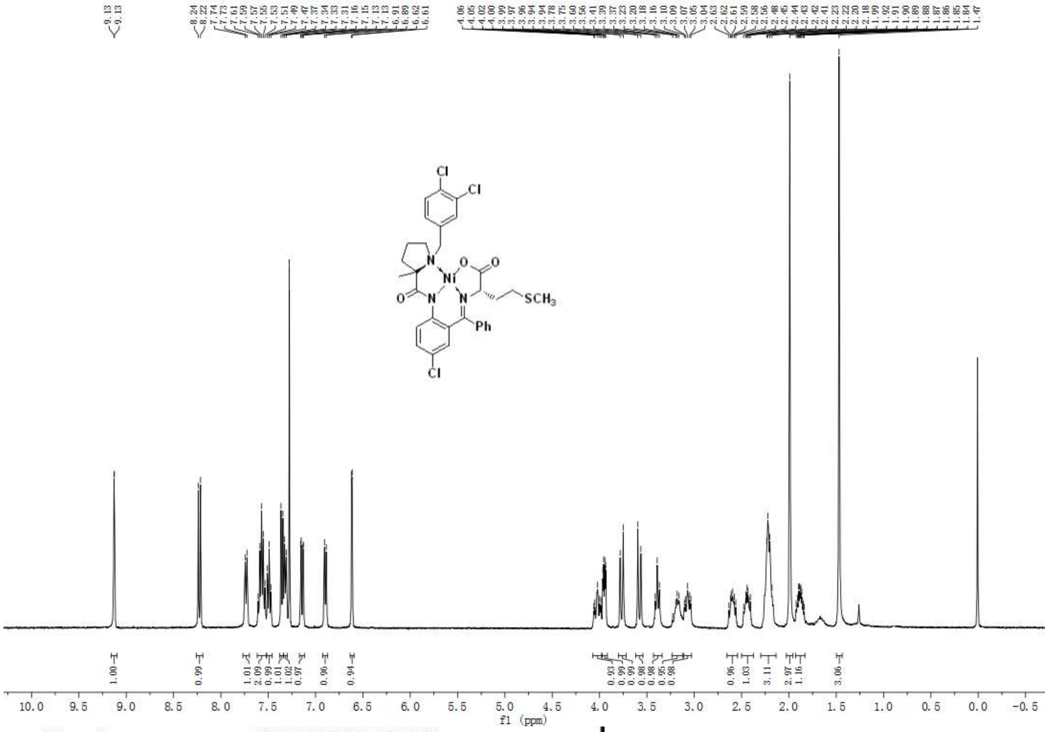

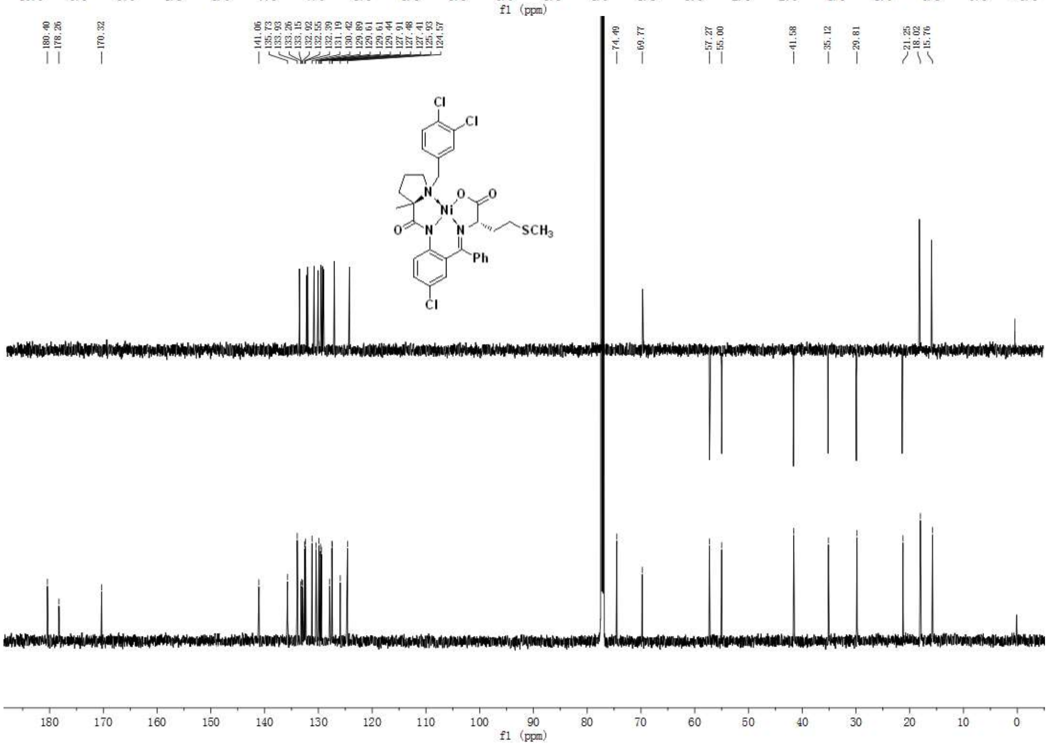

Figure S52: Nickel(II)-(*S*)-*N*-(2-benzoyl-4-chlorophenyl)-1-(3,4-dichlorobenzyl)-2-methylpyrrolidine-2-carboxamide/(*S*)-phenylalanine Schiff Base Complex 6a

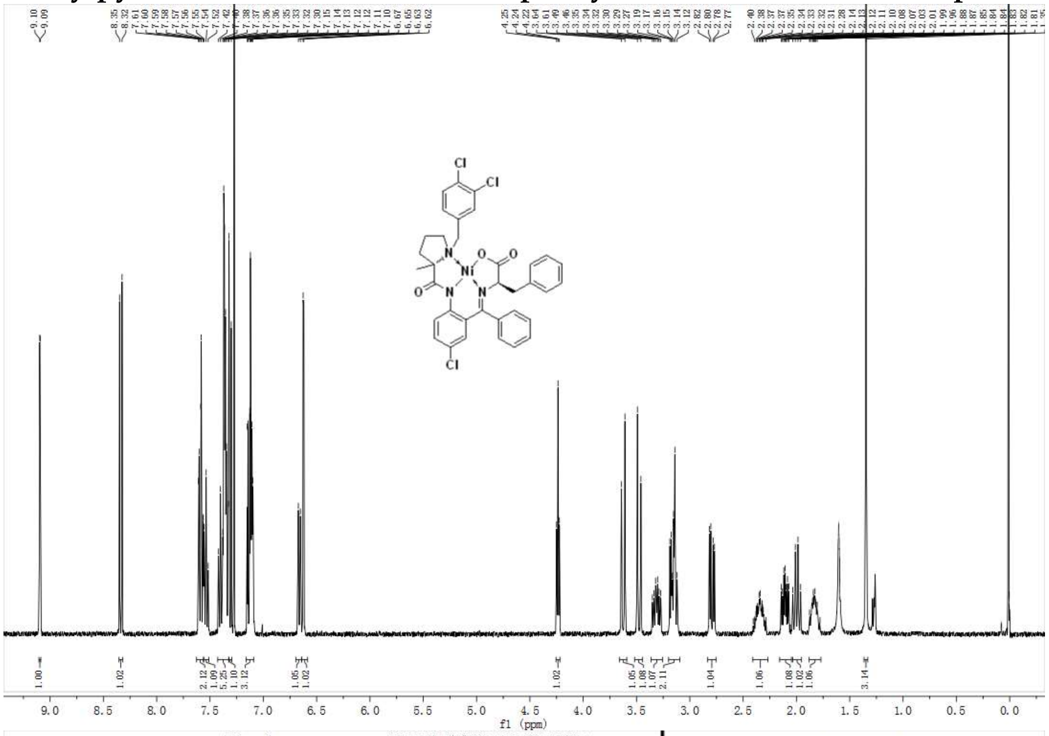

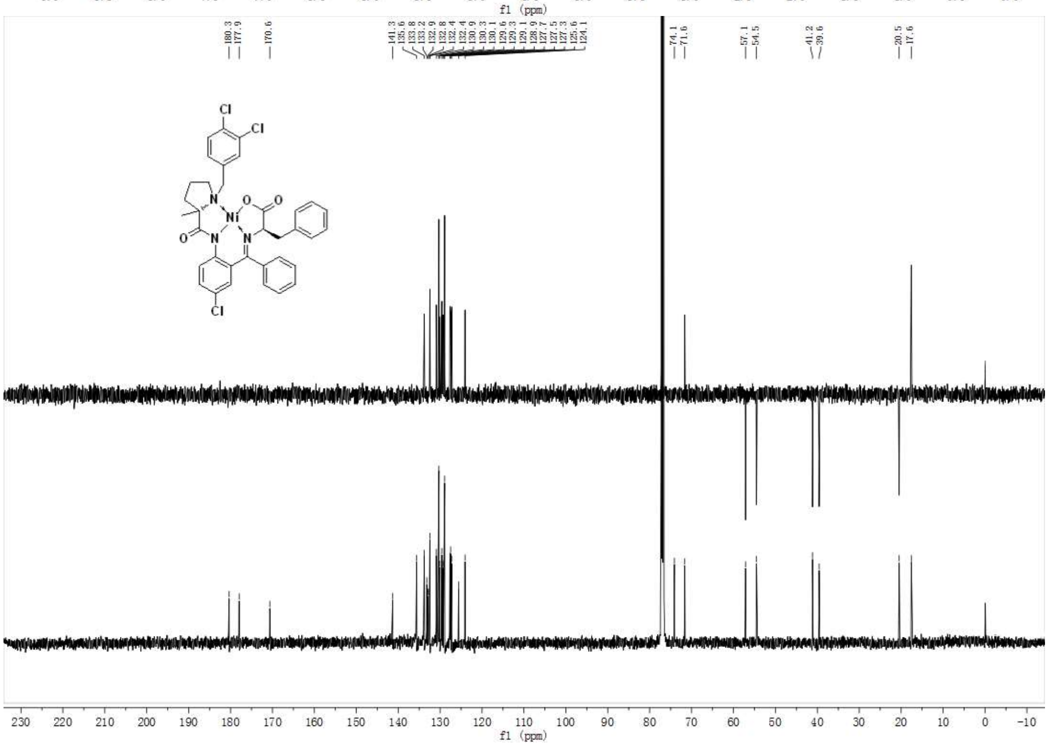

Figure S53: Nickel(II)-(*S*)-*N*-(2-benzoyl-4-chlorophenyl)-1-(3,4-dichlorobenzyl)-2-methylpyrrolidine-2-carboxamide/(*S*)-2-methoxyphenylalanine Schiff Base Complex 6b

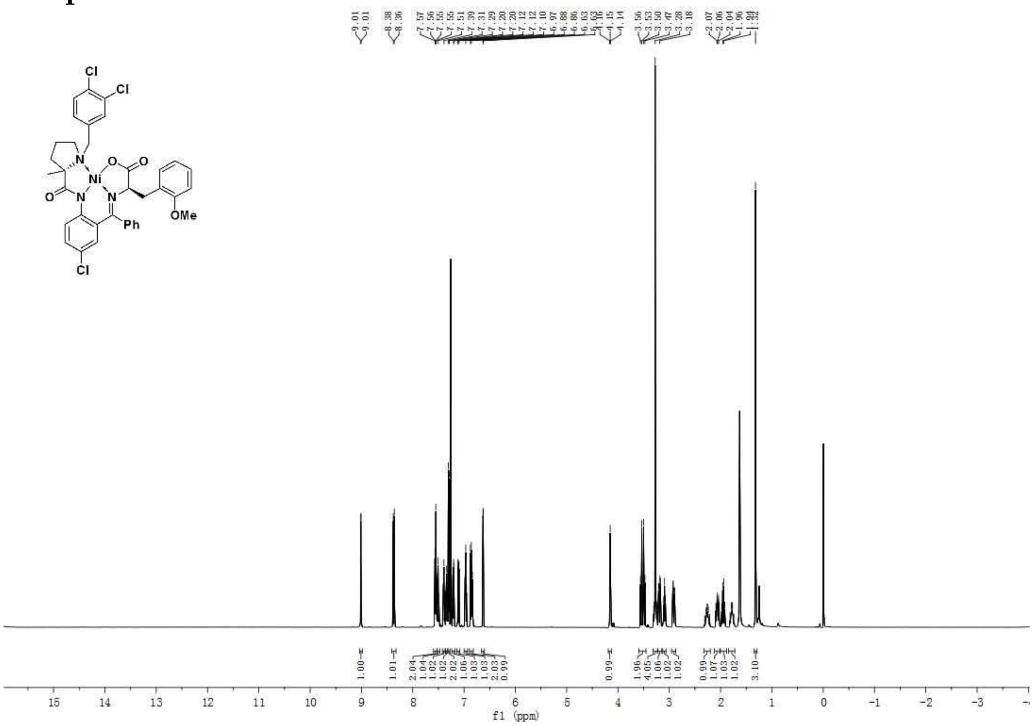

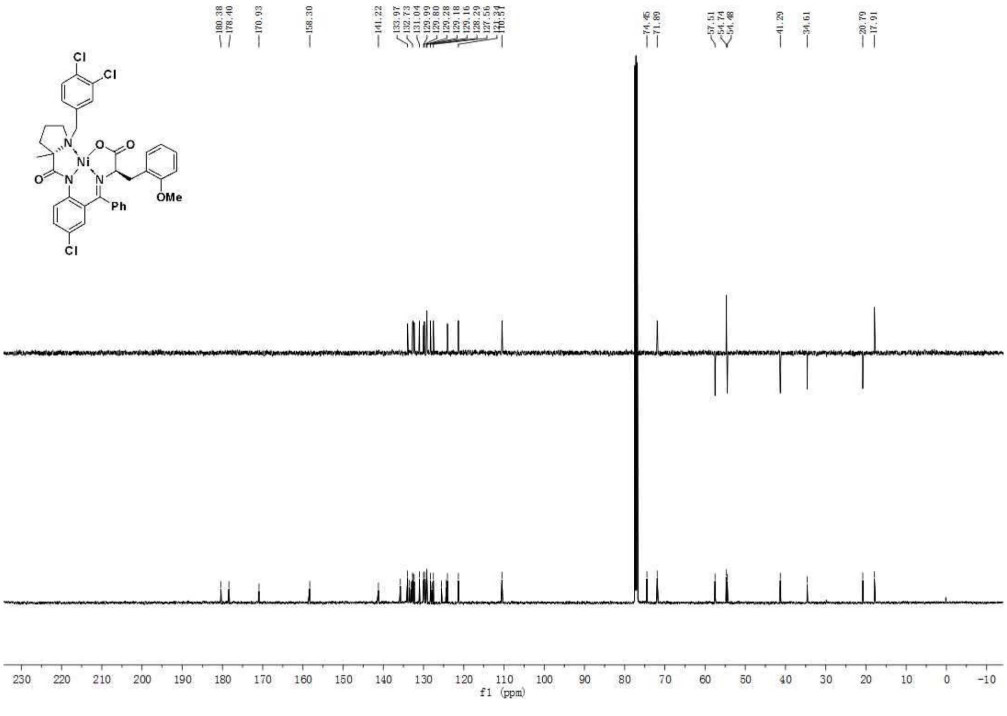

Figure S54: Nickel(II)-(*S*)-*N*-(2-benzoyl-4-chlorophenyl)-1-(3,4-dichlorobenzyl)-2-methylpyrrolidine-2-carboxamide/(*S*)-3-methoxyphenylalanine Schiff Base Complex 6c

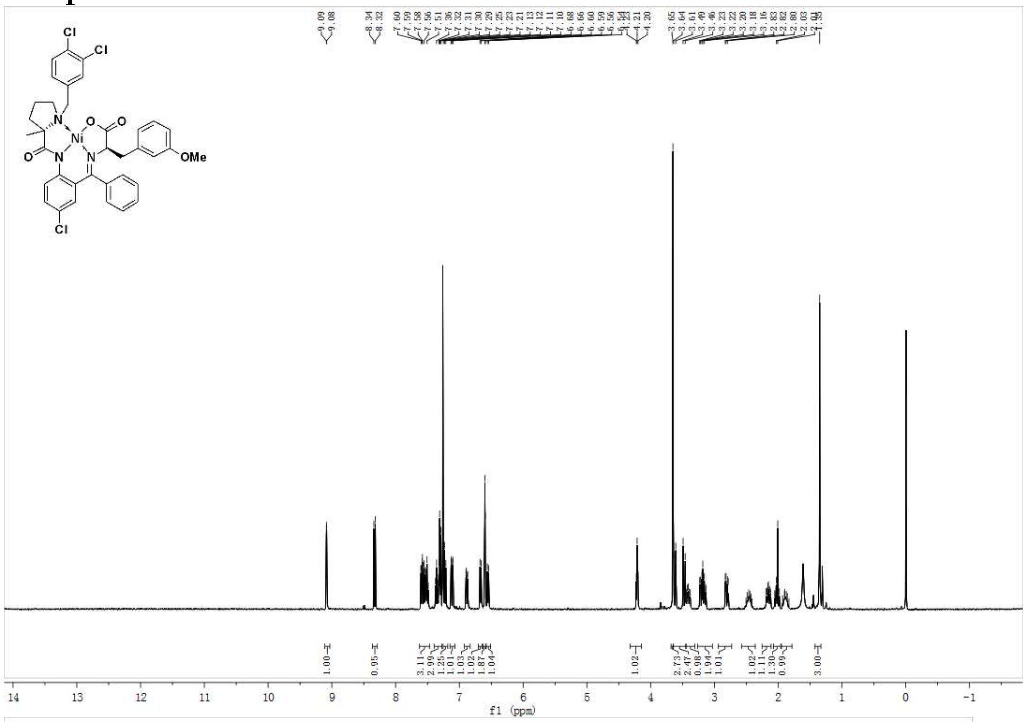

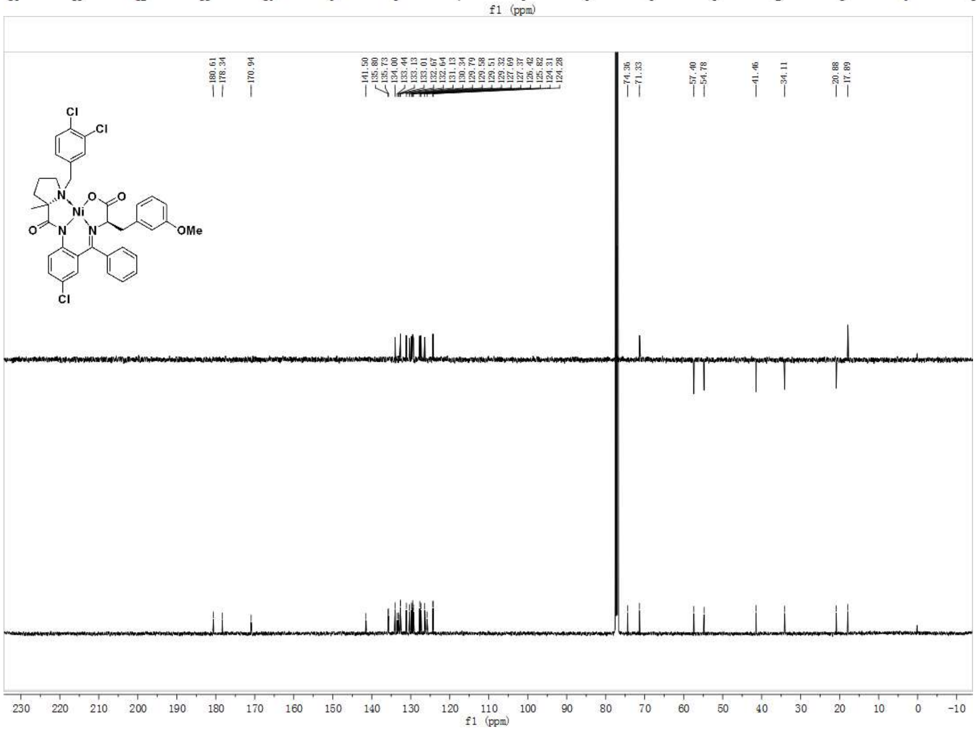

Figure S55: Nickel(II)-(*S*)-*N*-(2-benzoyl-4-chlorophenyl)-1-(3,4-dichlorobenzyl)-2-methylpyrrolidine-2-carboxamide/(*S*)-3-methylphenylalanine Schiff Base Complex 6d

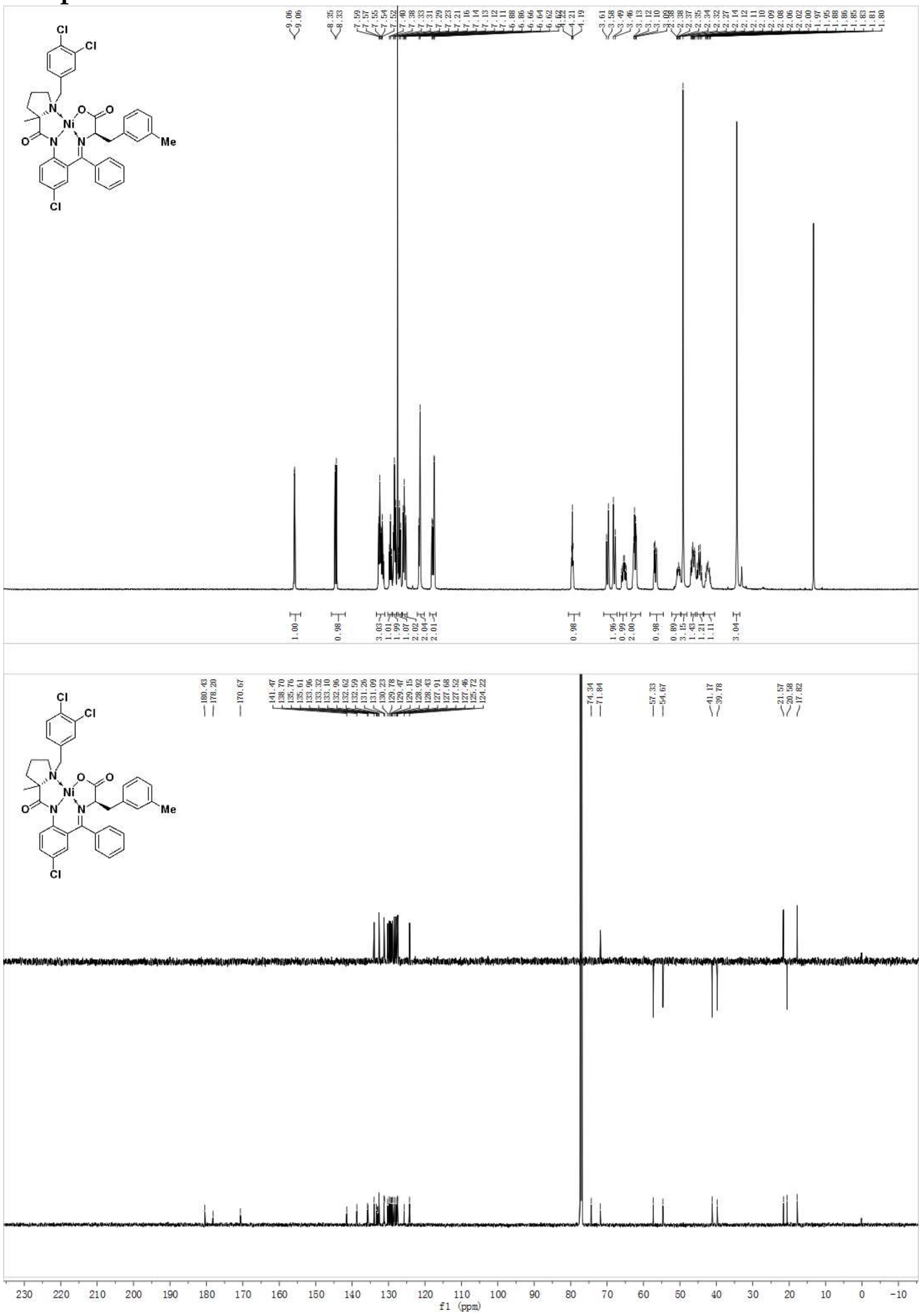

Figure S56: Nickel(II)-(*S*)-*N*-(2-benzoyl-4-chlorophenyl)-1-(3,4-dichlorobenzyl)-2-methylpyrrolidine-2-carboxamide/(*S*)-4-fluorophenylalanine Schiff Base Complex 6e

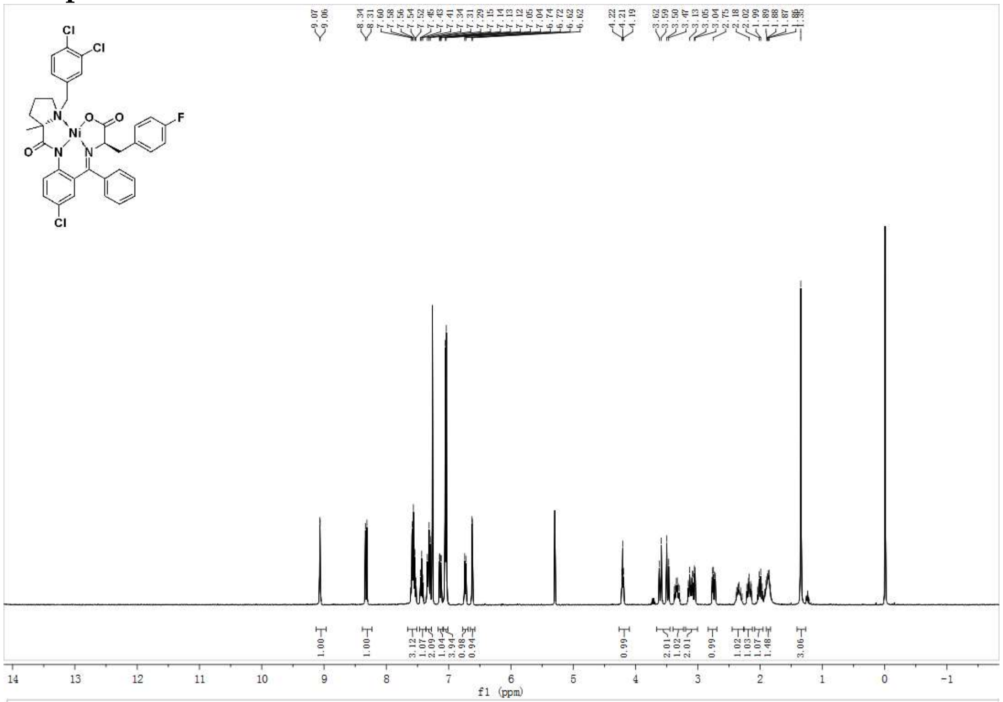

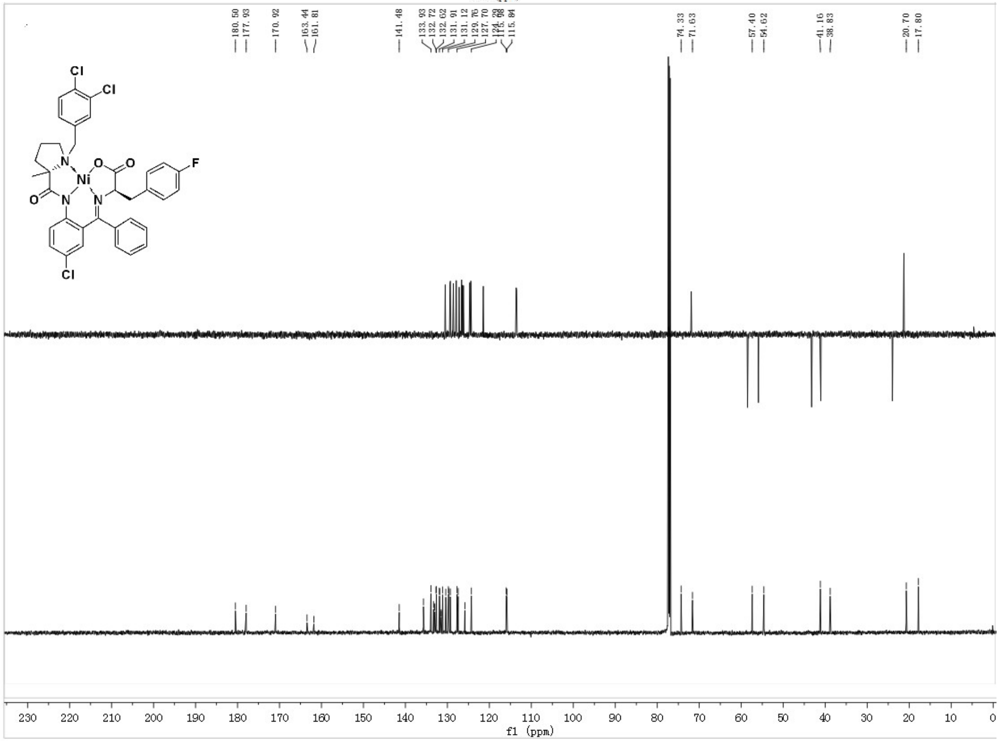

Figure S57: Nickel(II)-(*S*)-*N*-(2-benzoyl-4-chlorophenyl)-1-(3,4-dichlorobenzyl)-2-methylpyrrolidine-2-carboxamide/(*S*)-3,5-diiodotyrosine Schiff Base Complex 6f

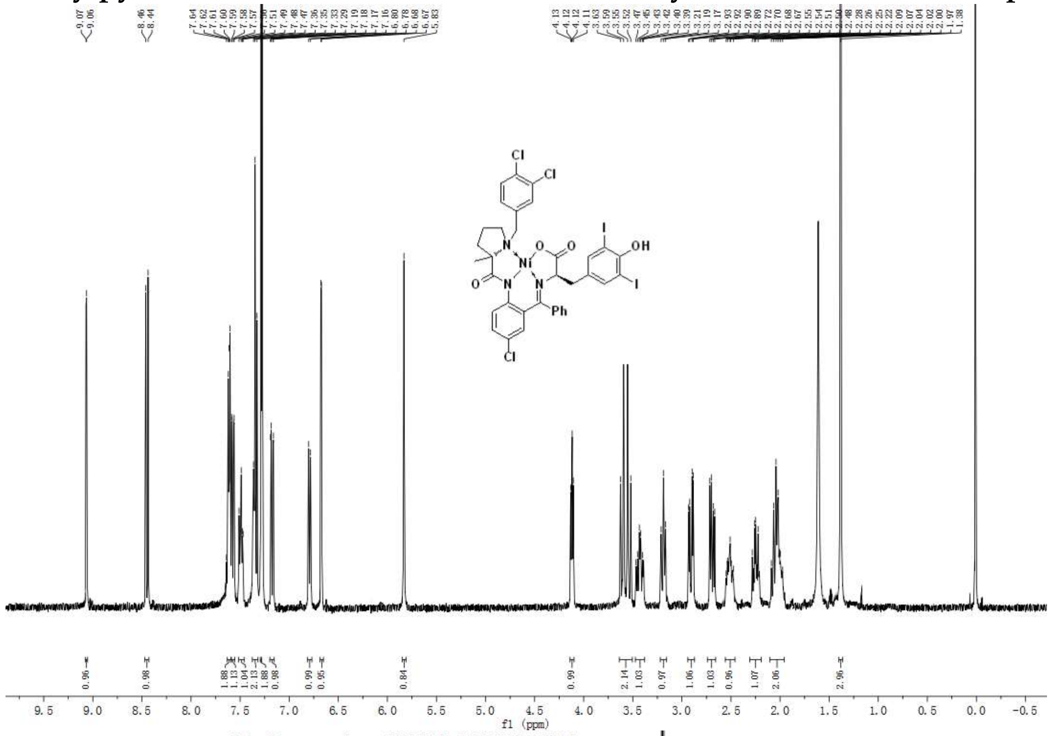

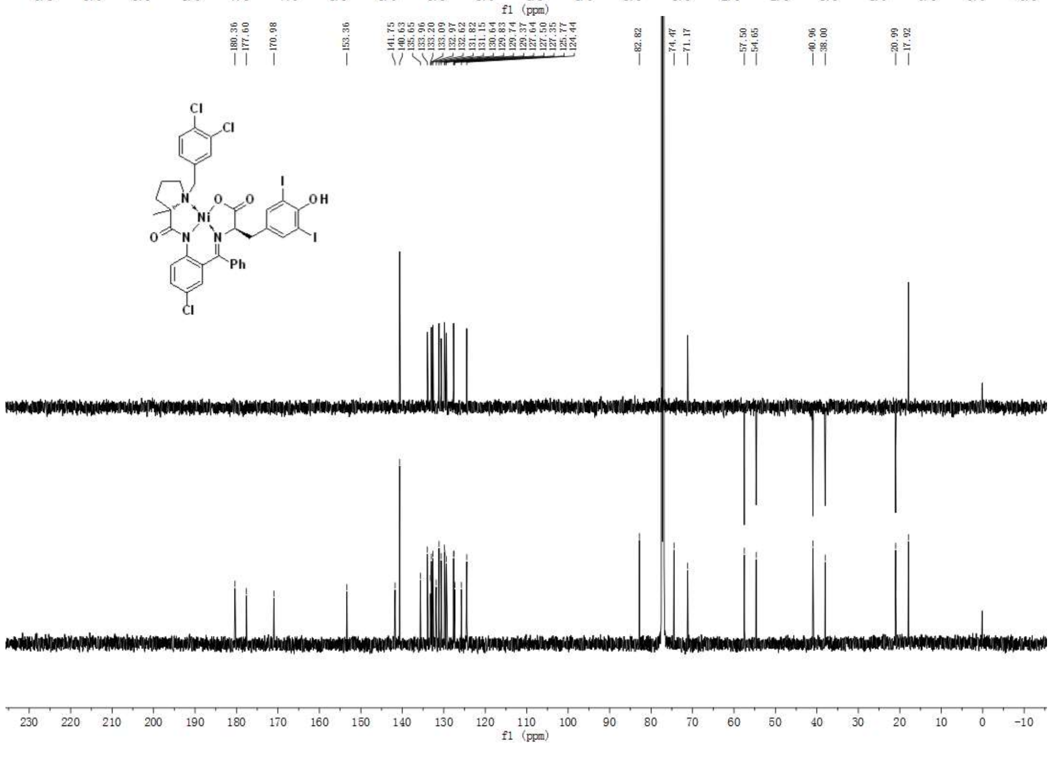

Figure S58: Nickel(II)-(*S*)-*N*-(2-benzoyl-4-chlorophenyl)-1-(3,4-dichlorobenzyl)-2-methylpyrrolidine-2-carboxamide/(*S*)-3-(1-naphthyl)alanine Schiff Base Complex 6g

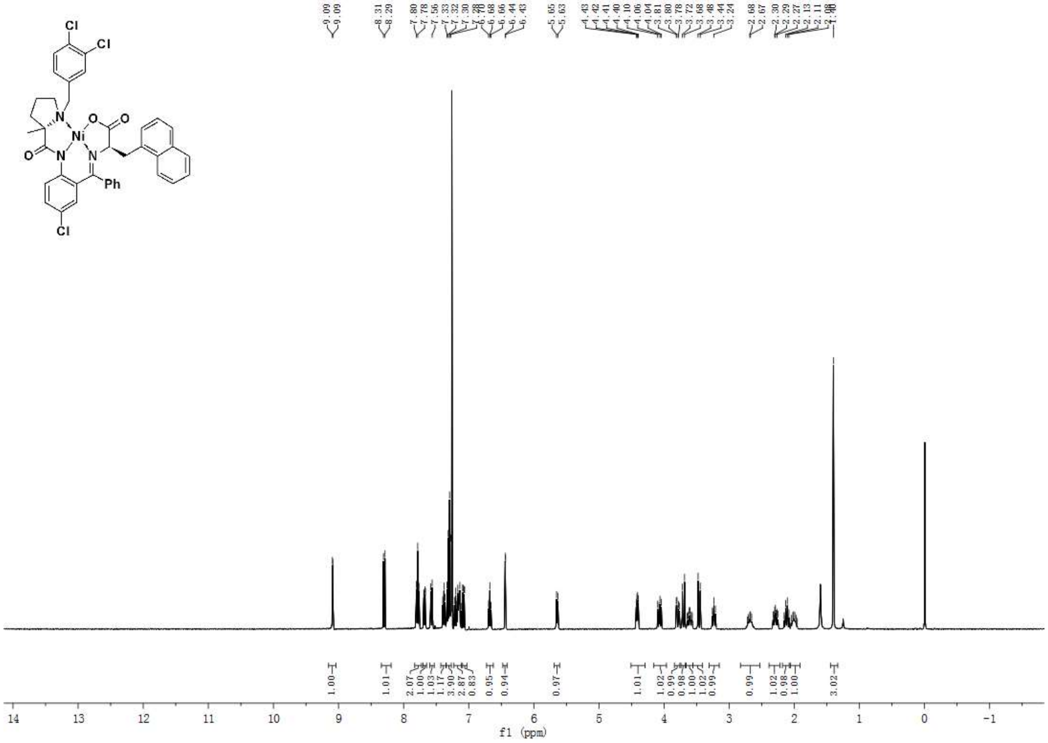

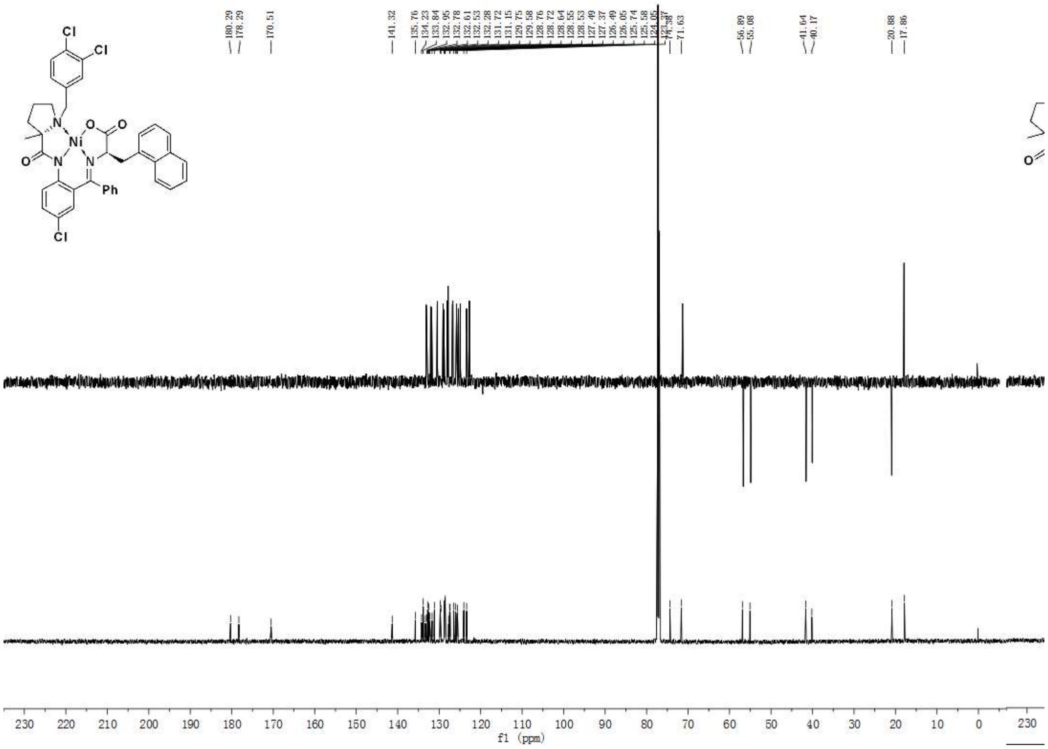

Figure S59: Nickel(II)-(*S*)-*N*-(2-benzoyl-4-chlorophenyl)-1-(3,4-dichlorobenzyl)-2-methylpyrrolidine-2-carboxamide/(*S*)-3-(3-benzothienyl)alanine Schiff Base Complex 6h

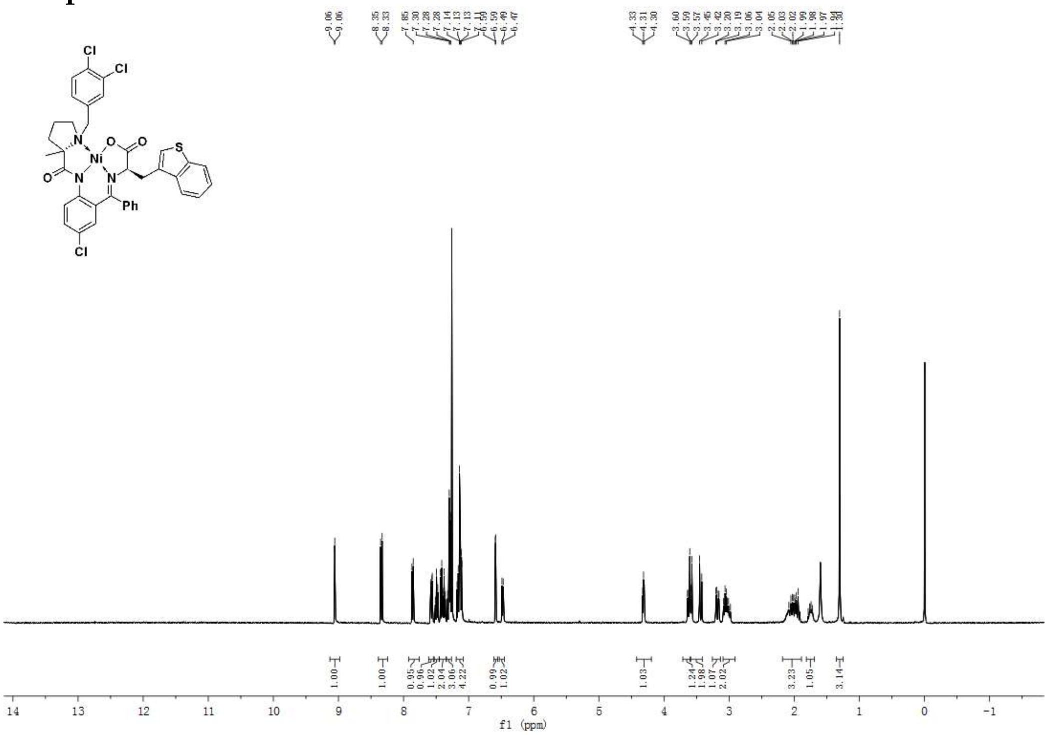

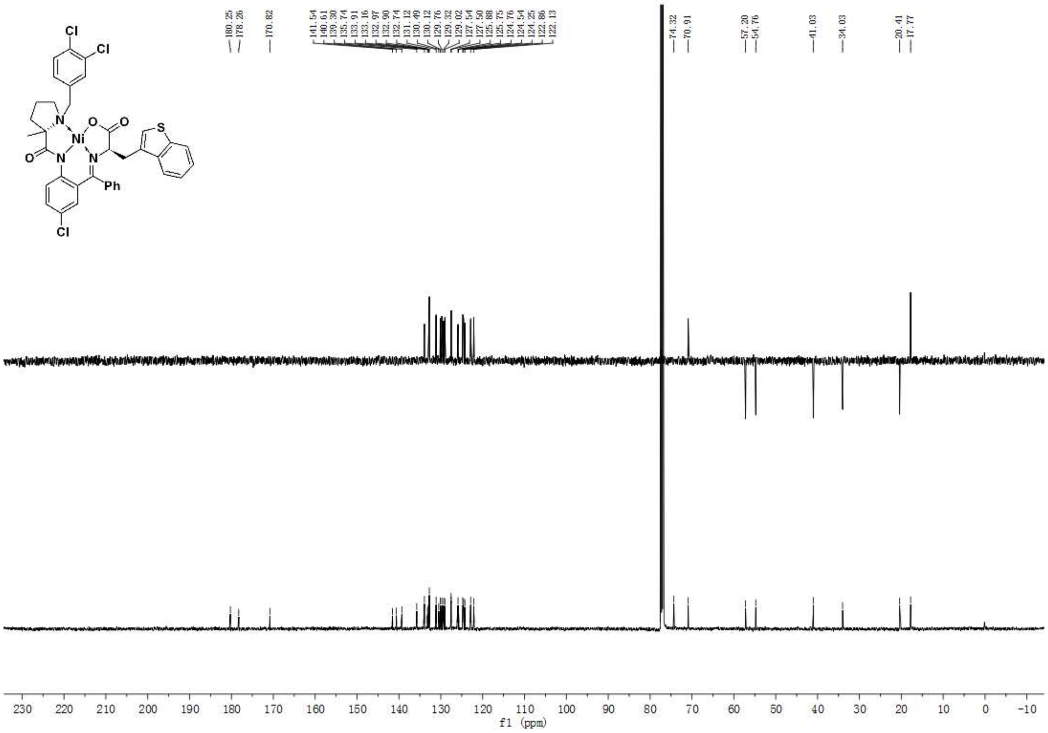

Figure S60: Nickel(II)-(*S*)-*N*-(2-benzoyl-4-chlorophenyl)-1-(3,4-dichlorobenzyl)-2-methylpyrrolidine-2-carboxamide/(*S*)-3-(3-thienyl)alanine Schiff Base Complex 6i

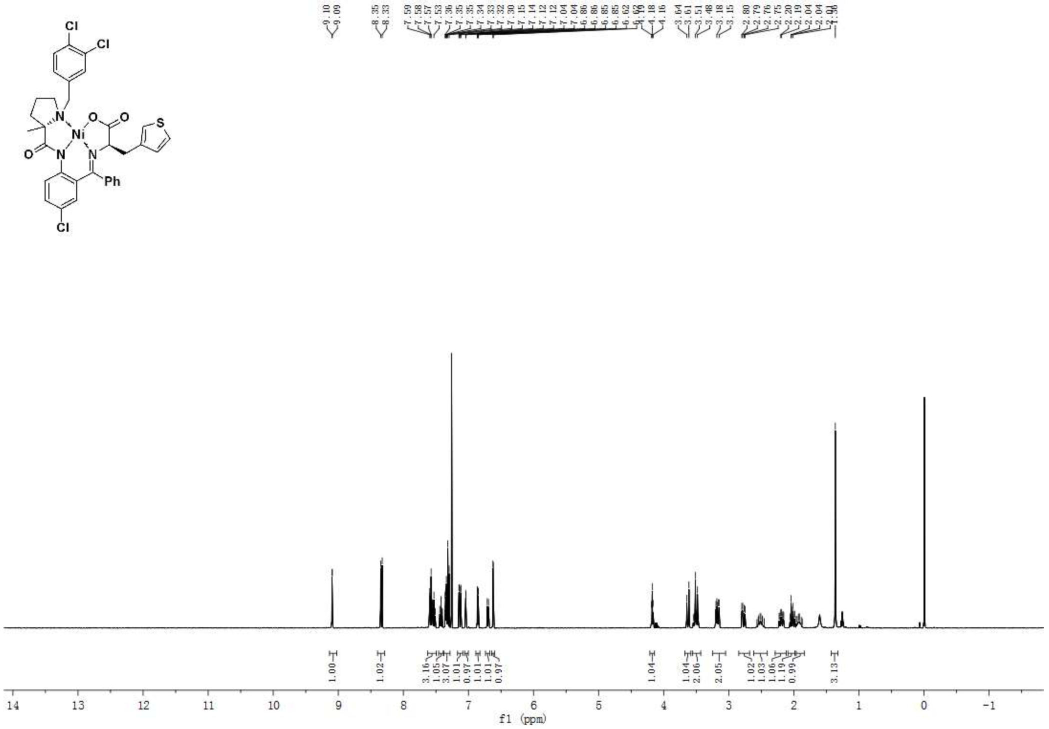

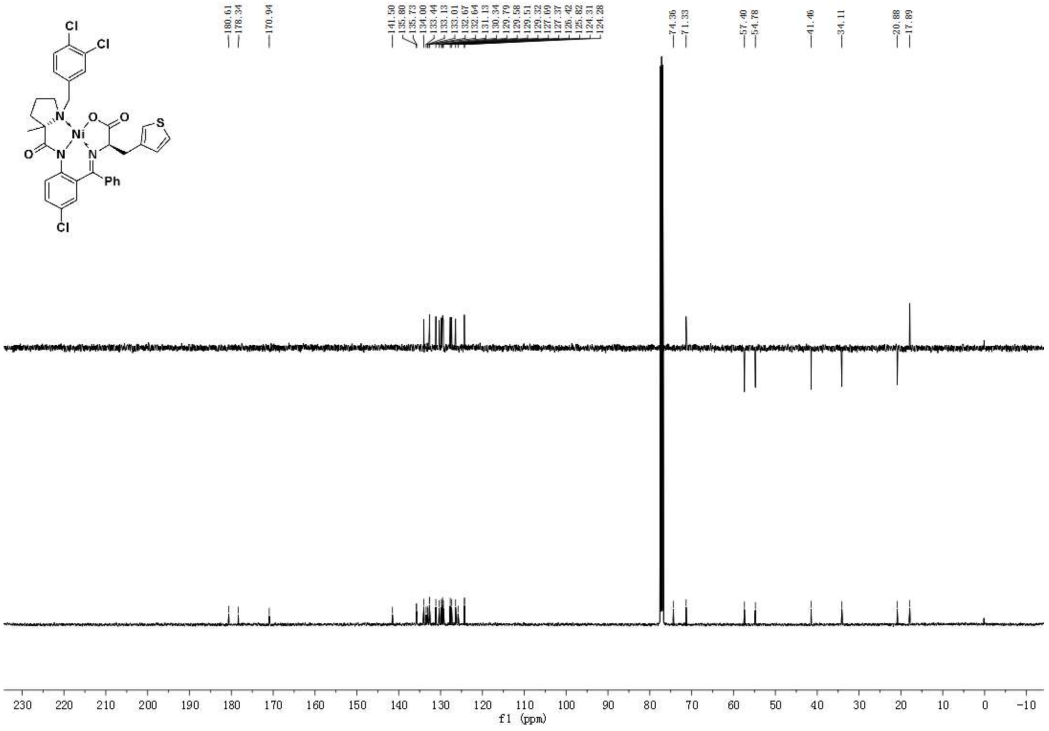

Figure S61: Nickel(II)-(*S*)-*N*-(2-benzoyl-4-chlorophenyl)-1-(3,4-dichlorobenzyl)-2-methylpyrrolidine-2-carboxamide/(*S*)-2-(3-methoxyphenyl)glycine Schiff Base Complex 6j

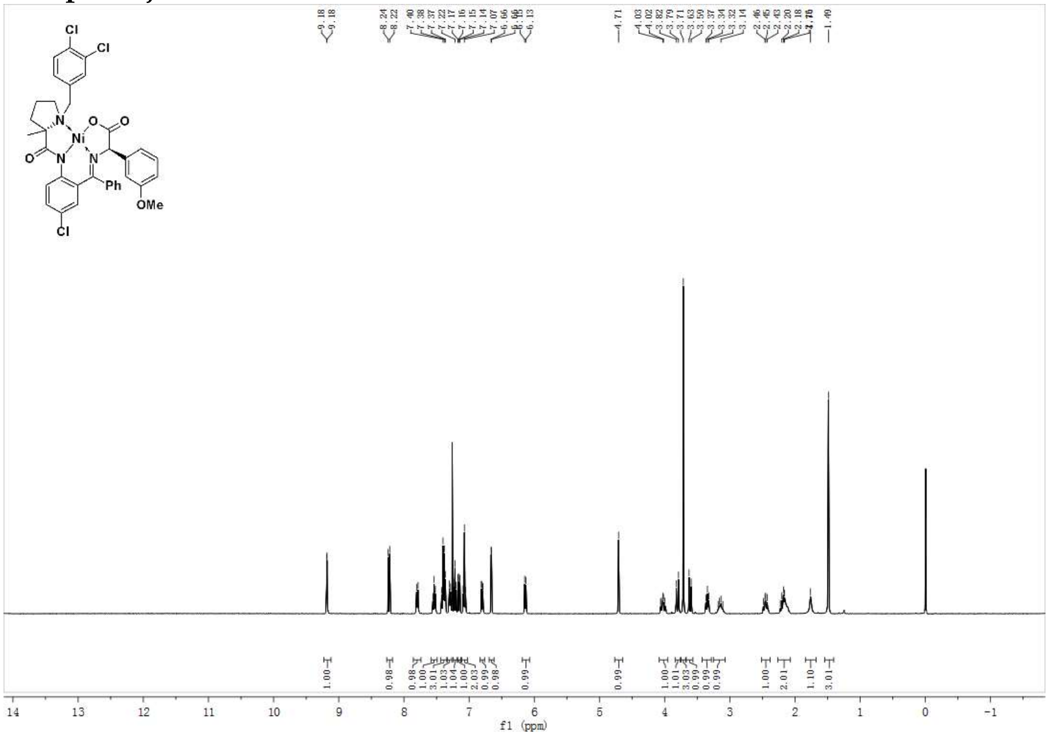

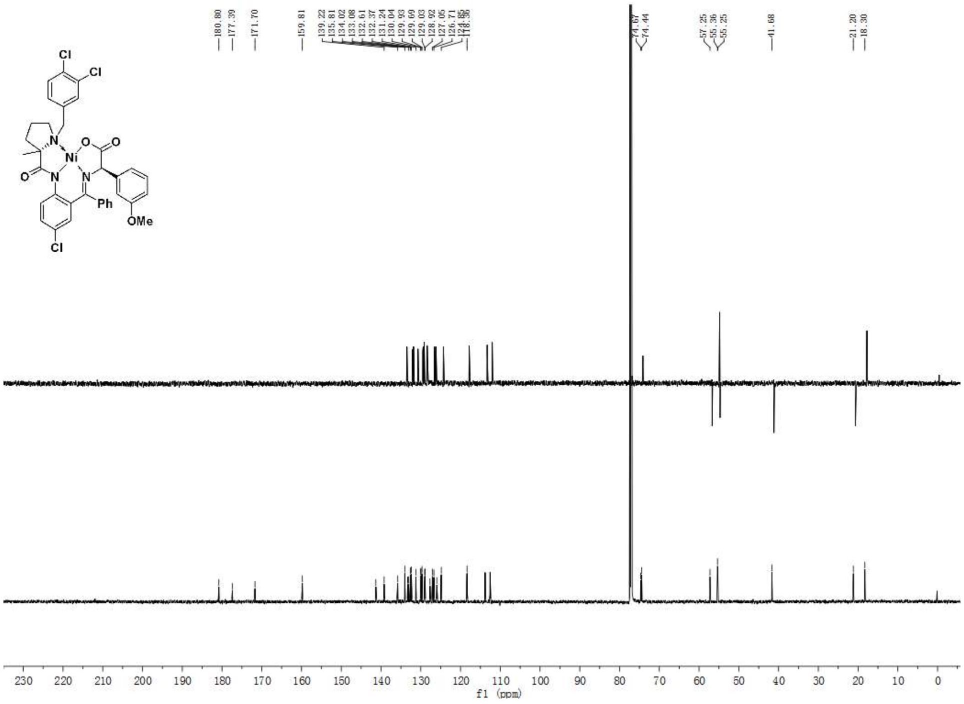

Figure S62 Nickel(II)-(*S*)-*N*-(2-benzoyl-4-chlorophenyl)-1-(3,4-dichlorobenzyl)-2-methylpyrrolidine-2-carboxamide/(*S*)-2-(3-bromophenyl)glycine Schiff Base Complex 6k

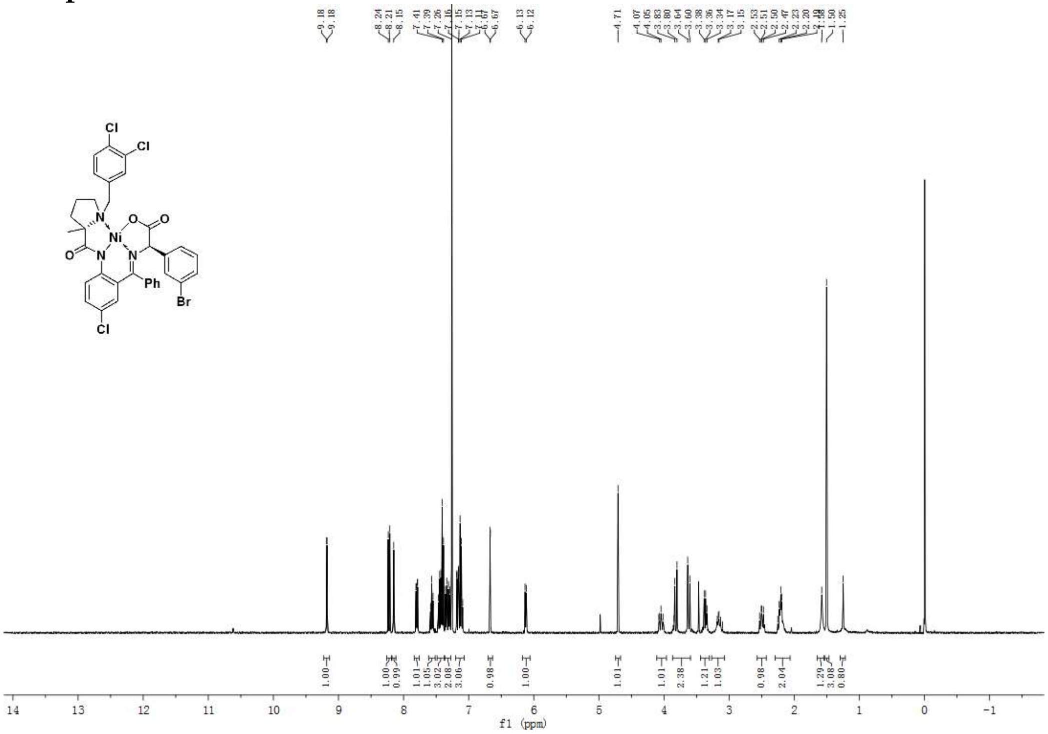

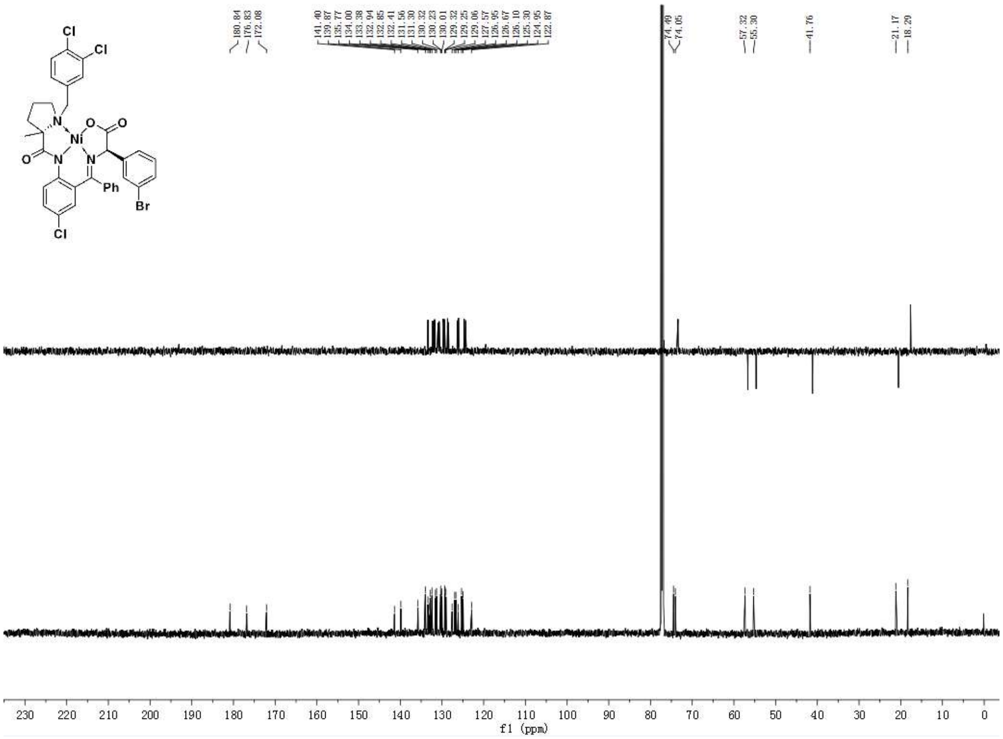

Figure S63: Nickel(II)-(*S*)-*N*-(2-benzoyl-4-chlorophenyl)-1-(3,4-dichlorobenzyl)-2-methylpyrrolidine-2-carboxamide/(*S*)-2-cyclobutylglycine Schiff Base Complex 6l

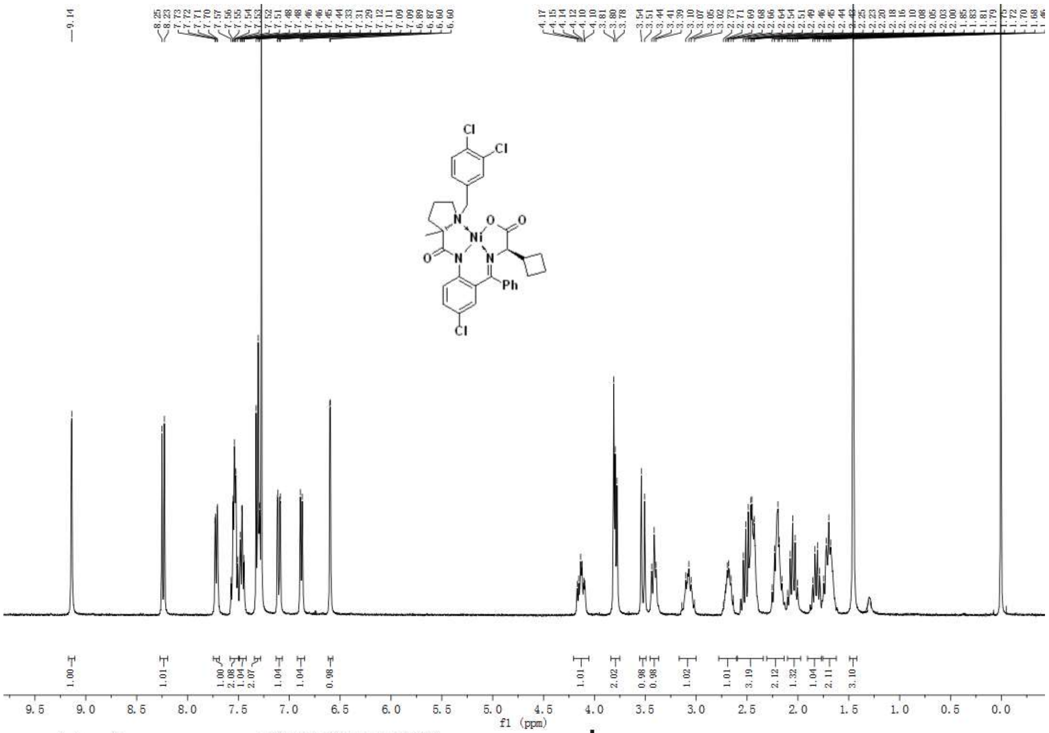

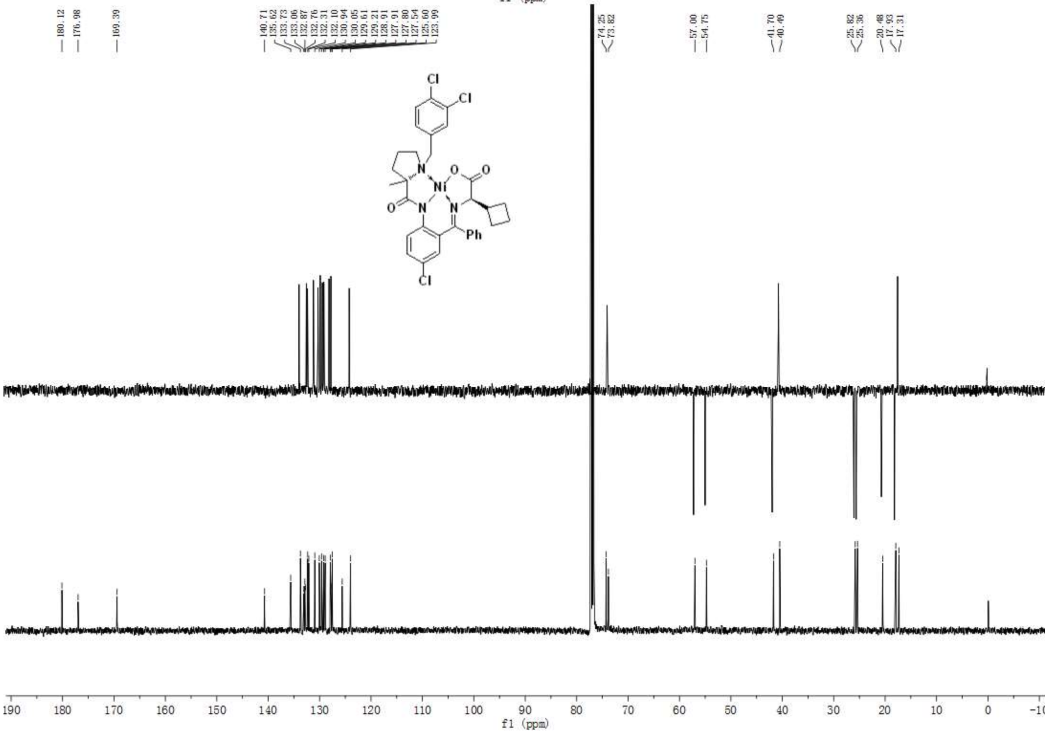

Figure S64: Nickel(II)-(*S*)-*N*-(2-benzoyl-4-chlorophenyl)-1-(3,4-dichlorobenzyl)-2-methylpyrrolidine-2-carboxamide/(*S*)-2-amino-4,4,4-trifluorobutyric acid Schiff Base Complex 6m

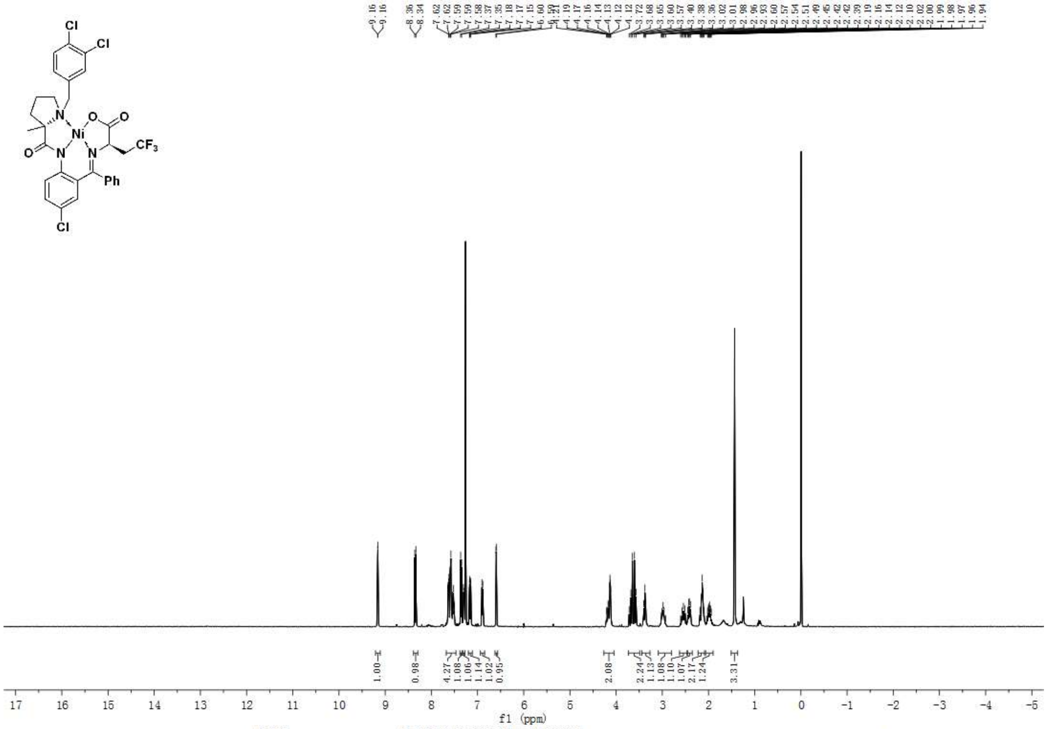

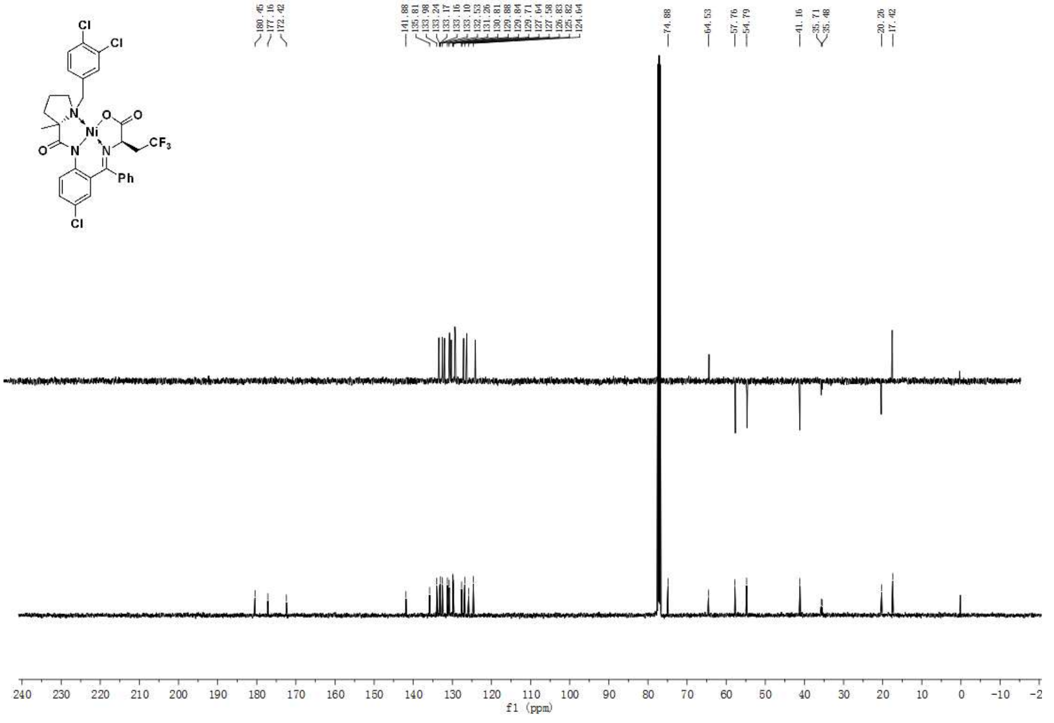

Figure S65: Nickel(II)-(*S*)-*N*-(2-benzoyl-4-chlorophenyl)-1-(3,4-dichlorobenzyl)-2-methylpyrrolidine-2-carboxamide/(*S*)-norvaline Schiff Base Complex 6n

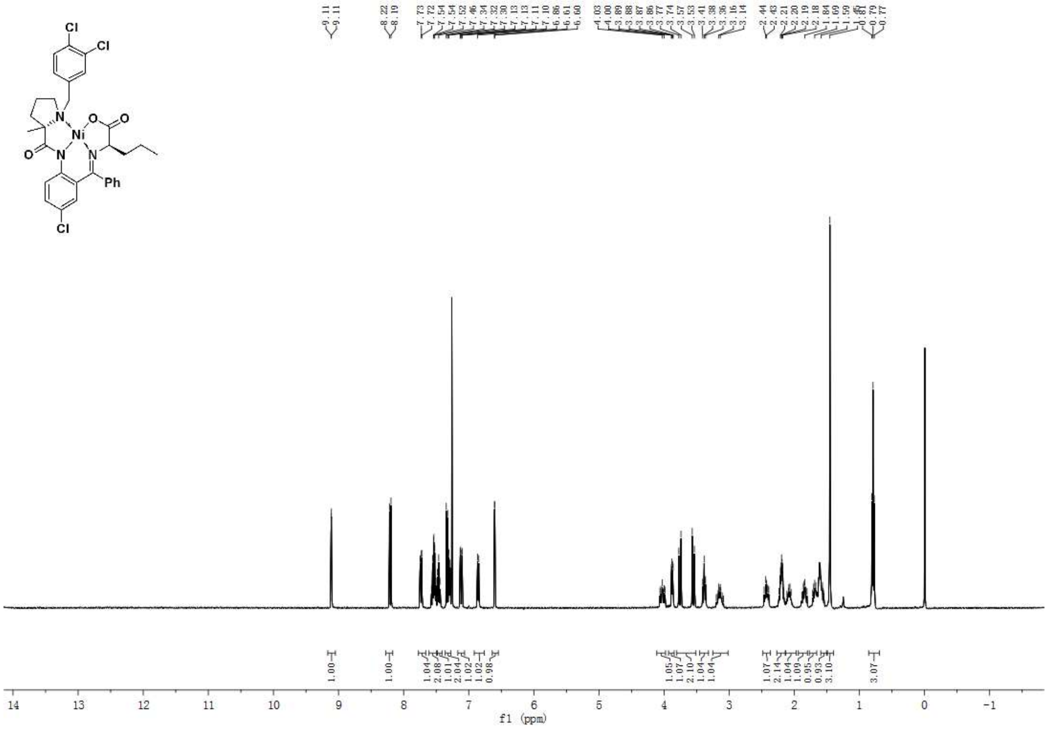

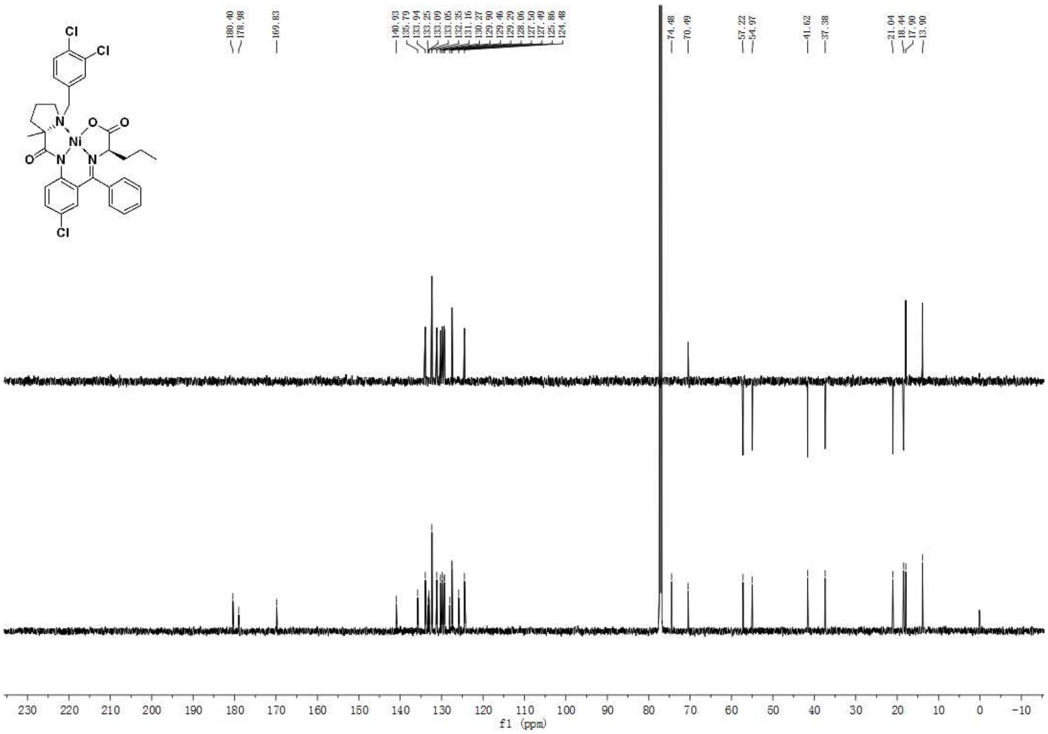

Figure S66: Nickel(II)-(*S*)-*N*-(2-benzoyl-4-chlorophenyl)-1-(3,4-dichlorobenzyl)-2-methylpyrrolidine-2-carboxamide/(*S*)-valine Schiff Base Complex 6o

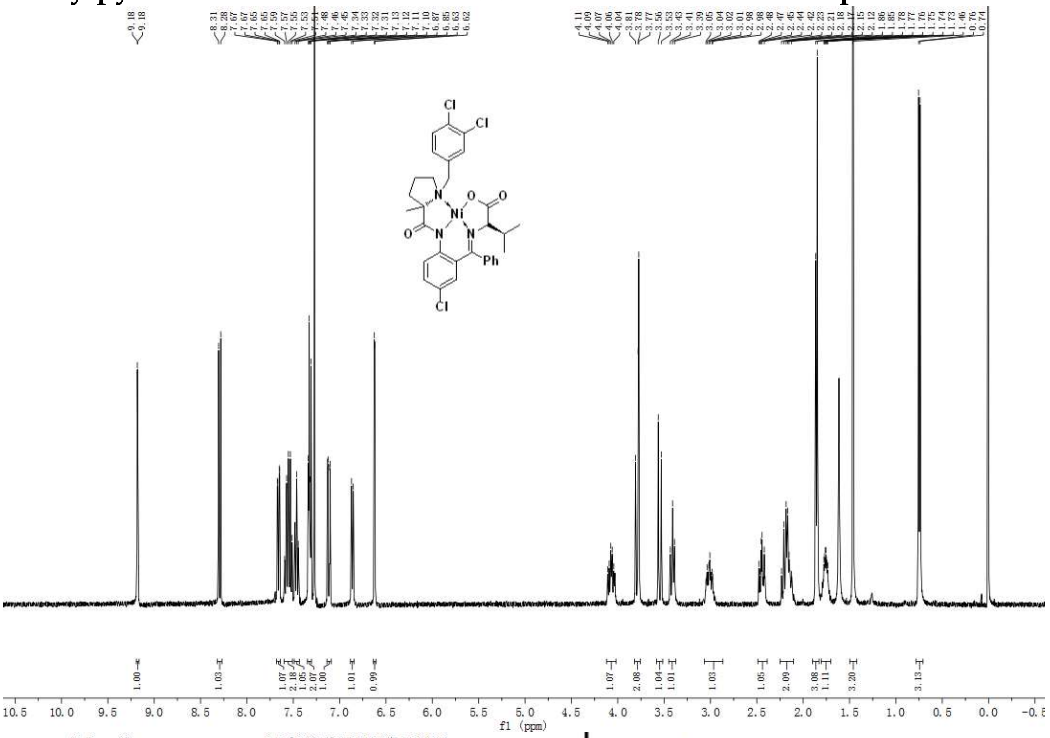

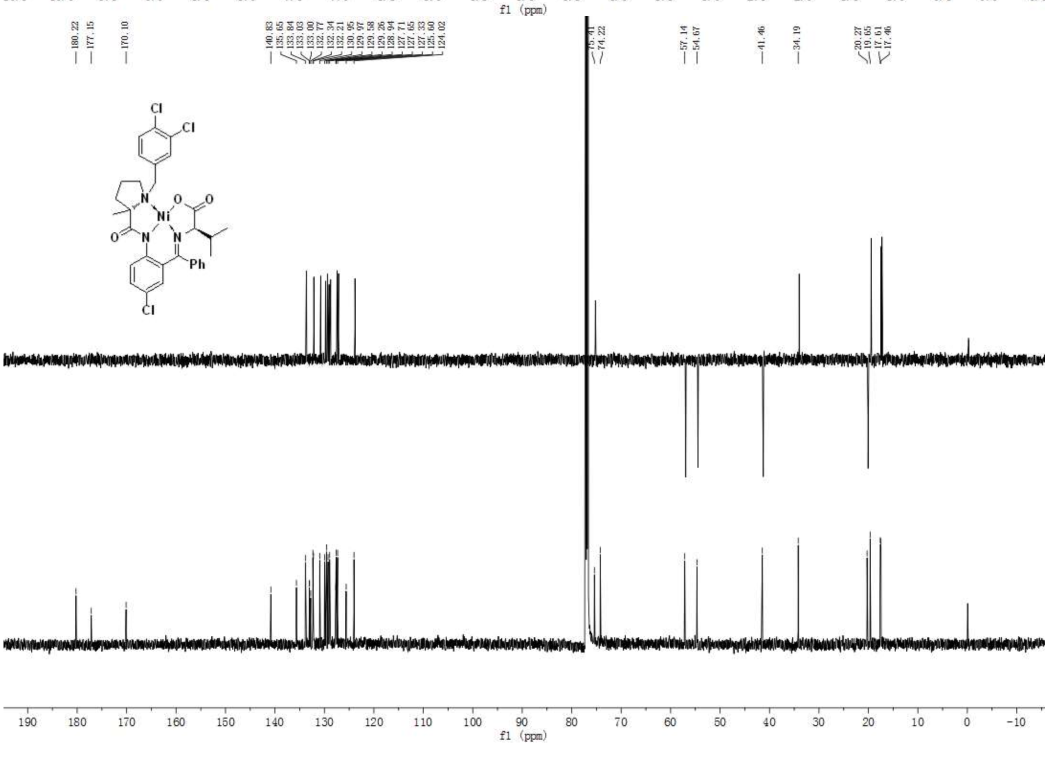

Figure S67: Nickel(II)-(*S*)-*N*-(2-benzoyl-4-chlorophenyl)-1-(3,4-dichlorobenzyl)-2-methylpyrrolidine-2-carboxamide/(*S*)-leucine Schiff Base Complex 6p

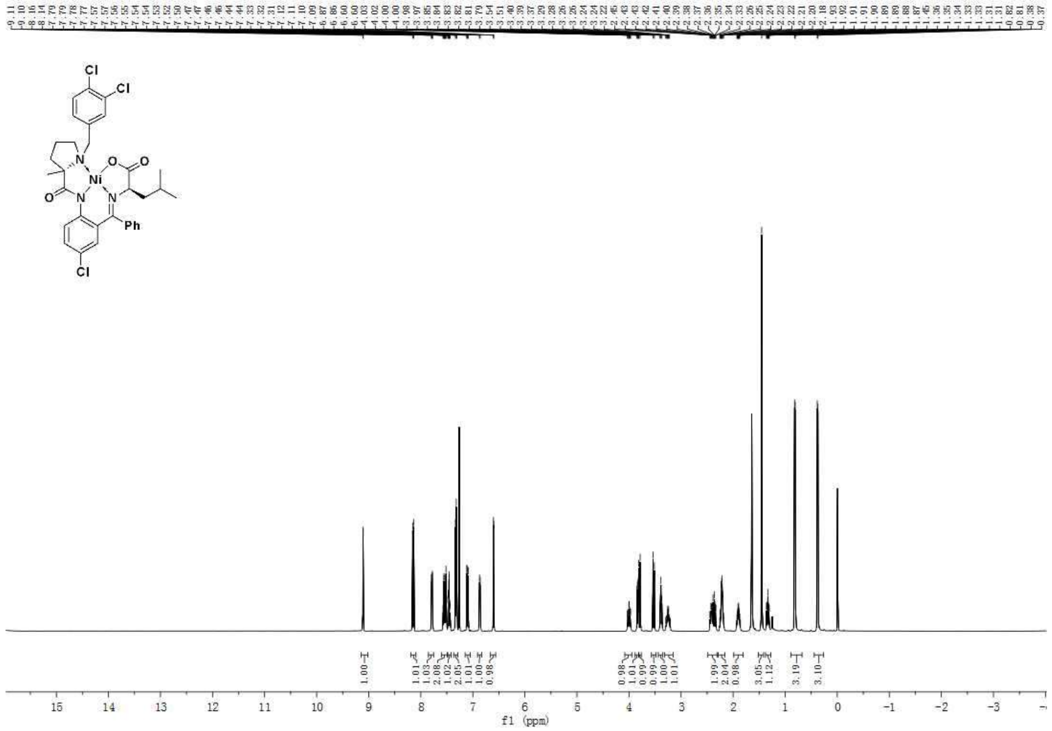

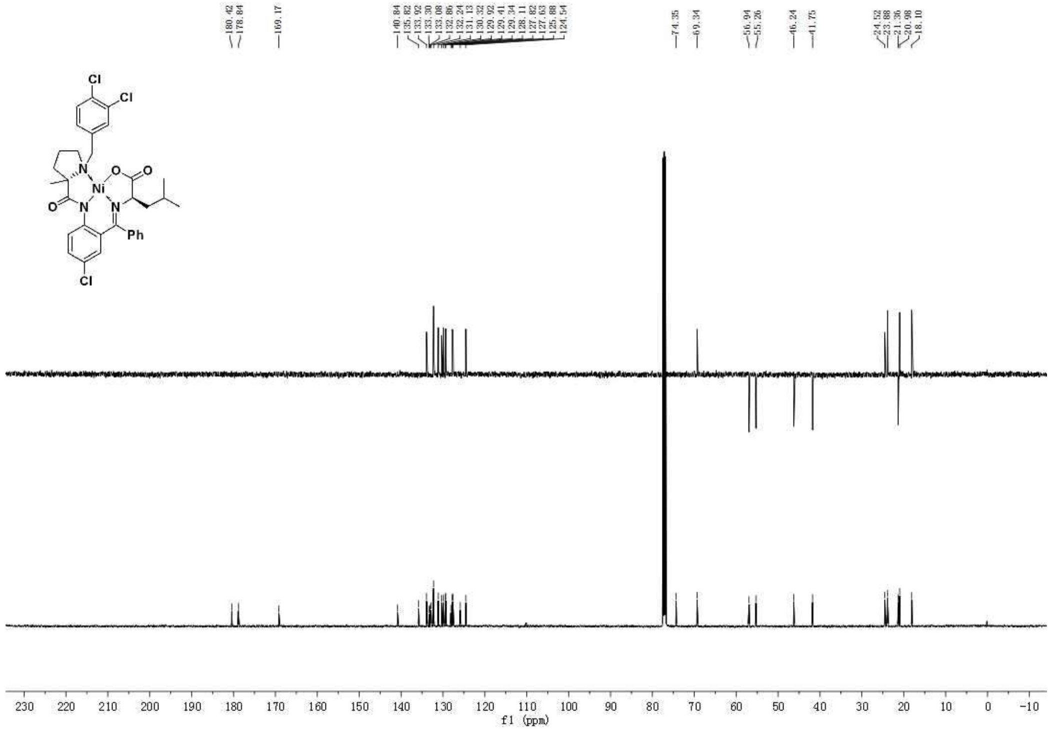

Figure S68: Nickel(II)-(*S*)-*N*-(2-benzoyl-4-chlorophenyl)-1-(3,4-dichlorobenzyl)-2-methylpyrrolidine-2-carboxamide/(*S*)-methionine Schiff Base Complex 6q

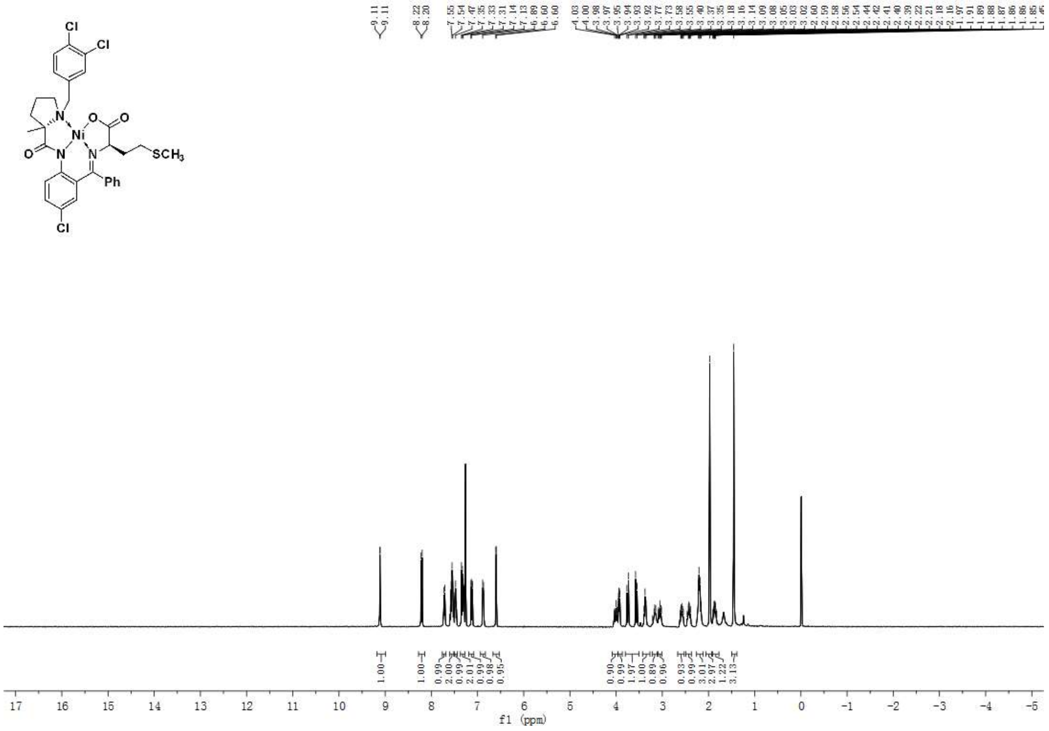

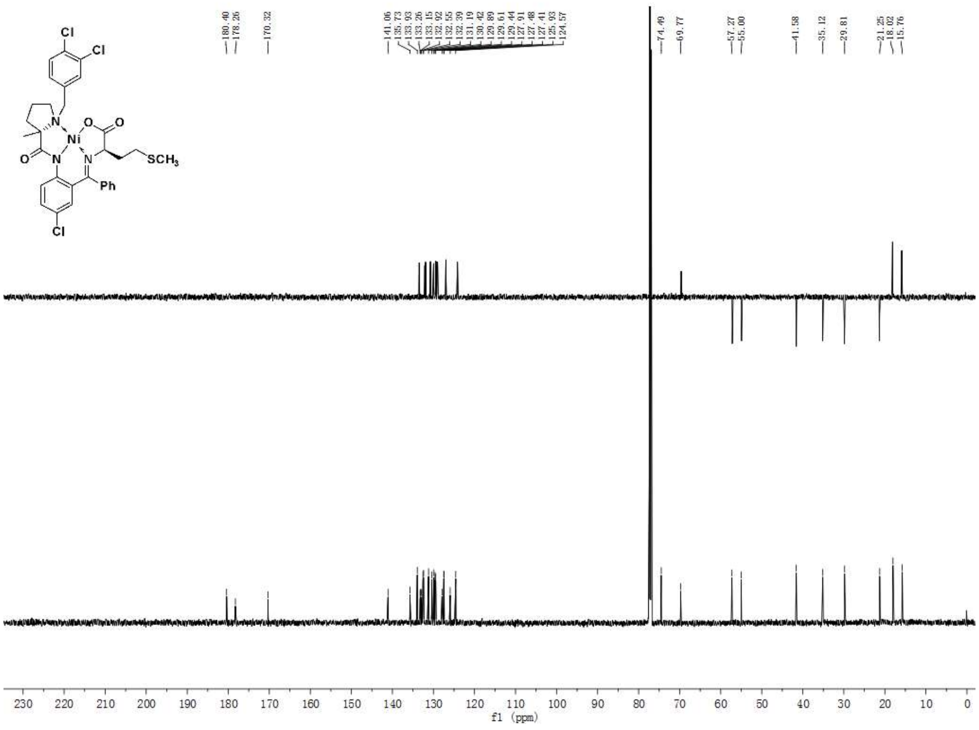

Figure S69: (*S*)-phenylalanine 5a

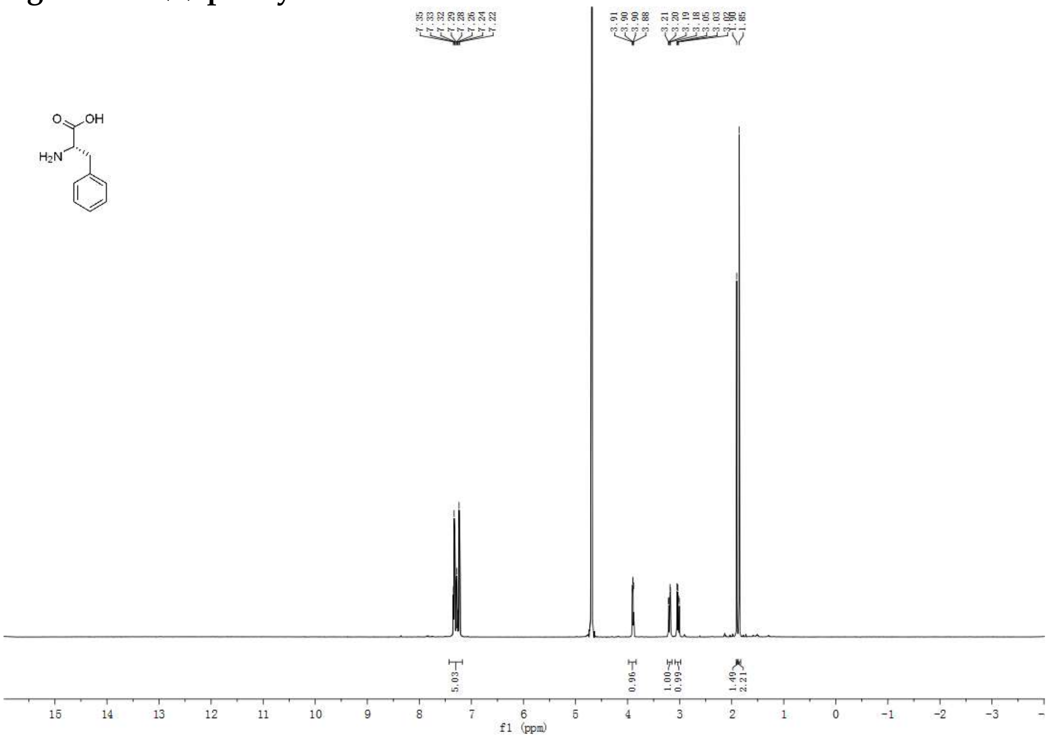

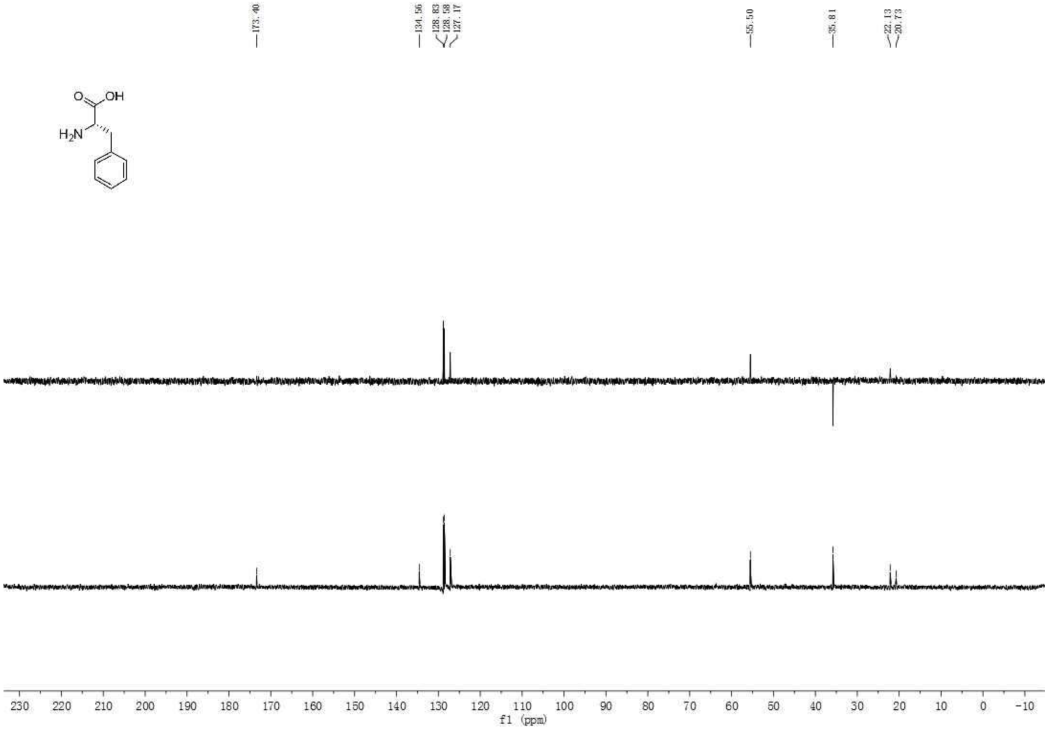

Supplement: Supplementary file 1 [file molecules-24-02218-s001.pdf]
